# Triple Combination of Entinostat, a Bromodomain Inhibitor, and Cisplatin Is a Promising Treatment Option for Bladder Cancer

**DOI:** 10.3390/cancers16193374

**Published:** 2024-10-02

**Authors:** Lukas M. Bollmann, Friedrich Lange, Alexandra Hamacher, Lukas Biermann, Linda Schäker-Hübner, Finn K. Hansen, Matthias U. Kassack

**Affiliations:** 1 Institute of Pharmaceutical and Medicinal Chemistry, Heinrich-Heine-University Duesseldorf, 40225 Duesseldorf, Germany; lukas.bollmann@hhu.de (L.M.B.);; 2 Department of Pharmaceutical and Cell Biological Chemistry, Pharmaceutical Institute, University of Bonn, An der Immenburg 4, 53121 Bonn, Germanyfinn.hansen@uni-bonn.de (F.K.H.)

**Keywords:** bladder cancer, resistance, epigenetics, class I HDACi, bromodomain inhibitor, entinostat, OTX015, JQ1, histone deacetylase (HDAC) inhibitor, cisplatin

## Abstract

**Simple Summary:**

The treatment of bladder cancer is still a challenge. New treatment options are required for patients not responding to established chemotherapy (e.g., cisplatin) due to primary or acquired chemoresistance. Bladder cancer cells are known for disturbed epigenetics contributing to resistance. This study aimed to investigate the combination of cisplatin, the class I histone deacetylase inhibitor (HDACi) entinostat, and bromodomain inhibitors (BETis) in two urothelial grade 3 bladder carcinoma cell pairs J82, cisplatin-resistant J82 cisR, T24, and cisplatin-resistant T24 LTT. Our results indicate that treatment of bladder cancer cells with entinostat and a BETi prior to cisplatin can completely reverse cisplatin resistance. This is of particular interest since entinostat and OTX015 (one of the used BETi) are already in clinical trials in other cancers. Thus, this study encourages further preclinical and eventually clinical trials with epigenetic inhibitors in combination with cisplatin to re-establish cisplatin efficacy in bladder cancer.

**Abstract:**

Background/Objectives. Cisplatin is part of the first-line treatment of advanced urothelial carcinoma. Cisplatin resistance is a major problem but may be overcome by combination treatments such as targeting epigenetic aberrances. Here, we investigated the effect of the class I HDACi entinostat and bromodomain inhibitors (BETis) on the potency of cisplatin in two pairs of sensitive and cisplatin-resistant bladder cancer cell lines. Cisplatin-resistant J82cisR and T24 LTT were 3.8- and 24-fold more resistant to cisplatin compared to the native cell lines J82 and T24. In addition, a hybrid compound (compound **20**) comprising structural features of an HDACi and a BETi was investigated. Results. We found complete (J82cisR) or partial (T24 LTT) reversal of chemoresistance upon combination of entinostat, JQ1, and cisplatin. The same was found for the BETis JQ35 and OTX015, both in clinical trials, and for compound **20**. The combinations were highly synergistic (Chou Talalay analysis) and increased caspase-mediated apoptosis accompanied by enhanced expression of p21, Bim, and FOXO1. Notably, the combinations were at least 4-fold less toxic in non-cancer cell lines HBLAK and HEK293. Conclusions. The triple combination of entinostat, a BETi, and cisplatin is highly synergistic, reverses cisplatin resistance, and may thus serve as a novel therapeutic approach for bladder cancer.

## 1. Introduction

Bladder cancer is the tenth most common cancer worldwide in both sexes, with men more commonly affected [1]. Further, bladder cancer is the most common malignant disease of the urinary tract [2]. Between 2020 and 2025, the incidence of bladder cancer is expected to increase across Europe through demographic change [3]. Cisplatin is used as standard therapy for urothelial carcinoma. For patients with advanced urothelial carcinoma, first-line treatment comprises cis- or carboplatin-based chemotherapy. For patients with muscle-invasive bladder cancer, neoadjuvant (chemotherapy before surgery) cisplatin-based chemotherapy is preferred over adjuvant treatment [4]. The development of cisplatin resistance is a common challenge [5,6]. Combination therapy is one way to overcome chemoresistance. For example, in advanced bladder cancer, a combination of cisplatin, gemcitabine, and paclitaxel has been studied. The combination therapy resulted in a prolonged survival of approximately 3 months, which was not statistically significant [7]. New effective combination therapies are thus urgently needed for the treatment of chemoresistant cancer cells to increase the effectiveness of chemotherapy and delay the problem of chemoresistance.

Bladder cancer shows epigenetic aberrances [8]. In addition, it is well known that aberrant histone and non-histone protein acetylation is a key determinant of acquired chemoresistance and, consequently, cisplatin resistance in cancer [9,10]. Epigenetic modulation, which describes reversible inhibition or activation of epigenetic writers, erasers, or readers, is one way of facing the chemoresistance problem [10]. Deacetylation by histone deacetylases (HDACs) of histones results in a condensed chromatin structure (heterochromatin), which suppresses gene expression. Histone deacetylase inhibitors (HDACis) can conversely be used to favor the open euchromatin structure and facilitate gene expression [11,12,13]. Epigenetic modulation of cancer cells by class I HDACi or pan-HDACi, followed by cisplatin treatment, demonstrated promising results in solid tumor cells [14,15,16,17]. Treatment with HDACi resulted in an increased expression of pro-apoptotic genes such as p21, APAF1, PUMA, and BAK1 and repression of anti-apoptotic genes such as survivin [14,18]. However, the sole use of class I HDACi or pan-HDACi is not a universal solution to the problem of chemoresistance because using a single epigenetic modifier such as an HDACi may not completely reverse cisplatin resistance, and additionally, cancer cells can also develop resistance to HDACi [17,19]. Therefore, further combination therapies need to be explored to overcome chemoresistance.

The bromodomain and extra-terminal domain inhibitors (BETis) containing bromodomains (BRDs) as an additional epigenetic target offer new potential for combination therapies [20]. BRD proteins can be divided into eight larger families, of which the BET proteins represent one family [21]. BET proteins orchestrate gene transcription by interacting with acetylated histones or transcription factors. In addition, BET proteins influence the JAK/STAT pathway as well as the cell cycle by activating oncogenes such as MYC, JUNB, CCND1, and CCNA1 [22,23,24,25]. As a result, BET proteins play a key role in cell survival and homeostasis. BET inhibitors (BETis) preferentially inhibit interaction with oncogenes that are essential for the survival of cancer cells [26,27,28]. A high sensitivity to BETi was discovered in chemoresistant ovarian tumors and patient-derived xenograft models with high MYC amplification. It was demonstrated that BETi had a synergistic effect in combination with cisplatin in ovarian cancer, even in cisplatin-resistant cells. Different explanations were given, including suppression of ALDH and downregulation of anti-apoptotic proteins BCL-2 and survivin. In addition, BETi sensitized ovarian cancer cells, which had a functional homologous recombination DNA repair pathway, to DNA damage by decreased expression of BRCA1 and RAD51 [18,25,29]. Modification of these signaling pathways by BETi may also lead to increased sensitivity to cisplatin in bladder cancer. Moreover, studies showed that the combination of the BET inhibitor JQ1 with the MAP kinase inhibitor trametinib in thyroid tumors resulted in a strong inhibition of tumor growth [30]. Furthermore, the BETi JQ1 suppressed cell proliferation by cell-cycle arrest and induction of apoptosis in acute lymphocytic leukemia cells [31]. Synergistic antiproliferative effects of BETi and cisplatin were demonstrated in malignant pleural mesothelioma cells via downregulation of MYC [32].

OTX015 is a BETi derived from JQ1. OTX015 has—in contrast to JQ1—an extended half-life and better oral bioavailability [27,33]. OTX015 revealed antitumor effects in vitro and in vivo against glioblastoma cells and non-small cell lung carcinoma cell lines [34,35]. Further, OTX015 has already been tested in clinical trials in hematopoietic and solid tumors. Dose-limiting toxicities in patients were anemia, thrombocytopenia, fatigue, and hyperbilirubinemia [27,36,37,38,39]. A clinical trial of OTX015 in solid tumors, such as midline carcinoma, was discontinued because part of the study indicated limited efficacy [40]. JQ35, another BETi, has also been tested in first clinical trials in acute myeloid leukemia and various solid tumors [41,42]. Further testing as a monotherapy for acute myeloid leukemia was discontinued due to a lack of efficacy [41].

Both HDACis and BETis influence gene expression. Therefore, a combination of BETis with HDACis may be useful to maximize the alteration of gene expression and for overcoming chemoresistance. The combination of HDACis and the BETi JQ1 has already been studied in acute myeloid leukemia, pancreatic ductal adenocarcinoma, glioblastoma, small-cell lung cancer, and bladder carcinoma, demonstrating synergistic anti-proliferative effects [28,43,44,45,46,47,48]. Furthermore, studies demonstrated that both HDACis and BETis downregulated the transcription of oncogenes and anti-apoptotic factors such as MYC or BCL-2 [28,48].

The aim of this study was to investigate a triple combination of HDACi, BETi and cisplatin in bladder cancer cells of different cisplatin sensitivity to reverse cisplatin resistance/increase cisplatin sensitivity and to increase the efficacy of treatment by exploiting synergistic effects of the combination. We investigated the combination treatment of the class I HDACi entinostat plus a BETi (JQ1 or JQ35 or OTX015) plus cisplatin in two bladder cancer cell pairs: native J82, cisplatin-resistant J82 cisR and native T24, and cisplatin-resistant T24 LTT (long-term treated). J82 cisR and T24 LTT were both established in laboratories of the University of Duesseldorf by intermittent treatment of the native cell lines with cisplatin as described in the literature [19,49,50]. J82 and T24 are muscle-invasive bladder cancer cell lines, both classified as grade 3 [49]. Muscle-invasive bladder cancer is treated with cisplatin-based combination chemotherapy in addition to radical removal of the bladder [2,31].

**Figure 1:**
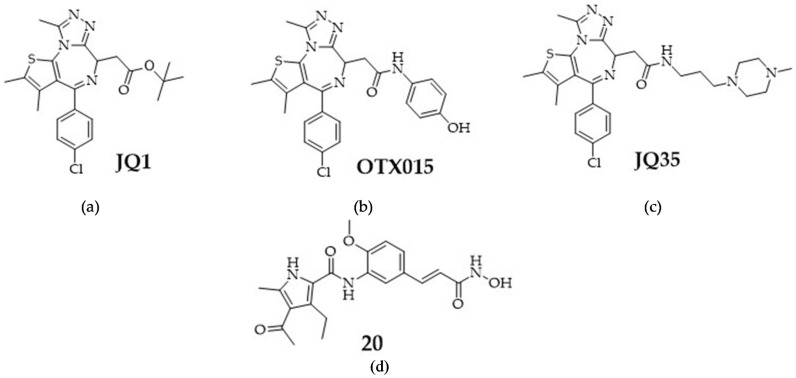
Structures of BETis: (**a**) JQ1, (**b**) OTX015, and (**c**) JQ35; (**d**) structure of dual HDACi and BETi **20 [51]**.

JQ1 was used as a model compound for BETis but has the disadvantage of a very short half-life in vivo and limited bioavailability [27,33]. Thus, we included OTX015 and JQ35 in our study, two BETis which are already used in a clinical trials (Figure 1) [27]. Additionally, we pursued a dual inhibitor approach to analyze the efficacy of combining an HDACi and a BETi in one molecule. Compound **20** combines structural features of and acts as an HDACi (class I/IIb) and BETi (Figure 1) [51].

Dual inhibitors have the advantage to simplify therapeutic application and may lead to higher efficacy by simultaneously inhibiting two targets by one molecule. We could demonstrate that triple combinations of entinostat, a BETi, and cisplatin act highly synergistically, and that entinostat and a BETi or compound **20** reversed cisplatin resistance while showing at least 4-fold selectivity for the bladder cancer over the non-cancer cell lines HBLAK and HEK293.

## 2. Materials and Methods

### 2.1. Materials

DMEM, penicillin/streptomycin (pen/strep) (10,000 U/mL; 10 mg/mL), fetal calf serum (FKS), and trypsin-EDTA (0.05% trypsin, 0.02% EDTA in phosphate-buffered saline) were received from PAN-Biotech (Aidenbach, Germany). Cnt-Prime Medium was obtained from CELLnTEC (Bern, Switzerland). Entinostat was ordered from Selleckchem (Houston, TX, USA). The JQ1 was purchased from Tocris (Bristol, UK). The OTX015 and JQ35 were delivered by AdooQ (AdooQ Bioscience, Irvine, CA, USA). The compound **20** was synthesized by Dr. Finn Hansen, Institute of Pharmaceutical and Cell Biological Chemistry, University of Bonn. The cisplatin was supplied by Sigma-Aldrich (Steinheim, Germany) and dissolved in 0.9% sodium chloride solution and further diluted with 0.9% sodium chloride. All other compounds were dissolved in the DMSO to a concentration of 10 mM. All further dilutions were prepared with the appropriate culture medium. A maximum amount of 1% DMSO was used in all experiments. Triton X-100 was delivered by AppliChem (Darmstadt, Germany), and propidium iodide was purchased from Santa Cruz Biotechnology (Heidelberg, Germany).

### 2.2. Cell Lines and Cell Culture

The human urothelial bladder carcinoma cell line J82 and the non-cancer cell line HEK293 were purchased from DSMZ (Braunschweig, Germany). The cisplatin-resistant subclone J82 cisR was created by repetitive treatment with cisplatin, as previously described [19]. The primary epithelial cell line HLBAK [52], T24, and T24 LLT cell line were kindly provided by Dr. Michèle Hoffmann, Department of Urology, Heinrich-Heine-university, Duesseldorf. The cell lines J82, J82 cisR, T24, T24 LTT, and HEK293 were cultured with DMEM, where 120 IU/mL penicillin and 120 µg/mL streptomycin and 10% heat-inactivated fetal calf serum were added. For cultivation of the HBLAK cells, the culture medium Cnt-Prime was used without further additives. All cells were incubated at 37 °C, 5% CO_2_ under humidified air. The J82 cisR and T24 LTT showed stable cisplatin resistance and did not require permanent treatment with cisplatin to maintain the cisplatin resistance.

### 2.3. MTT Cell Viability Assay

The cell viability for determination of IC_50_ values and synergism studies was analyzed by MTT assay as previously described [19,53]. Briefly, cells were incubated with the indicated compounds for 72 h or 48 h pre-incubated plus 72 h in combination with cisplatin. For preincubation experiments, 1600 c/w (cells per well) for J82 and 1200 c/w for J82 cisR were used. After the incubation time, 25 µL MTT (3-(4,5-dimethylthiazol-2-yl)-2,5-diphenyltetrazolium bromide, Serva, Heidelberg, Germany) solution (5 mg/mL in phosphate-buffered saline) was added. The formazan was dissolved in 75 µL DMSO, and the absorption was measured at 540 nm and 690 nm with a microplate reader (ThermoFisher Multiskan, Thermo Scientific, Wesel, Germany).

### 2.4. Measurement of Apoptotic Nuclei

The amount of apoptotic nuclei with DNA content in sub-G1 was measured as described before [19]. Briefly, J82 (75.000 c/w), J82 cisR (60.000 c/w), T24 (45.000 c/w), and T24 LTT (90.000 c/w) cells were seeded in 6-well plates (Sarstedt, Nümbrecht, Germany). Cells were 48 h preincubated with entinostat and JQ1 or OTX015. Then, the culture medium was removed, and the cells were incubated additional 24 h with entinostat, JQ1 or OTX015 and cisplatin. After the incubation, the 6-well plates were centrifuged, and the supernatant was removed. Cells were lysed by adding 1 mL hypotonic lysis buffer (0.1% tritonX-100, 100 µg/mL propidium iodide) at 4 °C in the dark overnight. The lysed cells were analyzed by flow cytometry using the CyFlow instrument (Partec, Norderstedt, Germany).

### 2.5. Activation of Caspase 3/7

Activation of caspase 3/7 was examined using the CellEvent Caspase-3/7 green detection reagent (Thermo Scientific, Wesel, Germany) according to the manufacturer’s instructions. The cells were seeded in 96-well-plates (Corning, Kaiserslautern, Germany) and treated with the indicated compounds for 48 h (J82 1600 c/w; J82 cisR 800 c/w; T24 1200 c/w; T24 LTT 3000 c/w). Afterwards, the medium was removed, and fresh medium and compounds including cisplatin were given to the cells for 24 h. The supernatant was aspirated and a solution of CellEvent Caspase 3/7 green detection reagent (PBS, 1% heat inactivated fetal calf serum) was added to the cells for a final concentration of 2 µM. Hoechst 33342 (10 mg/mL in water, Sigma-Aldrich, Steinheim, Germany) was used for nuclei staining. The cells were incubated for 30 min at 37 °C and 5% CO_2_ before imaging by using the Thermo Fischer ArrayScan XTI high content screening (HCS) system with a 10 × magnification (Thermo Scientific, Wesel, Germany).

### 2.6. Immunoblotting

Protein extraction and Western blot analysis were performed as previously described with minor modifications [19]. The cells were incubated for 48 h with the indicated concentrations. The medium was aspirated, and the cells were washed with PBS 1X after the incubation period. Then, a RIPA buffer (50 mM Tris-HCl pH 7.4, 1% NP-40, 0.5% sodium deoxycholate, 0.1% SDS, 150 mM sodium chloride, 2 mM EDTA, spiked with protease and phosphatase inhibitor (Pierce protease and phosphatase inhibitor mini tablets, Thermo Scientific, Wesel, Germany)) was added, and the cells were removed using a cell scraper. The cells were lysed for 30 min at 4 °C with agitation. Then, the protein preparations were boiled at 95 °C with 2x Laemmli buffer containing β-mercaptoethanol for 3 min. Pierce BCA protein assay was used to determine the total amount of protein in the protein preparations (Thermo Scientific, Wesel, Germany). Equal amounts of protein were separated by SDS-PAGE and transferred to a polyvinylidene fluoride membrane (Merck Millipore, Darmstadt, Germany). PageRuler Prestained Ladder, 10 to 180 kDa, or 10 to 250 kDa (Thermo Scientific, Wesel, Germany) were used as a size comparison marker. Blots were incubated with the primary antibodies (Table S1). The secondary antibodies conjugated to HRP used in the Western blot were supplied by bio-techne (Minneapolis, MN, USA). Proteins were visualized using Luminol reagent (Santa Cruz Biotechnology, Heidelberg, Germany) in Intas Imager (Intas, Göttingen, Germany). The images were analyzed with ImageJ 1.54g (http://imagej.org).

### 2.7. RT-PCR

The cells were incubated with the indicated concentrations of entinostat and JQ1 for 48 h. RNA was isolated using the RNeasy Mini Kit (Qiagen, Hilden, Germany). Subsequently, the RNA was transcribed into cDNA using the High-Capacity cDNA Reverse Transcription Kit (Thermo Scientific, Wesel, Germany). For an RT-PCR, GoTaq qPCR Master Mix (Promega, Fitchburg, WI, USA) and a CFX96 Real-Time System (BIO-RAD, Hercules, CA, USA) were used. The relative changes in gene expression were normalized to the control genes *GUSB* (beta-glucuronidase), *TBP* (TATA binding protein), and *HPRT1* (hypoxanthine–guanine phosphoribosyltransferase) by Vandesompele method [54]. Primers were designed using Primer-BLAST (NIH, Bethesda, MD, USA) and had an efficiency between 80% and 115% (Table S2). Efficiency was determined using an isolated template of HeLa cells.

### 2.8. Analysis of Double Strand Breaks with γ-H2AX and 53BP1

An analysis of double-strand breaks measuring γ-H2AX and 53BP1 foci was performed according to the literature, with minor modifications [55]. The J82 (60.000 c/w) and J82 cisR (48.000 c/w) were seeded on a coverslip (Paul Marienfeld GmbH & Co.KG, Lauda Königshofen, Germany) in a 6-well plate (Sarstedt, Nümbrecht, Germany). The cells were treated for the indicated time. Then, the cells were fixed with 3% formaldehyde for 10 min. The cells were permeabilized with 0.3% Triton X-100 in PBS and then blocked with 5% BSA for one hour. After this step, the primary antibody incubation (anti-phospho-Histone H2A.X Ser139 clone JBW301, 05-636, Merck Millipore, Darmstadt, Germany; 53BP1 #4937, Cell Signaling Technology, Danvers, Massachusetts, USA) was carried out overnight at 4 °C. The next day, the secondary antibody incubation was performed for one hour (AlexaFluor 555 and AlexaFluor 647, Thermo Scientific, Wesel, Germany). The cells on the coverslips were mounted in a DAPI-containing mounting medium (UltraCruz Mounting Medium, Santa Cruz Biotechnology, Heidelberg, Germany) and fixed with nail varnish. The evaluation was carried out on a Leica Thunder microscope (Leica Mikrosystems, Wetzlar, Germany).

### 2.9. Enzyme HDAC Inhibition Assay

The human recombinant enzymes were ordered from Reaction Biology Corp (Malvern, PA, USA). To determine the inhibitory activity of the compounds, the enzymes HDAC 2 (cat. no. KDA-21-277), HDAC 4 (cat. nr. KDA-21-279), HDAC 6 (cat. no. KDA-21-213), and HDAC 8 (cat. no. KDA-21-481); with 20 ng of HDAC 2 and 8, 17.5 ng of HDAC 6, and 2 ng of HDAC 4 per well were used. After pipetting 10 µL of increasing concentrations of the inhibitors to a 96-well plate (Corning, Kaiserslautern, Germany), the recombinant enzymes were diluted in assay buffer (50 mM Tris-HCl, pH 8.0, 137 mM KCl, 1 mM MgCl2, and 1 mg/mL bovine serum albumin (BSA) and added to the wells. After five minutes of incubation, the reaction was started with 10 µL of a 30 µM solution of Boc-Lys(Ac)-AMC (Bachem, Bubendorf, Switzerland) for HDAC 2 or a 15 µM solution for HDAC 6. For the HDAC 4, a 10 µM solution of Boc-Lys-(Tfa)-AMC (Bachem, Bubendorf, Switzerland) was used, and for the HDAC 8, a 6 µM solution was used. After 90 min, the reaction was stopped by adding 100 µL of stop buffer (16 mg/mL trypsin and 4 µM vorinostat for HDAC 2, 4 µM panobinostat for HDAC 4 and HDAC 8 and 4 µM tubastatin A for HAC 6 in 50 mM Tris-HCl, pH 8.0 and 100 mM NaCl). After additional 15 min, fluorescence intensity was measured at an excitation wavelength of 355 nm and an emission wavelength of 460 nm in the NOVOstar microplate reader (BMG LabTech, Offenburg, Germany).

### 2.10. Data Analysis

The concentration–effect curves were created with Prism 8.0.2 (GraphPad, San Diego, CA, USA). The statistical analysis was performed using two-tailed unpaired *t*-test in GraphPad Prism. To determine the synergistic effect, the sum of the single treatments was calculated with standard deviation. This value was compared with the measured value of the combination treatment through an unpaired *t*-test. CI-values were calculated according to Chou-Talalay using compuSyn 1.0 software [56,57]. Synergy levels were calculated applying the Bliss Model with combenefit [58,59].

## 3. Results

### 3.1. Cytotoxic Activity and Synergism Studies of Entinostat, JQ1, and Cisplatin

First, the IC_50_ values of entinostat, JQ1, and cisplatin were estimated in all bladder cancer cell lines by an MTT assay. Concentration–effect curves are shown in Figure S1, IC_50_ values are displayed in Table 1.

Two pairs of cisplatin-sensitive and cisplatin-resistant bladder cancer models were selected: J82, J82 cisR; and T24, T24 LTT. The J82 cisR showed a moderate cisplatin-resistance factor of 3.8. For the T24 LTT, the resistance factor was 24, demonstrating very high cisplatin resistance. Entinostat had similar IC_50_ values in J82 and J82 cisR, but a 4-fold lower IC_50_ in T24 LTT than T24. Interestingly, both cisplatin-resistant bladder cancer cell lines were more sensitive to JQ1 than the parental cell lines. J82 demonstrated an 8.4-fold lower sensitivity towards JQ1 than J82 cisR. The same observation was made for T24 with a 4-fold lower sensitivity than T24 LTT (Table 1). For further investigations, concentrations of entinostat and JQ1 were chosen which are achievable in in vivo studies according to the literature data. Furthermore, entinostat and BETi were used in combination studies in a maximum concentration reflecting their IC_25_, respectively, thus showing low toxicity when given alone [27,60].

Then, the mutual effect of the two epigenetic modulators was tested: the effect of a low concentration (maximum IC_25_) of the BETi JQ1 on the IC_50_ of entinostat (Figure S2a,c) and the effect of a low concentration (maximum IC_25_) of the HDACi entinostat on the IC_50_ of JQ1 (Figure S2b,d) in J82 and J82 cisR. Regardless of a 72 h co-incubation of both inhibitors or a sequential treatment (48 h pre-incubation of the IC_25_ of one inhibitor followed by addition of the second inhibitor for another 72 h), no significant increase in the potency of either epigenetic modulator was found (Figure S2 and Table S3). Shift factors were calculated by dividing the IC_50_ value of the control treatment with the IC_50_ value of the treated cells. All shift factors were below 2 (Table S3).

**Figure 2:**
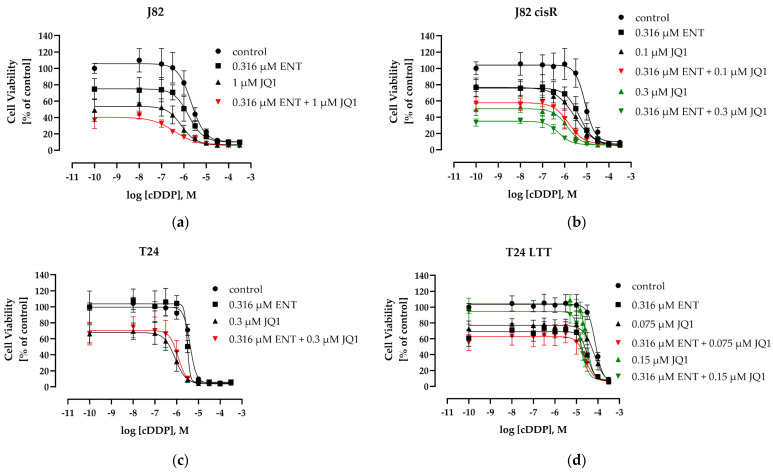
The concentration–effect curves of cisplatin (cDDP) (MTT assay). The cells were preincubated with entinostat (ENT) and/or JQ1 for 48 h followed by the addition of cisplatin for another 72 h. (**a**) J82, (**b**) J82 cisR, (**c**) T24, and (**d**) T24 LTT. Control means concentration–effect curve of cisplatin in the absence of epigenetic inhibitors. Data shown are the mean ± SD of at least three independent experiments, each carried out in triplicates.

Next, the effect of each epigenetic modulator alone and of the combination of entinostat plus JQ1 on the IC_50_ of cisplatin was studied. A 72 h co-incubation of entinostat, JQ1, and cisplatin did not achieve a significant increase in the potency of cisplatin. Shift factors of cisplatin were less than 2, except in J82 cells (Figure S3, Table S4). We then used our proven incubation scheme, applying a 48 h preincubation of the epigenetic inhibitors prior to addition of cisplatin for another 72 h, allowing the epigenetic inhibitors to modulate the cellular epigenetic environment prior to addition of the cytotoxic compound. This incubation scheme has proven successful in several of our previous studies [14,61]. Notably, whereas co-incubation of entinostat, JQ1, and cisplatin did not increase the potency of cisplatin (Figure S3, Table S4), preincubation of entinostat and JQ1 for 48 h followed by the addition of cisplatin for another 72 h increased the potency of cisplatin up to 15.6-fold. The concentration–effect curves and corresponding IC_50_ values with shift factors are displayed in Figure 2 and Table 2.

The 48 h preincubation with entinostat gave only shift factors (SFs) of <2 for cisplatin, except for T24 LTT cells (SF 2.26) whereas preincubation with JQ1 gave SF > 2, except for 0.075 µM JQ1 in T24 LTT where cisplatin remained almost equipotent with or without JQ1 preincubation (Table 2). Preincubation with both epigenetic modifiers (entinostat plus JQ1) increased cisplatin potency more than with one epigenetic modifier only, except for T24 cells, where JQ1 alone gave an SF of 4.92 and entinostat plus JQ1 gave an SF of 3.52. Cisplatin resistance was completely reversed for the J82 cisR cells. Even in the highly resistant T24 LTT cell line, a 4.29-fold resensitization against cisplatin could be achieved. Since 48 h preincubation of the epigenetic modifiers prior to cisplatin addition was more successful than co-incubation of all three compounds, all following experiments were performed using preincubation conditions.

**Figure 3:**
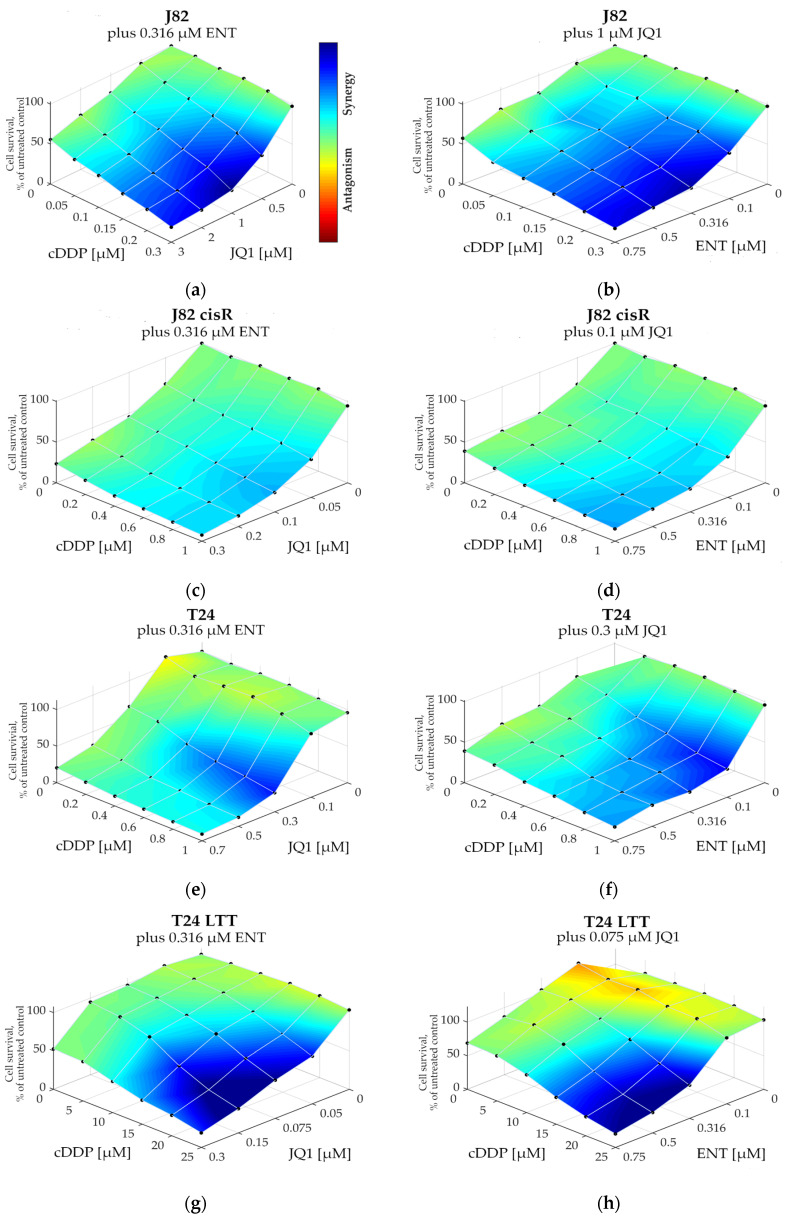
Synergy levels calculated applying the Bliss model using combenefit [58] of the triple combination studies (48 h preincubation with entinostat and JQ1, followed by addition of cisplatin for another 72 h). Entinostat was used at 0.316 µM for all cell lines. JQ1 was used at 1 µM in J82, 0.1 µM in J82 cisR, 0.3 µM in T24, and 0.075 µM in T24 LTT. (**a**,**b**) J82, (**c**,**d**) J82 cisR, (**e**,**f**) T24, and (**g**,**h**) T24 LTT.

Next, synergism studies were carried out for all cell lines for dual combinations (JQ1 plus cisplatin; entinostat plus cisplatin) and for triple combinations (48 h preincubation of entinostat plus JQ1 followed by addition of cisplatin for another 72 h). Data were analyzed according to Chou-Talalay [57] (Table 3) and according to the Bliss model [58] (Figure 3). Dual combinations were not or only moderately synergistic (Table S5), particularly with entinostat, as displayed by color coding: red color: non-synergistic, green color: synergistic. The only dual combination displaying strong synergy was JQ1 with cisplatin in T24 cells, showing CI values < 0.9 (indicating synergism) for almost all chosen concentrations (Table S5) which is in accordance with the high SF for cisplatin of 4.92 (Table 2). Further, in T24, the synergy/CI values of the dual combination (JQ1 plus cisplatin) and of the triple combination (entinostat, JQ1, cisplatin) were comparable (Table 3 and Table S5).

Triple combinations were analyzed by Chou-Talalay in the following manner: one of the epigenetic modifiers was used at a fixed concentration, whereas the other and cisplatin were used in increasing concentrations. Thus, two data sets were obtained for each cell line (Table 3). In accordance with the previously determined shift factors (Table 2), the strongest synergism was observed in the J82, J82 cisR, and T24 cells (Table 3).

It is remarkable that the preincubation of entinostat plus JQ1 at low-toxic concentrations (0.316 µM entinostat plus 1 µM JQ1 in J82 cells; 0.316 µM entinostat plus 0.1 µM JQ1 in J82 cisR) already achieved a synergistic effect at 0.05 µM cisplatin for J82 and at 0.2 µM cisplatin for J82 cisR (Table 3). Similar data were observed in T24 at a cisplatin concentration of 0.2 µM and in the highly cisplatin-resistant T24 LTT cell line at 15 µM cisplatin.

Using combenefit, the synergy levels were calculated according to the Bliss model (Figure 3). Again, a clear synergistic effect was observed for the triple combination in all cancer cell lines.

In addition, the synergy data from MTT assays were further evaluated as a sum of the cytotoxic effects of the respective single compound treatments (black bars) compared to the cytotoxic effect of the combination treatments (white bars), as shown in the supplement (Figure S4). For almost all concentrations in all cell lines, the sum of the single treatment effects (black bars) was significantly lower than the effects of the triple combination treatments (white bars), supporting the synergistic behavior of the combination treatments (Figure S4).

### 3.2. Triple Combination Treatment with Entinostat, JQ1, and Cisplatin Enhances Apoptosis Induction Via Caspase-3/7 Activation and Double-Strand Breaks

**Figure 4:**
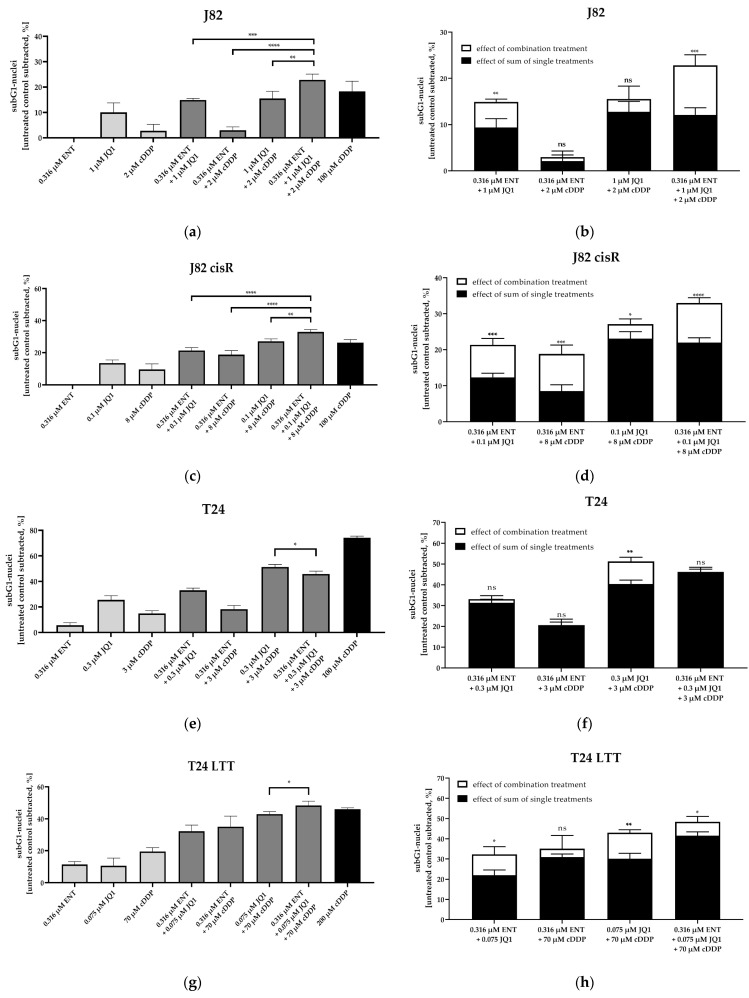
The estimation of apoptosis by measuring induction of the subG1-nuclei. The cells were preincubated with entinostat and JQ1 for 48 h followed by addition of cisplatin for another 24 h. Apoptotic cells were defined by subG1 fraction (flow cytometry). Data shown are the mean + SD of one representative experiment out of at least two, each carried out at least in duplicates. Untreated control was subtracted. (**a**,**b**) J82; (**c**,**d**) J82 cisR; (**e**,**f**) T24; (**g**,**h**) T24 LTT. (**a**,**c**,**d**,**e**) show subG1 nuclei. (**b**,**d**,**f**,**h**) show the comparison between the sum of the single compound effects (black bars) versus the effect of the combination treatment (white bars). Statistical analysis was performed using unpaired *t*-test. Level of significance: ns (*p* ≥ 0.05); * (*p* < 0.05); ** (*p* < 0.01); *** (*p* < 0.001); **** (*p* < 0.0001).

Subsequently, we investigated whether the synergistic effect of the triple combination was based on an increased induction of caspase-mediated apoptosis. First, the induction of subG1 cell nuclei was measured using flow cytometry (Figure 4). In total, 100 µM of cisplatin was used as positive control for J82, J82 cisR, and T24. In total, 200 µM of cisplatin was chosen as positive control for T24 LTT, based on the high cisplatin resistance of these cells. Representative cytometry flow images are shown in Figure S5.

The triple treatment led to the highest induction of subG1 nuclei in J82, J82 cisR, and T24 LTT. In T24, the dual combination consisting of JQ1 and cisplatin had the strongest effect, followed by the triple treatment. This was consistent with the SF in Table 2 (JQ1 and cisplatin: SF 4.92 vs. entinostat, JQ1, and cisplatin: SF 3.52). Statistical evaluation demonstrated that the triple combination (white bars) caused a significantly higher increase in subG1 nuclei than the sum of the single treatments (black bars) in J82, J82 cisR and T24 LTT. This emphasizes the synergistic effect of the triple combination. Again, T24 was outstanding, as the dual combination of JQ1 and cisplatin already had a significant synergistic effect, which was not further enhanced by a triple combination. These results confirmed the previous ones (Section 3.1) and suggest enhanced apoptosis by the triple combination in at least three cell lines.

**Figure 5:**
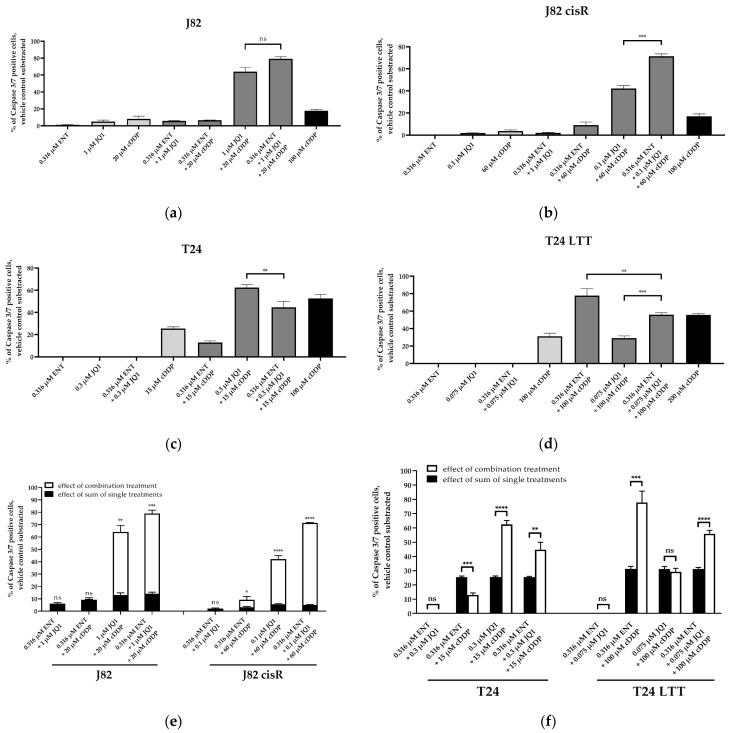
Caspase 3/7 activation in (**a**) J82, (**b**) J82 cisR, (**c**) T24, and (**d**) T24 LTT. The cells were preincubated with entinostat and JQ1 for 48 h followed by addition of cisplatin for another 24 h (T24 LTT 48 h). (**e**,**f**) show the comparison between the sum of the single-compound effects (black bars) versus the effect of the combination treatments (white bars) in J82 and J82 cisR (**e**) and T24 and T24 LTT (**f**). Data shown are the mean + SD of one representative experiment out of at least two, each carried out in duplicates. Significance was calculated using unpaired *t*-test. Level of significance: ns (*p* ≥ 0.05); * (*p* < 0.05); ** (*p* < 0.01); *** (*p* < 0.001); **** (*p* < 0.0001).

To examine whether apoptosis was caspase-mediated, caspase 3/7 activation was analyzed (Figure 5). Representative fluorescence images of each cell line are shown in Figure S6. Again, 100 µM of cisplatin was chosen as positive control for caspase 3/7 activation in J82, J82 cisR, and T24. Due to the high cisplatin resistance of the T24 LTT cells (Table 1), 200 µM of cisplatin were incubated in the T24 LTT cells for 48 h in contrast to the other cancer cell lines where 100 µM of cisplatin was used for 24 h.

**Figure 6:**
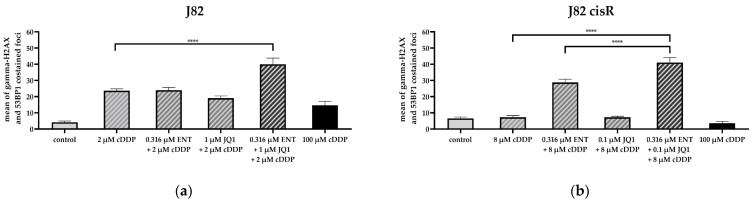
Number of foci stained for γ-H2AX and 53BP1 of (**a**) J82 and (**b**) J82 cisR. Cells were preincubated with entinostat and JQ1 for 48 h followed by addition of cisplatin for another 24 h. Foci in at least 19 representative random cells were counted. Data shown are the mean + SEM of counted foci. Significance was calculated using unpaired *t*-test in GraphPad Prism. Level of significance: ns (*p* ≥ 0.05); **** (*p* < 0.0001).

**Figure 7:**
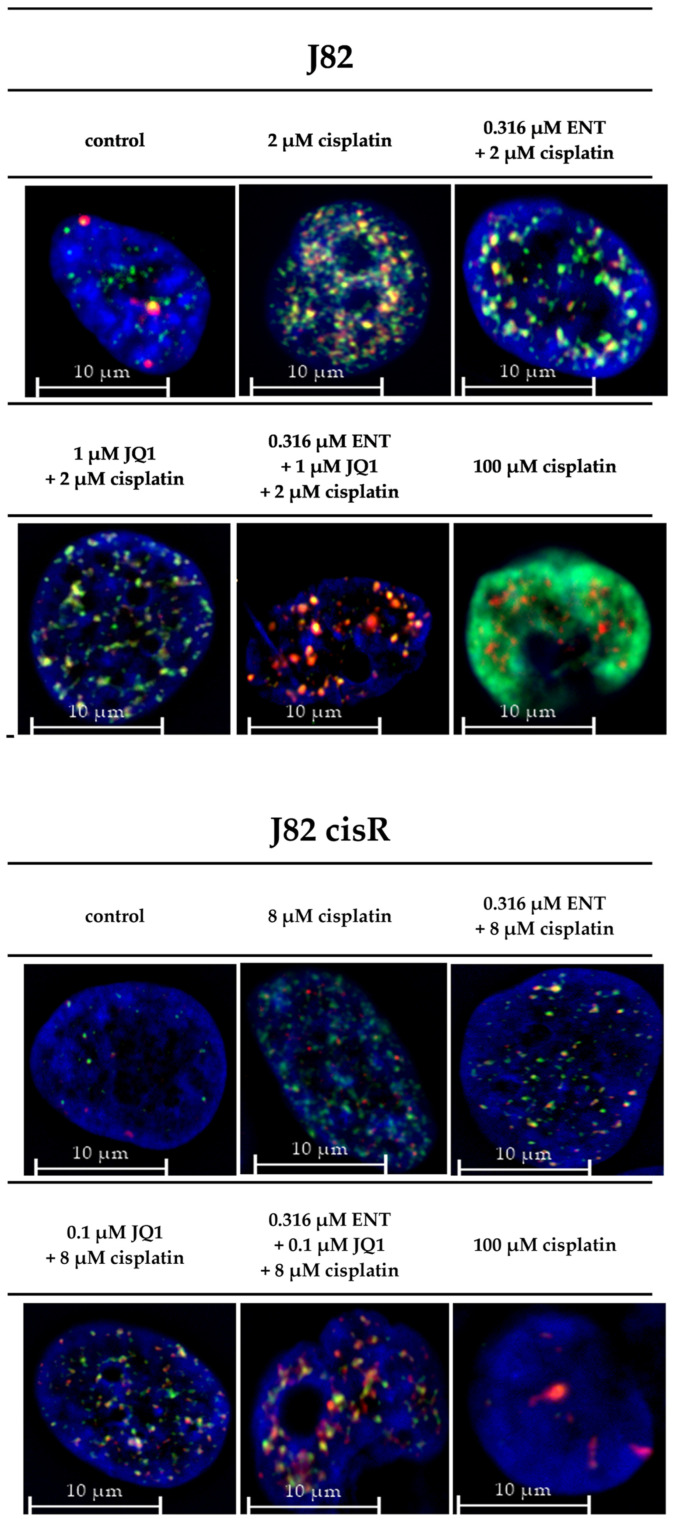
One representative fluorescence image out of five experiments is shown for each treatment condition for J82 and J82 cisR (γ-H2AX (green) and 53BP1 (red); overlay of γ-H2AX and 53BP1 (yellow)). The nuclei were stained using Hoechst 33342 (blue). The 100 µM of cisplatin in J82: DAPI staining is overlayed by strong γ-H2AX staining. The cells were preincubated with entinostat and JQ1 for 48 h, followed by addition of cisplatin for another 24 h.

The triple treatment caused the highest caspase 3/7 activation in J82 and J82 cisR. T24 cells revealed the strongest caspase 3/7 activation with the combination of JQ1 and cisplatin followed by the triple combination. T24 LTT exhibited the greatest caspase 3/7 activation with the combination of entinostat and cisplatin (Figure 5). This is in agreement with a reduction in the IC_50_ value of cisplatin in the dual combination entinostat plus cisplatin, which was in a similar range as the reduction in the IC_50_ value with the triple combination (Table 2). Nevertheless, the triple combination led to a clear increase in caspase 3/7 activation. Further, comparing the sum of the caspase activation of the single treatments (black bars) with the caspase activation of the combination treatments (white bars), we could demonstrate that the triple treatment is synergistic in all cell lines, i.e., the white bars are larger than the black bars. This was also true for the dual combination of JQ1 and cisplatin in the J82, J82 cisR, and T24 cells, and for the dual combination of entinostat and cisplatin in the T24 LTT cells. Overall, these results were in accordance with the apoptosis experiments (Figure 4). Additionally, we could confirm that the increased apoptosis is caspase 3/7-mediated. A further mechanistic investigation is the analysis of compound-induced double-strand breaks as additional readout for the cytotoxic effect. The number of corresponding foci of γ-H2AX and 53BP1 was counted as surrogate parameters for double-strand breaks using fluorescence microscopy (Figure 6) [62,63,64]. Because γ-H2AX could also be generated by other cellular processes, 53BP1 was used as an additional marker [65,66]. The presence of both proteins at the same position is considered as a specific indication of double-strand breaks [67,68,69]. Representative fluorescence images of J82 and J82 cisR cells are shown in Figure 7.

Treatment with the triple combination yielded the highest number of co-staining foci of γ-H2AX and 53BP1 in the J82 and J82 cisR cells (Figure 6 and Figure 7). This increase was significant compared to all other treatments underlining the strongest effect for a triple combination treatment. Dual combinations were not more potent in increasing double strand breaks compared to cisplatin treatment only, except for the combination of entinostat or JQ1 and cisplatin in J82 cisR cells. The 100 µM cisplatin showed only a small number of double-strand breaks in both cell lines, especially in J82 cisR, which might be due to an advanced stage of apoptosis due to the high cisplatin concentration. The total number of double-strand breaks was lower in J82 cisR than in J82 when treated with cisplatin alone and in combination with JQ1 and cisplatin. Generally, this could be related to an increased DNA repair capacity in J82 cisR contributing to cisplatin resistance. In summary, we could demonstrate synergy for the triple combination in caspase-mediated apoptosis and a significantly stronger effect in inducing double strand breaks.

### 3.3. Effect of Entinostat and JQ1 on Expression of Pro- and Anti-Apototic Genes and Proteins

**Figure 8:**
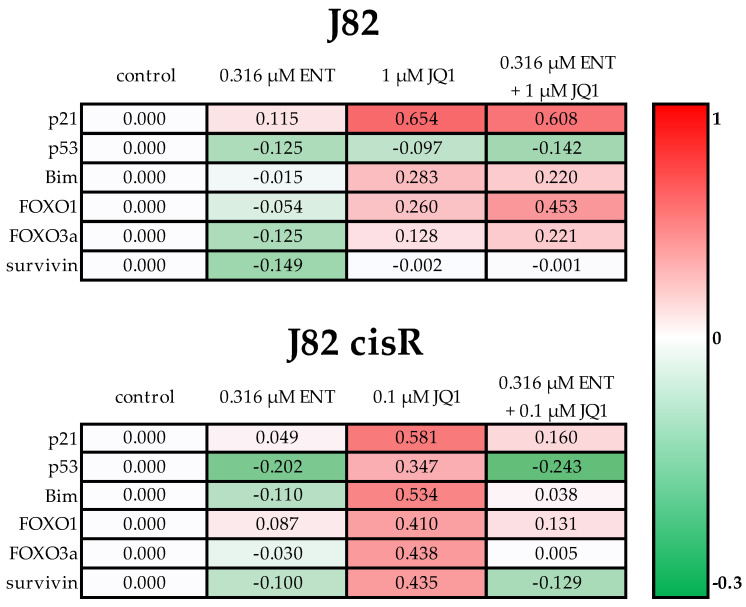
Gene expression was examined by RT-qPCR. Cells were incubated with entinostat and JQ1 for 48 h. The analysis was performed according to Vandesompele [54]. Shown is the decadic logarithm of the expression values normalized to the expression of the control genes GUSB (beta-glucuronidase), TBP (TATA-binding protein), and HPRT1 (hypoxanthine–guanine phosphoribosyltransferase) of the respective cell line. Negative values (green) indicated lower gene expression, and positive values (red) indicated higher gene expression compared to the cell-specific control. Data are shown of one representative experiment carried out in duplicates.

Next, the effects of the epigenetic modulators entinostat and JQ1 on gene and protein expression were analyzed to determine the effect of their preincubation. Therefore, the influence of entinostat, JQ1, and their combination was investigated on the expression of pro- and anti-apoptotic genes in the J82 and J82 cisR cells, showing the most favorable results with the triple combination. The RT-qPCR was performed after 48 h incubation with entinostat and JQ1 and evaluated according to Vandesompele [54]. Genes coding for pro-(Bim, FOXO1, FOXO3a) and anti-apoptotic (survivin) proteins were analyzed. Furthermore, gene expression of proteins that influence the cell cycle (p21, p53) were also examined (Figure 8).

In J82 and J82 cisR, *p21* was only slightly upregulated by treatment with entinostat. In contrast, JQ1 or the combination of entinostat and JQ1 led to a greater upregulation of *p21* in both cell lines. In J82, the increase in p21 gene expression by the triple treatment was only slightly weaker than by JQ1 alone, whereas the increase in p21 was 3.6-fold greater by JQ1 than by the combination of entinostat and JQ1 in J82 cisR. *P53* was downregulated by the epigenetic modulators in J82 and J82 cisR, except for JQ1 alone in J82 cisR, where *p53* was upregulated. *Bim*, *FOXO1,* and *FOXO3a* showed an increased gene expression after JQ1 and the dual combination treatment in both cell lines, while *Bim* and *FOXO3a* were only marginally upregulated through the combination treatment in J82 cisR. The gene expression of *survivin* was inconsistent. *Survivin* was less expressed by entinostat in J82. The treatment with JQ1 or the combination of entinostat and JQ1 hardly made any difference to control. In J82 cisR, *survivin* was strongly upregulated by JQ1, but downregulated by entinostat and the combination treatment.

**Figure 9:**
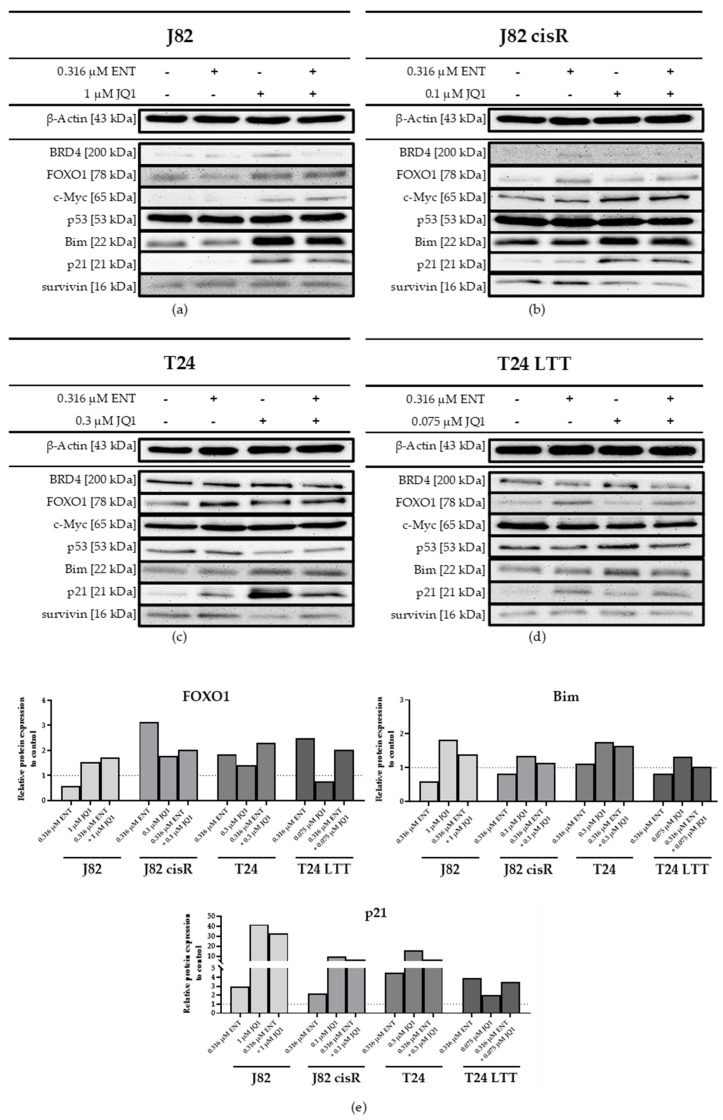
Western blot analysis of (**a**) J82, (**b**) J82 cisR, (**c**) T24, and (**d**) T24 LTT. The cells were incubated with entinostat and JQ1 for 48 h. The data shown are a representative blot out of at least two different protein samples. The uncropped blots are presented in the supplement (Figures S8–S14). (**e**) Relative protein expression of FOXO1, Bim, and p21 related to β-actin as loading control in J82, J82 cisR, T24, and T24 LTT. The ratio of control was set to 1, represented by the dashed line. Shown Western blots were analyzed with ImageJ. The analysis of the remaining proteins is presented in Figure S7.

To verify the results of the RT-qPCR at the protein level, Western blots were performed with the same incubation time. Furthermore, BRD4 and c-Myc were considered as additional target proteins of JQ1. JQ1 has previously been shown to exert its antitumor effects by inhibiting c-Myc expression [70]. The presented blots were analyzed with ImageJ for a more comprehensive analysis. The results are shown in Figure 9 and Figure S7.

The combination of entinostat and JQ1 revealed an increased protein expression of FOXO1, Bim, and p21 compared to the control in all four cell lines (Figure 9). The strongest increase in FOXO1 expression was induced by entinostat in J82 cisR and T24 LTT and not by the combination treatment. Interestingly, Bim was most expressed when treated with JQ1 alone, but the combination of entinostat and JQ1 gave similar results. P21 was highly upregulated by JQ1 and the combination treatment in J82, J82 cisR, and T24. In T24 LTT, p21 was also upregulated, but only by entinostat and the combination treatment. A slight decrease in survivin was only evident with JQ1 and the combination treatment in J82 cisR and T24. P53 was downregulated by the combination of entinostat and JQ1 in all four cell lines. C-Myc was detectable in all cell lines, tending towards upregulation in J82 and J82 cisR and downregulation in T24 and T24 LTT (Figure S7) upon treatment. BRD4 was only very slightly expressed in J82 cisR in contrast to the other three cell lines. The present Western blot results were largely consistent with the qPCR results, demonstrating the preincubation with both epigenetic modulators resulted in upregulation of p21, FOXO1, and Bim.

### 3.4. Investigation of BETi OTX015, JQ35, and Dual Inhibitor 20 in Combination Treatments

JQ1 was used as a model compound for BETi in this study. JQ1 was not investigated clinically due to its short half-time [33]. Advanced BETi as OTX015 and JQ35 were developed and already clinically investigated in B-cell lymphoma, multiple myeloma, castrate-resistant prostate cancer, midline carcinoma, and non-small-cell lung cancer, but to our knowledge, not in bladder cancer [27,36]. Therefore, we investigated the advanced BETi OTX015 and JQ35 in the triple combination (together with cisplatin) as replacement for JQ1 to enable a future potential clinical application perspective for the triple treatment in bladder cancer. The cell lines J82 and J82 cisR were selected for this investigation since the triple treatment was most effective in these cell lines. As additional approach, the dual HDAC- and BET-inhibitor **20** was investigated [51]. Inhibition of two target proteins simultaneously, HDAC and BRD proteins, in one compound could simplify therapy regimes, known in medicinal chemistry as dual-targeting approach. Compound **20** was described in the literature as a compound with sub-micromolar inhibitory activity against HDAC 1-3 and 6 [51]. The IC_50_ values at HDAC4 (207 µM) and HDAC8 (6.03 µM) support selectivity towards class I and IIb HDACs (Figure S15, Table S6). The inhibitory activity to BRDs was reported in the single-digit micromolar range [51]. As **20** can inhibit both epigenetic targets, **20** was only tested in dual combination with cisplatin in all four cancer cell lines.

Firstly, the IC_50_ values for all three compounds were determined in J82 and J82 cisR. Compound **20** was additionally tested in T24 and T24 LTT. Results are presented in Figure S16 and Table 4.

**Figure 10:**
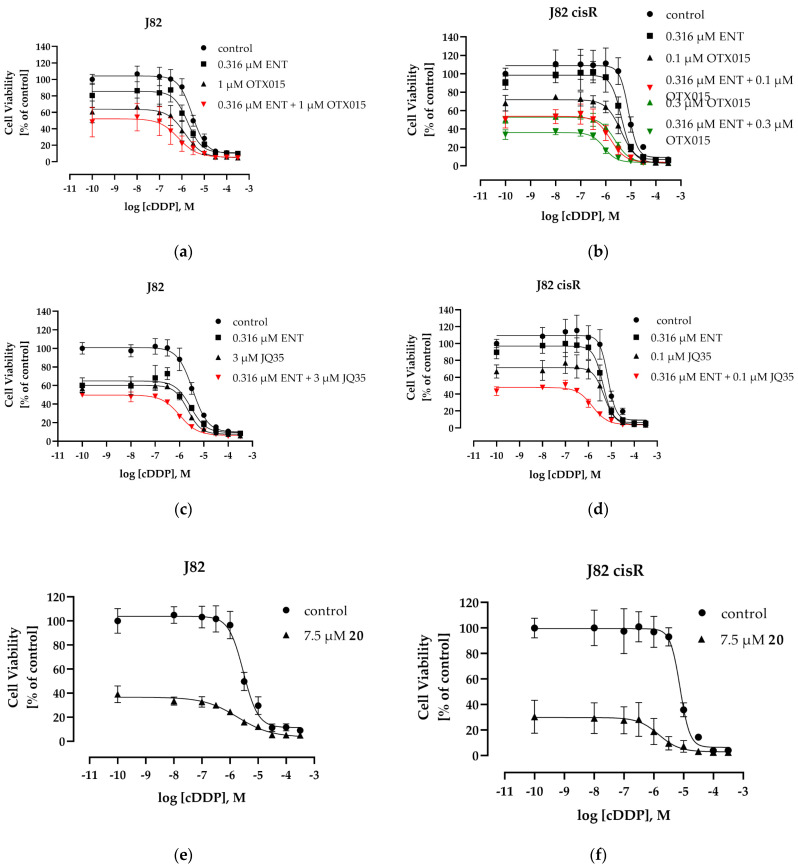
Concentration–effect curves of cisplatin (MTT assay) of (**a**,**c**,**e**) J82 and (**b**,**d**,**f**) J82 cisR. Cells were preincubated with entinostat and/or OTX015 or JQ35 or compound **20** alone for 48 h followed by addition of cisplatin for another 72 h. Control means concentration–effect curve of cisplatin in absence of epigenetic inhibitors. Data shown are the mean ± SD of at least one experiment, carried out in triplicates.

The IC_50_ values of OTX015 and JQ35 were comparable to the IC_50_ values of JQ1 in J82 and J82 cisR (Table 1, J82 18.8 µM, J82 cisR 2.22 µM), except for JQ35 in J82. JQ35 had a 3.7-fold-increased IC_50_ value in J82 cells compared to JQ1. Compound **20** was approximately equally cytotoxic in the single-digit micromolar range in all four cell lines. Then, the effect of entinostat plus OTX015 or entinostat plus JQ35 or, alternatively, **20** only on the IC_50_ of cisplatin was investigated. The concentration–effect curves of cisplatin are presented in Figure 10 and Figure S17. The IC_50_ values and shift factors of the combination treatments are shown in Table 5 (additional IC_50_ values and shift factors in Tables S7 and S8).

**Figure 11:**
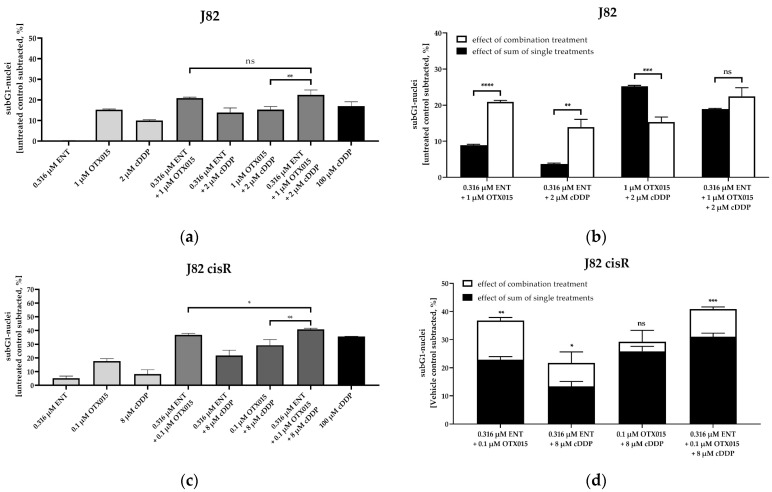
Induction of subG1-nuclei. Cells were preincubated with entinostat and OTX015 for 48 h followed by addition of cisplatin for another 24 h. Apoptotic cells were defined by subG1 fraction (flow cytometry). Data shown are the mean + SD of one representative experiment out of at least two, each carried out at least in triplicates. Untreated control was subtracted. (**a**) J82; (**c**) J82 cisR. (**b**,**d**) show the comparison between the sum of the single compound effects (black bars) versus the effect of the combination treatments (white bars) in J82 (**b**) and J82 cisR (**d**). Statistical analysis was performed using unpaired *t*-test. Level of significance: ns (*p* ≥ 0.05); * (*p* < 0.05); ** (*p* < 0.01); *** (*p* < 0.001); **** (*p* < 0.0001).

As previously observed for JQ1, OTX015 and JQ35 also revealed the largest SF in combination with entinostat (Table 5 and Table S7), leading to a complete reversal of cisplatin resistance in J82 cisR. The application of one epigenetic inhibitor only gave a slight reduction in the IC_50_ value of cisplatin (Table S7). Overall, the combination of the BETi, OTX015 or JQ35, and entinostat showed comparable sensitizing effects on the IC_50_ value of cisplatin, as JQ1 did. Compound **20** had an SF of 5.31 in J82 cisR, which is comparable to the effect of the combination of entinostat plus OTX015 or JQ35. In J82, T24, and T24 LTT, the SF of **20** was mostly only <2 (Table 5 and Table S8); however, 7.5 µM of **20** induced a significant decrease in cell viability (approx. 60–70%) in all bladder cancer cell lines. (Figure 10 and Figure S17). OTX015 was chosen for further detailed investigations due to its overall presenting the highest SF in the triple combinations in J82 and J82 cisR (Table 5). We investigated whether OTX015 alone and in combination treatments induced comparable effects as JQ1 regarding apoptosis induction. The results of subG1 induction are shown in Figure 11 and representative cytometry flow images are presented in Figure S18.

The triple combination of entinostat, OTX015, and cisplatin exhibited the largest subG1 fraction in J82 and J82 cisR. The triple combination induced significantly more subG1 nuclei than the single or dual combinations in J82 cisR. The same applied to J82, except for the combination of entinostat and OTX015. In J82, the difference between the sum of the single treatments and the triple combination treatment was not significant. Even the dual combination of entinostat and OTX015 was as effective as the triple treatment and showed a significant increase in subG1 nuclei in comparison to the sum of single treatments. In J82 cisR, the number of subG1 nuclei induced by triple treatment was significantly higher than the sum of the single treatments. Overall, OTX015 in combination with entinostat and cisplatin yielded comparable results to JQ1 in the induction of subG1 nuclei (Figure 4 and Figure 11).

**Figure 12:**
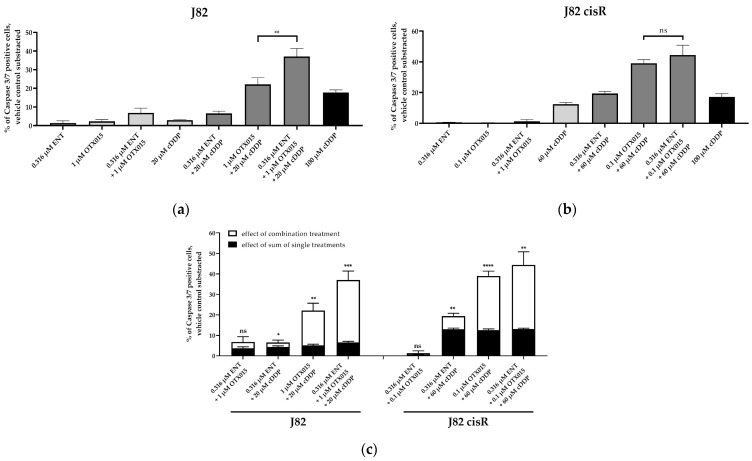
Caspase 3/7 activation in (**a**) J82 and (**b**) J82 cisR. Cells were preincubated with entinostat and OTX015 for 48 h followed by addition of cisplatin for another 24 h. (**c**) shows the comparison between the sum of the single compound effects (black bars) versus the effect of the combination treatments (white bars) in J82 and J82 cisR. Data shown are the mean + SD of one representative experiment out of at least two, each carried out in duplicates. Significance was calculated using unpaired *t*-test. Level of significance: ns (*p* ≥ 0.05); * (*p* < 0.05); ** (*p* < 0.01); *** (*p* < 0.001); **** (*p* < 0.0001).

**Figure 13:**
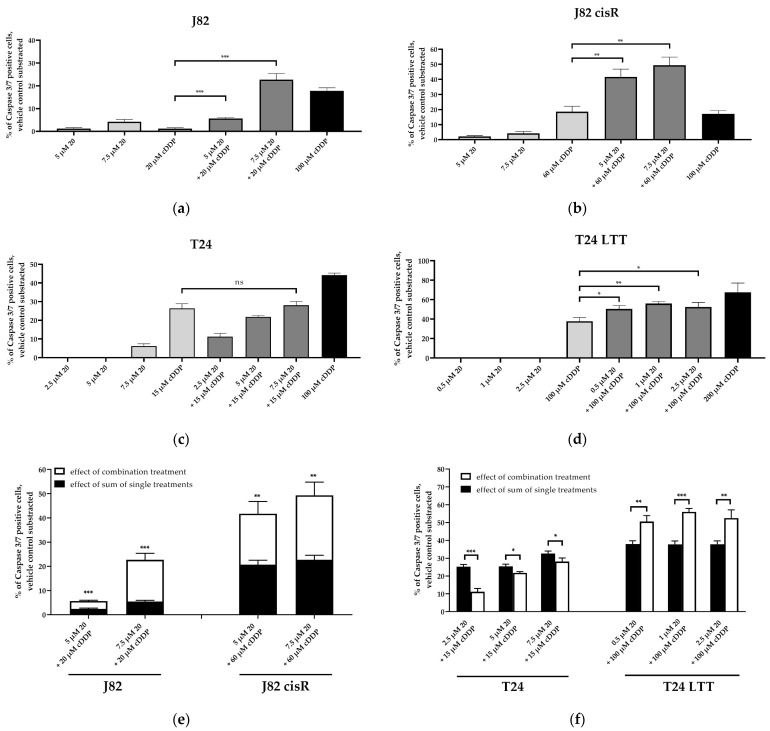
Caspase 3/7 activation in (**a**) J82, (**b**) J82 cisR, (**c**) T24, and (**d**) T24 LTT. Cells were preincubated with **20** for 48 h followed by addition of cisplatin for another 24 h (T24 LTT 48 h). (**e**,**f**) show the comparison between the sum of the single compound effects (black bars) versus the effect of the combination treatments (white bars) in J82 and J82 cisR (**e**) and T24 and T24 LTT (**f**). Data shown is the mean + SD of one representative experiment out of at least two, each carried out in duplicates. Significance was calculated using unpaired *t*-test. Level of significance: ns (*p* ≥ 0.05); * (*p* < 0.05); ** (*p* < 0.01); *** (*p* < 0.001).

Then, we investigated whether the induction of apoptosis was triggered by caspase 3/7 activation as observed for JQ1. The activation of caspase 3/7 was investigated using the using dual and triple combinations consisting of entinostat, OTX015, and cisplatin in J82 and J82 cisR (Figure 12). In addition, we investigated the dual combination of **20** and cisplatin in J82, J82 cisR, T24, and T24 LTT (Figure 13). Representative fluorescence images are presented in Figure S19.

The triple combination induced the highest caspase 3/7 activation followed by the combination of OTX015 and cisplatin in J82 and J82 cisR, although the difference was not significant in J82 cisR. The synergistic effect of the triple combination was evident as seen by a huge increase in caspase 3/7 activation in J82 and J82 cisR compared to the sum of the single treatments. These results again demonstrated that OTX015 behaved analogous to JQ1 in the triple combination (Figure 5 and Figure 12). Remarkably, the triple combination with OTX015 exceeded by far the caspase activation induced by 100 µM cisplatin, again demonstrating the potential of the triple combination for overcoming cisplatin resistance.

Compound **20** only weakly activated caspase 3/7 as a single treatment in all four cell lines, although **20** combines both features as HDACis and BETis (Figure 13). However, the dual combination of **20** and cisplatin revealed a significant increase in caspase 3/7 activation compared to cisplatin alone in J82 and J82 cisR, exceeding by far the effect of 100 µM cisplatin (Figure 13). The combination treatment also demonstrated a significantly higher caspase 3/7 activation in T24 LTT, although increasing concentrations of **20** did not further increase this effect. Remarkably, already 0.5 µM **20** in combination with cisplatin resulted in a higher caspase 3/7 activation in T24 LTT compared to cisplatin alone, although the IC_50_ of **20** in T24 LTT cells is much higher (4.66 µM, Table 4). In T24 cells, the combination of **20** and cisplatin did not increase caspase 3/7 activation compared to cisplatin alone up to 7.5 µM **20,** which is slightly above its IC_50_ value in T24 cells (6.95 µM, Table 4). In summary, our results confirm a synergistic effect of **20** and cisplatin for caspase 3/7 activation in J82, J82 cisR, and T24 LTT (Figure 13) and additionally confirm the higher potency of the combination of HDACi and BETi (including **20**) in cisplatin-resistant cell lines (see also Table 4).

**Figure 14:**
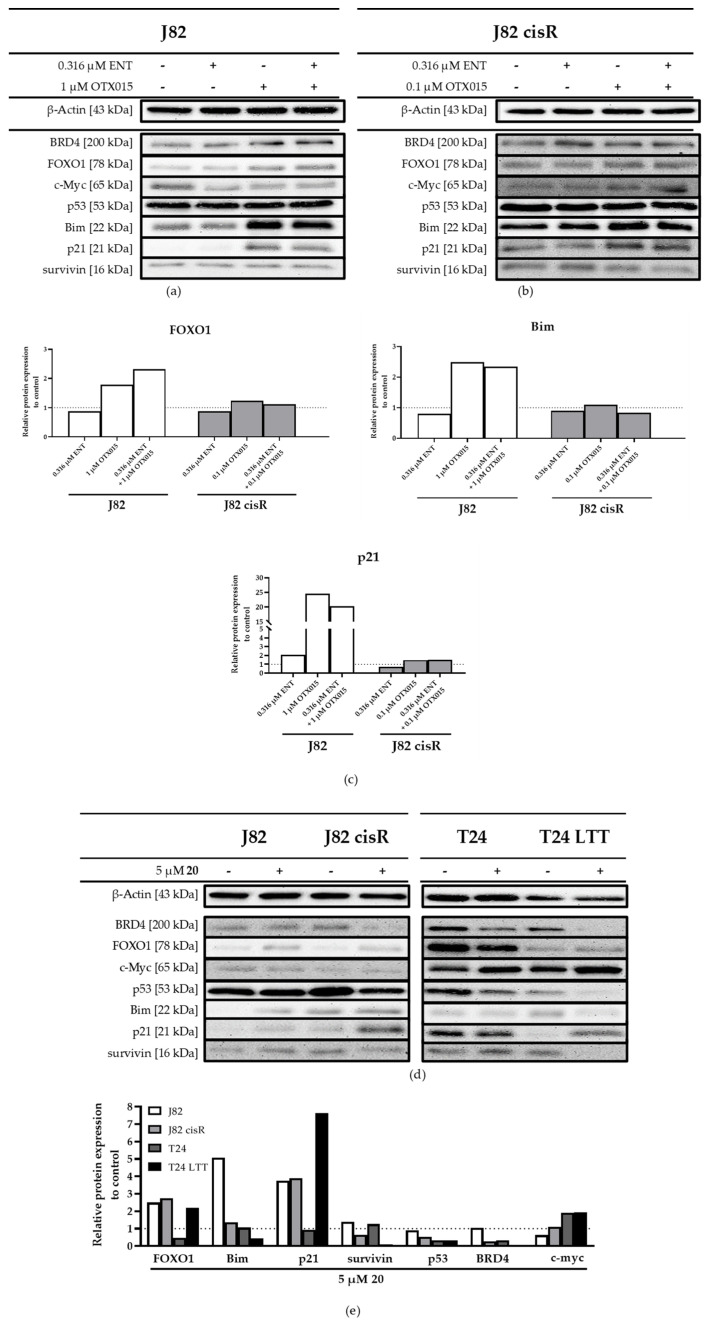
Western blot analysis of (**a**) J82, (**b**) J82 cisR and (**d**) J82, J82 cisR, T24, and T24 LTT. Cells were incubated with entinostat, OTX015, or **20** for 48 h. Data shown are a representative blot out of at least two different protein samples. Uncropped blots are presented in the Supplementary Materials (Figures S20–S23). Relative protein expression of selected proteins related to β-actin as loading control in (**c**) J82 and J82 cisR and (**e**) J82, J82 cisR, T24, and T24 LTT**.** Ratio of control was set to 1, represented by the dashed line. Western blots were analyzed with ImageJ. The remaining investigated proteins are displayed in Figure S24.

Similar to the analysis of protein expression changes upon entinostat and JQ1 treatment (Figure 9), we analyzed altered protein expression upon treatment with OTX015, entinostat, and their combination, as well as with **20** in Western blot (Figure 14).

In J82 and J82 cisR cells, expression of FOXO1 and p21 increased upon treatment with OTX015 and the combination of OTX015 plus entinostat but not with entinostat alone (Figure 14). The expression of Bim was clearly increased by OTX015 and the combination treatment in J82, but to a lesser extent in J82 cisR. A decrease in survivin expression was found for OTX015 and OTX015 plus entinostat in J82 cisR, whereas its expression was unchanged in J82. Furthermore, the expression of p53 remained unchanged for all treatments in J82 cisR and J82. Expression of c-Myc slightly increased upon treatment with OTX015 and the combination in agreement to the findings with JQ1 (Figure 9) in J82 cisR. However, in J82 cells, expression of c-Myc decreased upon treatment with OTX015 and the combination, which was different with the BETi JQ1 (Figure 9). BRD4 expression slightly increased upon entinostat treatment but not with OTX015 or the combination in J82 cisR. However, incubation with OTX015 and combination of entinostat and OTX015 led to an increase in BRD4 in J82 cells. In summary, changes in protein expression in J82 and J82 cisR cells were similar for entinostat plus OTX015 as observed for entinostat plus JQ1 (Figure 9 and Figure 14). Major changes in expression were found for FOXO1, p21, and Bim.

Upon treatment with compound **20**, similar expression changes were obtained as with the combination of entinostat and OTX015 (Figure 14). FOXO1 expression increased in J82, J82 cisR, and T24 LTT, but decreased in T24. P21 expression increased upon treatment with **20** in all cell lines except T24. Of interest, increase was most prominent in the cisplatin-resistant cell lines J82 cisR and T24 LTT. Bim expression increased in J82 only and was even decreased in T24 LTT. Survivin expression slightly increased in the cisplatin-sensitive cell lines J82 and T24 but decreased clearly in the cisplatin-resistant cell clones J82 cisR and T24 LTT. P53 was downregulated by **20** in all cell lines. The expression of c-Myc decreased in J82, remained unchanged in J82 cisR, and slightly increased in T24 and T24 LTT cells. Except for in J82, BRD4 was downregulated upon treatment with **20** in all cell lines. In summary, **20** increased the expression of FOXO1 and p21 and decreased expression of survivin, particularly in the cisplatin-resistant clones.

### 3.5. Selectivity of the Triple Combination Entinostat, BETi, and Cisplatin for Cancer over Non-Cancer Cells

A limiting factor in the clinical administration of anticancer drugs is their toxicity to non-cancer cells. Overall, the triple combination of entinostat, the BETi JQ1 or OTX015, and cisplatin delivered promising anticancer effects in two pairs of cisplatin-sensitive and -resistant bladder cancer cells. Here, we assessed the cytotoxicity of the triple treatments in non-cancer cell lines. The HBLAK cells were chosen as a non-cancerous entity from the bladder and HEK293 cells as a second sample for non-cancer cells. Concentration–effect curves of the compounds were performed in both cell lines. In HBLAK cells, JQ1 was used as the BETi, and in HEK293, JQ1 and OTX015 were used as the BETis (Figure S25, Table 6).

**Figure 15:**
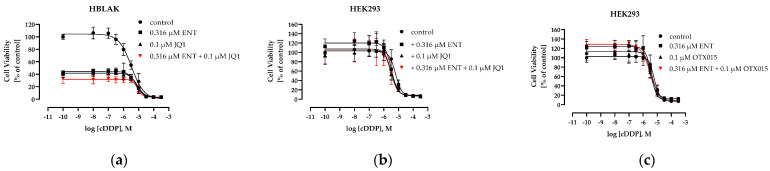
Concentration–effect curves of cisplatin (MTT assay) in (**a**) HBLAK and (**b**,**c**) HEK293. Cells were preincubated with entinostat and/or JQ1 or OTX015 for 48 h followed by addition of cisplatin for another 72 h. Control means concentration–effect curve of cisplatin in absence of epigenetic inhibitors. Data shown are mean ± SD of at least three independent experiments, each carried out in triplicates.

Both non-cancer cell lines had similar IC_50_ values for cisplatin as the cisplatin-sensitive cancer cell lines J82 or T24 (Table 1). Entinostat had a lower IC_50_ value in HBLAK than in HEK293. The IC_50_ value of entinostat in HBLAK was comparable to the IC_50_ in T24 LTT, and the IC_50_ in HEK293 was similar to the IC_50_ in T24 (Table 1). In both non-cancer cell lines, the IC_50_ values of JQ1 and OTX015 were similar to the IC_50_ values in J82 cisR or T24 cells. Thus, the IC_50_ values were generally in a similar range in cancer and non-cancer cells. Next, the effect of entinostat and JQ1 or OTX015 alone and in combination on the IC_50_ of cisplatin was investigated (Figure 15, Table 7 and Table 8). Concentrations used in non-cancer cell lines were as used for the cancer cell lines.

Overall, all combination treatments, including triple combinations, yielded low SFs in both non-cancer cell lines. SFs of around two were calculated for HEK293. Surprisingly, in HBLAK, the IC_50_ values of cisplatin even increased (SF < 1) under treatment with the epigenetic inhibitors. However, in HBLAK, treatment with entinostat and/or JQ1 in the absence of cisplatin reduced cell viability by 60–65% (Figure 15a), whereas in HEK293, cell viability was only reduced by up to 20% (Figure 15b,c). All SFs of the triple combination were <2.1 in non-cancer cells (Table 7 and Table 8) and, thus, much lower than the SFs of the cancer cell lines which were up to 15.6 (Table 2).

**Figure 16:**
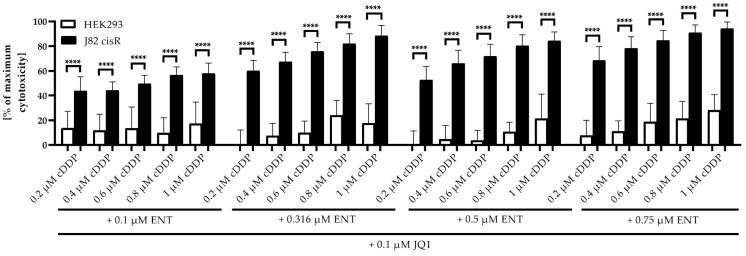
Cytotoxicity (MTT assay) of the triple combination entinostat, JQ1, and cisplatin in HEK293 cells (white bars) and J82 cisR cells (black bars). The cells were preincubated with entinostat and JQ1 for 48 h, followed by the addition of cisplatin for another 72 h. Shown are the mean + SD from at least three independent experiments, each carried out in triplicates. Data were normalized to untreated control, where 100% cell viability was set as 0% cytotoxicity. Significance was calculated using unpaired *t*-test. **** (*p* ≤ 0.0001).

Finally, the cytotoxic effect of various triple combinations (fixed concentration JQ1, 0.1–0.75 µM entinostat, 0.2–1.0 µM cisplatin) were investigated in HEK293 and J82 cisR cells for direct comparison of their toxicities (Figure 16).

Figure 16 shows that all triple combinations had a significantly higher cytotoxicity in the cisplatin-resistant cancer cell line J82 cisR than in the non-cancer cell line HEK293. Since the IC_50_ values of entinostat, JQ1, and cisplatin are approximately the same in cancer and non-cancer cell lines, these results suggest that non-cancer cell lines are much less susceptible to sensitization by epigenetic inhibitors, here HDACis and BETis, against cisplatin than cancer cells (Table 2, Table 7 and Table 8, Figure 16). Even sub-micromolar concentrations of entinostat, JQ1 and cisplatin were sufficient to achieve a high cytotoxic effect in cisplatin-resistant J82 cisR cells due to reversal of cisplatin resistance, whereas cytotoxicity was very low in HEK293 (Figure 16). Thus, our results indicate a strong selectivity of the triple combination for cancer over non-cancer cell line.

## 4. Discussion

The development of cisplatin resistance is an unresolved problem [71,72]. The use of combination therapies offers the potential to overcome intrinsic or acquired resistance of cisplatin through increased efficacy and selectivity. Especially, non-responders of established cisplatin-based chemotherapy or patients with cisplatin-resistant tumors need new therapeutic approaches, among them bladder cancer patients. The inhibition of epigenetic regulators such as HDACs or BET proteins has often been found to sensitize cancer cells to cisplatin, including bladder cancer cells [14,32,72,73]. In this study, we investigated the triple combination consisting of the class I HDACi entinostat; a BETi, such as JQ1 or OTX015; and cisplatin in two pairs of cisplatin-sensitive and -resistant bladder cancer cells. A previous study has demonstrated a synergistic effect of the class I HDACi romidepsin and JQ1 in bladder carcinoma cell lines [48]. However, this previous study did not investigate the effect of romidepsin and JQ1 on cisplatin. In the present study, we used the class I-selective HDACi entinostat but found only moderate mutual sensitizing effects of entinostat and JQ1 (SF < 2, Table S3), resulting in a study design including cisplatin as a further cytotoxic agent with clinical relevance in bladder carcinoma. The triple combination of entinostat, JQ1, and cisplatin in this study resulted in a strongly reduced IC_50_ value of cisplatin obtaining shift factors of >2 in all four bladder carcinoma cell lines. In accordance with previous results from our groups [74,75], 48 h preincubation of the epigenetic modulators prior to addition of cisplatin for another 72 h was much more effective (SF of cisplatin up to 15.6-fold, Table 2) than co-incubation of all three compounds (SF < 2, Table S4). The triple combination (applying pre-incubation of the epigenetic modulators) resulted in strong synergistic effects according to Chou-Talalay and Bliss analysis (Table 3, Figure 2 and Figure 3) in all four cell lines. This led to a complete reversal of cisplatin resistance in J82 cisR cells (IC_50_ value reduced from 8.49 to 0.54 µM, Table 2) and an even lower IC_50_ value of cisplatin than in the native cell line J82 (2.16 µM), which can be designated as “oversensitization”. Even the highly resistant T24 LTT cell line (IC_50_ value cisplatin 75.0 µM) was strongly sensitized against cisplatin by a factor of 4.3 applying the epigenetic modulators, although no complete resistance reversal was achieved

Studies investigating dual combinations consisting of an HDACi plus cisplatin or an HDACi plus a BETi found an enhanced induction of apoptosis through increased caspase 3/7 activation [14,48]. These results are consistent with our findings that the synergistic effect of the triple treatment is based on synergistic increase in caspase 3/7 activation and increased apoptosis (Figure 4 and Figure 5). Among other proteins, JQ1 inhibits BRD4, which is a main regulator for numerous genes of the DNA repair system [76]. Therefore, it appears reasonable that inhibition of BRD4 by JQ1 or OTX015 may contribute to a higher number of double-strand breaks. Indeed, the triple treatment including JQ1 resulted in significantly more double-strand breaks than the dual combination comprising entinostat and cisplatin (Figure 6b), underlining the synergistic effect of the triple combination.

The underlying mechanism for the synergistic effect may at least in part be attributed to increased expression of FOXO1, Bim and p21 in all four cell lines as analyzed by qPCR and Western blot. As the literature confirms, these proteins are increased by class I HDACi entinostat or BETi JQ1 and play a key role for DNA damage-induced cell death. [19,54,70,77]. Further, the literature reports that JQ1-mediated BRD4 inhibition leads to downregulation of mutant p53, resulting in upregulation of p21 in triple-negative breast cancer cells [78]. Both native bladder cell lines used in this study have mutated p53 (J82 missense mutation, T24 non-sense mutation) [79,80,81,82]. p53 mutations can cause chemoresistance via different mechanisms, one of which is partially preventing or impeding drug-induced apoptosis. Additionally, mutated p53 can prevent cells from initiating G1 or G2/M arrest, likely due to inhibition of p21 expression [83]. Our Western blot results are consistent with literature reports on expression of mutant p53, as p53 was downregulated in all four cell lines by the dual combination of entinostat and JQ1, whereby p21 was upregulated (Figure 9 and Figure S7). Particularly in J82 cells, p21 expression was strongly enhanced by treatment with JQ1 with or without entinostat. High levels of p21 can then lead to increased cell cycle arrest and apoptosis induction [84]. It was shown in bladder carcinoma cells that overexpression of c-Myc and the resulting increase in MMS19 expression facilitate cell proliferation and the development of cisplatin resistance [85]. Many studies revealed a downregulation of c-Myc by JQ1 [70,86]. Surprisingly, we could observe a downregulation of c-Myc only in T24 and T24 LTT cells, whereas J82 and J82 cisR exhibited the opposite tendency (Figure 9). It can therefore be concluded that the suppression of c-Myc and its downstream signaling in bladder carcinoma cell lines can only partly explain the synergistic effect of entinostat and JQ1 and cisplatin. Further studies are needed to fully understand the mechanisms and network of protein interactions leading to the synergistic effect of the triple combination observed in our study. As common changes in gene expression upon treatment with entinostat and JQ1 in the four investigated bladder cancer cell lines, we have discovered an upregulation of p21, FOXO1, and Bim, all of which will at least in part contribute to the observed synergistic effect with cisplatin.

**Figure 17:**
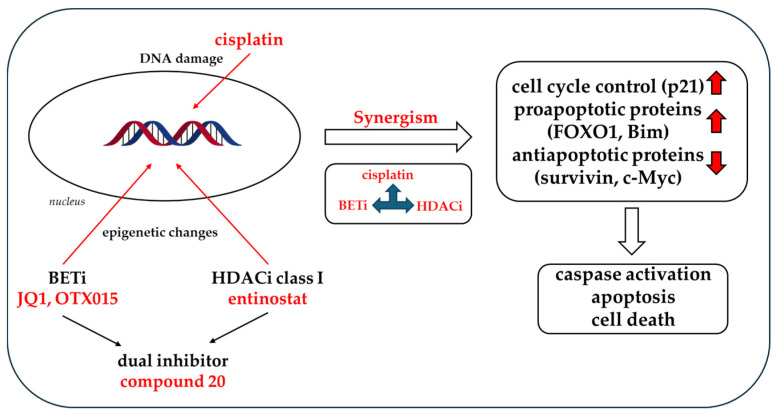
Overview of the effects of the combination of class I HDACi, BETi JQ1, or OTX015 or the dual inhibitor **20** and cisplatin.

OTX015, already investigated in clinical trials, demonstrated comparable effects as JQ1. Oral administration and longer half-life are key advantages of OTX015 over JQ1 [26,27,39]. In the triple-combination experiment using entinostat, OTX015, and cisplatin, we observed complete resensitization against cisplatin in the J82 cisR cells as we did with JQ1. The IC_50_ value of cisplatin was reduced from 7.80 to 0.85 µM, corresponding to an SF of 9.2 (Table 5). Further, the triple treatment of entinostat, OTX015, and cisplatin overall confirmed results obtained with JQ1 concerning caspase 3/7-mediated apoptosis (Figure 11 and Figure 12) and protein expression changes: reduced p53, increased p21, and increased FOXO1 expression in J82 and J82 cisR cells, whereas Bim expression was only increased in J82 (Figure 14a–c). Taken together, JQ1 can be equivalently exchanged by OTX015. Figure 17 summarizes the effects of the triple combination consisting of entinostat, BETi JQ1, or OTX015 or the dual inhibitor **20** and cisplatin on caspase-mediated apoptotic cell death.

In conclusion, the triple combination of entinostat, a BETi, preferably OTX015, and cisplatin yields a highly synergistic caspase-mediated apoptosis and cytotoxicity, completely reversing cisplatin resistance and even leading to oversensitization, meaning to increase cisplatin potency even beyond the potency in the native (non-resistant) cell lines. Thus, this combination is highly promising to be further explored in bladder carcinoma models and eventually in clinical trials, particularly since all components of this combination have already undergone clinical evaluation.

As an additional feature, we have characterized compound **20**, a dual HDACi and BETi previously described [51]. A newer approach in medicinal chemistry follows up on the combination of two or more inhibitory principles in one molecule (multi-target inhibitors) if synergistic effects are likely to simplify the treatment [87,88]. Several dual HDAC and BET inhibitors have already been investigated in various cancer entities, including bladder cancer [51,89,90,91,92,93]. Here, we characterized the dual HDAC and BET inhibitor **20** in combination with cisplatin in the two cisplatin-sensitive and -resistant bladder cancer cell pairs. Compound **20** is a combination of a 4-acyl pyrrole moiety derived from XD14 (BRD4(1) inhibitor) and the zinc-binding group related to panobinostat (HDACi) [51,94]. The combination of **20** and cisplatin led to a strong decrease in the IC_50_ value of cisplatin from 7.80 µM to 1.47 µM (SF 5.31) and thus to a complete cisplatin resensitization of J82 cisR cells (Figure 10). Consequently, the combination of **20** and cisplatin was as effective as the triple combination in J82 cisR cells. In addition, a significant increase in caspase 3/7 activation was observed upon combination of **20** and cisplatin in J82, J82 cisR, and T24 LTT but not T24 cells (Figure 13). This was accompanied by increased expression of FOXO1 and p21 in all cancer cell lines except T24, which may explain the lack of increase in caspase 3/7 activation in T24 by the combination of **20** and cisplatin. Notably, survivin expression was decreased in both cisplatin-resistant cell lines (J82 cisR, T24 LTT, Figure 14d,e). Taken together, the dual inhibitor **20** demonstrated similar changes in protein expression as the combination of entinostat and JQ1 or OTX015. In conclusion, the combination of the dual HDAC and BET inhibitor, **20,** plus cisplatin yielded comparable efficacy as the triple combination of entinostat, JQ1 or OTX015, and cisplatin, thus confirming the efficacy of the dual targeting approach and simplifying treatment regimes. Further studies are needed to investigate the pharmacokinetics, toxicity, and long-term efficacy of compound **20**, including other cancer models.

Ideally, the highly synergistic effect of the triple combination is selective for the cancer cells and does not, or does to a much lesser extent, affect non-cancer cells. The single compounds have similar toxicity in the bladder cancer cell lines and two non-cancer cell lines, the bladder-tissue-derived cell line HBLAK and the kidney cell line HEK293 (Table 1 and Table 6). However, in combination with cisplatin, the toxicity is much lower in the non-cancer than in the bladder cancer cells, thus suggesting selectivity for the bladder cancer cells. The reduction in the IC_50_ of cisplatin upon pretreatment with entinostat and JQ1 or OTX015 was small in HEK293 cells, and the SFs were even <1 in HBLAK cells (Figure 15). A selectivity for cancer over non-cancer cells is crucial for combination treatments aiming to overcome cisplatin resistance in patients. The selectivity of the triple combination for cancer over non-cancer cells is impressively shown for J82 cisR and HEK293 cells in Figure 16 using different concentrations of JQ1, entinostat, and cisplatin relevant for in vivo studies [27,60,95].

## 5. Conclusions

Our study showed that the triple combination of the HDACi entinostat, a BETi, preferably OTX015, and cisplatin or a dual-acting HDAC/BET inhibitor plus cisplatin is highly synergistic in bladder carcinoma cell lines and completely reverses cisplatin resistance. The synergistic cytotoxicity is due to an enhanced caspase-mediated induction of apoptosis caused by increased expression of p21, FOXO1, and Bim. The triple combination is selective for bladder cancer compared to the non-cancer cell lines HBLAK and HEK293. Thus, this study encourages further preclinical and eventually clinical trials with HDAC and BET inhibitors in combination with cisplatin to overcome cisplatin resistance and foster treatment efficacy in bladder cancer.

## Figures and Tables

**Table 1:** IC_50_ values (MTT, 72 h) of cisplatin, entinostat, and JQ1 in all bladder cancer cell lines. Data shown are the mean ± SD of at least three independent experiments, each carried out in triplicates.

	Cisplatin IC_50_ ± SD [µM]	Entinostat IC_50_ ± SD [µM]	JQ1 IC_50_ ± SD [µM]
**J82**	1.87 ± 0.06	10.4 ± 0.89	18.8 ± 1.55
**J82 cisR**	7.16 ± 0.24	7.60 ± 0.59	2.22 ± 0.41
**T24**	2.99 ± 0.12	5.68 ± 0.51	2.29 ± 0.42
**T24 LTT**	72.2 ± 3.57	1.38 ± 0.10	0.60 ± 0.08

**Table 2:** IC_50_ values (MTT) of cisplatin. Cells were preincubated with entinostat (ENT) and/or JQ1 for 48 h followed by addition of cisplatin for another 72 h. Control was untreated for 48 h followed by addition of cisplatin for another 72 h. Data shown are the mean ± SD of at least three independent experiments, each carried out in triplicates. All IC_50_ values of the combination treatments were significantly lower than the IC_50_ values of the cisplatin alone (control, at least *p* < 0.001).

Cell Line	Control	ENT Plus Cisplatin			JQ1 Plus Cisplatin			ENT Plus JQ1 Plus Cisplatin		
	Cisplatin IC_50_ ± SD [µM]	ENT [µM]	Cisplatin IC_50_ ± SD [µM]	SF	JQ1 [µM]	Cisplatin IC_50_ ± SD [µM]	SF	ENT+ JQ1 [µM]	Cisplatin IC_50_ ± SD [µM]	SF
**J82**	2.16 ± 0.14	0.316	1.63 ± 0.24	1.33	1	0.77 ± 0.13	2.81	0.316 + 1	0.38 ± 0.08	5.62
**J82 cisR**	8.49 ± 0.51	0.316	4.62 ± 0.57	1.84	0.1	2.96 ± 0.38	2.87	0.316 + 0.1	1.53 ± 0.22	5.54
					0.3	1.35 ± 0.15	6.27	0.316 + 0.3	0.54 ± 0.06	15.6
**T24**	4.15 ± 0.12	0.316	3.08 ± 0.15	1.35	0.3	0.84 ± 0.09	4.92	0.316 + 0.3	1.18 ± 0.13	3.52
**T24 LTT**	75.0 ± 2.85	0.316	33.2 ± 3.51	2.26	0.075	67.0 ± 9.03	1.12	0.316 + 0.075	26.4 ± 4.28	2.85
					0.15	30.6 ± 2.32	2.45	0.316 + 0.15	17.5 ± 1.13	4.29

**Table 3:** The CI values of the triple combination studies (48 h preincubation with entinostat and JQ1, followed by addition of cisplatin for another 72 h). The CI values were calculated using compuSyn from the mean of at least three independent experiments, each carried out in triplicates. A CI < 1.0 indicate a synergistic effect (green), CI = 1 indicate an additive effect, and CI > 1 indicate an antagonistic effect (red); * means fraction affected was less than 0.20 [57].

**J82**									
		**0.316 µM ENT** **+ JQ1 [µM]**				**1 µM JQ1** **+ ENT [µM]**			
		**0.5**	**1**	**2**	**3**	**0.1**	**0.316**	**0.5**	**0.75**
**cisplatin [µM]**	0.05	*	0.502	0.413	0.311	*	0.341	0.463	0.216
	0.1	*	0.500	0.327	0.284	*	0.479	0.352	0.193
	0.15	1.40	0.370	0.247	0.215	1.12	0.401	0.269	0.170
	0.2	1.10	0.290	0.190	0.179	1.21	0.287	0.217	0.119
	0.3	0.662	0.163	0.131	0.124	0.786	0.196	0.141	0.087
**J82 cisR**									
		**0.316 µM ENT** **+ JQ1 [µM]**				**0.1 µM JQ1** **+ ENT [µM]**			
		**0.05**	**0.1**	**0.2**	**0.3**	**0.1**	**0.316**	**0.5**	**0.75**
**cisplatin [µM]**	0.2	1.10	0.374	0.223	0.100	1.34	0.644	0.480	0.285
	0.4	0.874	0.318	0.141	0.067	1.38	0.481	0.287	0.198
	0.6	0.782	0.243	0.118	0.067	1.07	0.352	0.249	0.167
	0.8	0.689	0.213	0.106	0.064	0.789	0.296	0.194	0.139
	1	0.538	0.192	0.104	0.063	0.795	0.250	0.183	0.133
**T24**									
		**0.316 µM ENT** **+ JQ1 [µM]**				**0.3 µM JQ1** **+ ENT [µM]**			
		**0.1**	**0.3**	**0.5**	**0.7**	**0.1**		**0.5**	**0.75**
**cisplatin [µM]**	0.2	*	0.949	0.438	0.349	1.32		0.643	0.509
	0.4	*	0.655	0.313	0.290	0.844		0.626	0.421
	0.6	*	0.491	0.294	0.265	0.726		0.431	0.392
	0.8	*	0.463	0.258	0.247	0.552		0.392	0.322
	1	*	0.360	0.243	0.235	0.484		0.398	0.287
**T24 LTT**									
		**0.316 µM ENT** **+ JQ1 [µM]**				**0.075 µM JQ1** **+ ENT [µM]**			
		**0.05**	**0.075**	**0.15**	**0.3**	**0.1**		**0.5**	**0.75**
**cisplatin [µM]**	5	*	*	*	1.23	*		*	1.48
	10	*	*	2.24	0.912	*		1.97	1.08
	15	*	*	1.04	0.690	*		0.940	0.698
	20	1.26	0.980	0.668	0.610	*		0.597	0.504
	25	0.865	0.684	0.522	0.457	*		0.466	0.390

**Table 4:** IC_50_ (MTT, 72 h) of OTX015, JQ35, and **20** in all bladder cancer cell lines. Data shown are the mean ± SD of at least three independent experiments, each carried out in triplicates. ND means not determined.

	OTX015 IC_50_ ± SD [µM]	JQ35 IC_50_ ± SD [µM]	20 IC_50_ ± SD [µM]
**J82**	18.9 ± 2.82	69.5 ± 7.88	7.17 ± 0.57
**J82 cisR**	1.04 ± 0.13	1.49 ± 0.34	7.00 ± 0.49
**T24**	ND	ND	6.95 ± 0.54
**T24 LTT**	ND	ND	4.66 ± 0.41

**Table 5:** IC_50_ values (MTT) of cisplatin. Cells were preincubated with entinostat and/or OTX015 or JQ35 or compound **20** alone for 48 h followed by addition of cisplatin for another 72 h. Control was untreated for 48 h followed by addition of cisplatin for another 72 h. Data shown are the mean ± SD of at least one experiment, carried out in triplicates. All IC_50_ values of the combination treatments were significantly lower than the IC_50_ values of cisplatin alone (control, *p* < 0.0001).

Cell Line	Control	+ 0.316 µM Entinostat								
	Cisplatin IC_50_ ± SD [µM]	OTX015 [µM]	Cisplatin IC_50_ ± SD [µM]	SF	JQ35 [µM]	Cisplatin IC_50_ ± SD [µM]	SF	20 [µM]	Cisplatin IC_50_ ± SD [µM]	SF
**J82**	3.02 ± 0.10	1	0.70 ± 0.23	4.31	3	1.01 ± 0.10	2.99	7.5	1.86 ± 0.56	1.62
**J82 cisR**	7.80 ± 0.23	0.1	1.55 ± 0.20	5.03	0.1	1.47 ± 0.13	5.31	7.5	1.47 ± 0.76	5.31
		0.3	0.85 ± 0.06	9.18						

**Table 6:** IC_50_ values (MTT, 72 h) of cisplatin, entinostat, JQ1, and OTX015 in non-cancer cell lines HBLAK and HEK293. Data shown are the mean ± SD of at least three independent experiments, each carried out in triplicates; ND = not determined.

	Cisplatin IC_50_ ± SD [µM]	Entinostat IC_50_ ± SD [µM]	JQ1 IC_50_ ± SD [µM]	OTX015 IC_50_ ± SD [µM]
**HBLAK**	1.71 ± 0.09	1.45 ± 0.18	2.37 ± 0.41	ND
**HEK293**	3.36 ± 0.09	4.47 ± 0.77	1.41 ± 0.26	1.98 ± 0.42

**Table 7:** IC_50_ values (MTT) of cisplatin. Cells were preincubated with entinostat and/or JQ1 for 48 h followed by addition of cisplatin for another 72 h. Data shown are mean ± SD of at least three independent experiments, each carried out in triplicates.

Cell line	Control	+0.316 µM ENT		+0.1 µM JQ1		+0.316 µM ENT +0.1 µM JQ1	
	Cisplatin IC_50_ ± SD [µM]	Cisplatin IC_50_ ± SD [µM]	SF	Cisplatin IC_50_ ± SD [µM]	SF	Cisplatin IC_50_ ± SD [µM]	SF
HBLAK	3.38 ± 0.18	6.89 ± 0.81	0.49	8.33 ± 0.76	0.41	8.45 ± 0.77	0.40
HEK293	6.26 ± 0.21	3.29 ± 0.30	1.90	3.19 ± 0.32	1.96	3.19 ± 0.45	1.96

**Table 8:** IC_50_ values (MTT) of cisplatin. Cells were preincubated with entinostat and/or OTX015 for 48 h followed by addition of cisplatin for another 72 h. Data shown are mean ± SD of at least three independent experiments, each carried out in triplicates.

Cell Line	Control	+0.316 µM ENT		+0.1 µM OTX015		+0.316 µM ENT +0.1 µM OTX015	
	Cisplatin IC_50_ ± SD [µM]	Cisplatin IC_50_ ± SD [µM]	SF	Cisplatin IC_50_ ± SD [µM]	SF	Cisplatin IC_50_ ± SD [µM]	SF
HEK293	6.26 ± 0.21	3.84 ± 0.31	1.63	3.74 ± 0.31	1.67	3.16 ± 0.24	1.98

## Data Availability

The data presented in this study are available in this article and the corresponding supplemental information.

## Supplementary Materials

The following supporting information can be downloaded at: https://www.mdpi.com/article/10.3390/cancers16193374/s1, Figure S1. Concentration–effect curves determined with a 72 h MTT assay in J82 (a) cisplatin (cDDP), (b) entinostat (ENT), (c) JQ1; J82 cisR (d) cisplatin, (e) entinostat, (f) JQ1; T24 (g) cisplatin, (h) entinostat, (i) JQ1; T24 LTT (j) cisplatin, (k) entinostat, (l) JQ1. Figure S2. Concentration–effect curves of entinostat or JQ1 (MTT assay). Figure S3. Concentration–effect curves of cisplatin (MTT assay). Figure S4. Synergistic cytotoxic effect of combination treatment in J82 (a)-(d), J82 cisR (e)-(h), T24 (i)-(l) and T24 LTT (m)-(p). Figure S5. Representative cytometry flow images are shown for control, entinostat, JQ1, cisplatin and their combination treatments in J82, J82 cisR, T24 and T24 LTT. Figure S6. Representative fluorescent imaging pictures (Thermofisher Arrayscan XTI) are shown for vehicle control, entinostat, JQ1, cisplatin and their combination treatments in J82, J82 cisR, T24 and T24 LTT. Figure S7. The relative protein expression of proteins of interest related to β-actin as loading control is shown in (a) J82, (b) J82 cisR, (c) T24 and (d) T24 LTT; Figure S8. Uncropped western blot, J82: blot at left top shows FOXO1 (a), β-Actin (b) and p21 (c) and the blot below illustrates p53 (d) and survivin (e), the blot on the right-side displays Bim (f), 48 h incubation. Figure S9. Uncropped western blot, J82: blot at right top shows BRD4 (a), c-Myc (b) and β-Actin (c), 48 h incubation. Figure S10. Uncropped western blot, J82 cisR: blot at left top shows FOXO1 (a), β-Actin (b) and p21 (c), the blot below illustrates p53 (d) and survivin (e), 48 h incubation. Figure S11. Uncropped western blot, J82 cisR: blot at right top shows at the bottom Bim (a), 48 h incubation. Figure S12. Uncropped western blot, J82 cisR: blot at top shows BRD (a), c-Myc (b) and β-Actin (c), 48 h incubation. Figure S13. Uncropped western blot, T24: blot at left top shows FOXO1 (a), β-Actin (b) and p21 (c), the blot below illustrates p53 (d) and survivin (e), the blot on the right-side displays BRD4 (f), c-Myc (g) and Bim (h), 48 h incubation. Figure S14. Uncropped western blot, T24 LTT: blot at left top shows FOXO1 (a), β-Actin (b) and p21 (c), the blot below illustrates p53 (d) and survivin (e), the blot on the right-side displays BRD4 (f), c-Myc (g) and Bim (h), 48 h incubation. Figure S15. HDAC enzyme inhibitory activity of 20, vorinostat and Panobinostat; Figure S16. Concentration–effect curves (MTT assay, 72 h) in J82 (a) OTX015, (b) JQ35, (c) 20; in J82 cisR (d) OTX015, (e) JQ35, (f) 20; in T24 (g) 20 and in T24 LTT (h) 20. Figure S17. Concentration–effect curves of cisplatin (MTT assay) of (a) J82; (b) T24; (c) T24 LTT. Figure S18. Representative cytometry flow images are shown for control, entinostat, OTX015, cisplatin and their combination treatments in J82 and J82 cisR. Figure S19. (a) Representative fluorescent imaging pictures (Thermofisher Arrayscan XTI) are shown for vehicle control, entinostat, OTX015, cisplatin and their combination treatments in J82, J82 cisR. (b) Representative fluorescent imaging pictures (Thermofisher Arrayscan XTI) are shown for vehicle control, 20, cisplatin and their combination treatments in J82, J82 cisR, T24 and T24 LTT. Figure S20. Uncropped western blot, J82, blot at left top shows FOXO1 (a), β-Actin (b) and p21 (c), the blot below illustrates p53 (d) and survivin (e), the blot on the right-side displays BRD4 (f), c-Myc (g) and Bim (h), 48 h incubation. Figure S21. Uncropped western blot, J82 cisR, blot at left top shows FOXO1 (a), β-Actin (b) and p21 (c), the blot below illustrates p53 (d) and Bim (e), 48 h incubation. Figure S22. Uncropped western blot, J82 cisR, the blot on the right-side displays BRD4 (a), c-Myc (b), β-Actin (c) and survivin (d), 48 h incubation. Figure S23. Uncropped western blot, J82, J82 cisR, T24 and T24 LTT, blot at left top shows FOXO1 (a), β-Actin (b) and p21 (c), the blot below illustrates p53 (d) and survivin (e), the blot on the right side displays BRD4 (f), c-Myc (g) and Bim (h), 48 h incubation. Figure S24. The relative protein expression of proteins of interest related to β-actin as loading control is shown in (a) J82, (b) J82 cisR. Figure S25. Concentration–effect curves determined by 72 h MTT assay in (a)-(c) HBLAK and (d)-(g) HEK293. Table S1. Overview of primary antibodies used in Western blot; Table S2. Primer sequences for RT-PCR. Table S3. IC50 values (MTT) of entinostat or JQ1. Table S4. IC50 values (MTT) of cisplatin. Table S5. CI values of the dual combination studies (48 h preincubation with entinostat or JQ1, followed by addition of cisplatin for another 72 h. Table S6. HDAC enzyme inhibitory activity of 20, vorinostat and Panobinostat. Table S7. IC50 values (MTT) of cisplatin; Table S8. IC50 values (MTT) of cisplatin.
