# Supplementary material for: Triple Combination of Entinostat, a Bromodomain Inhibitor, and Cisplatin Is a Promising Treatment Option for Bladder Cancer
Source: Cancers (Basel). 2024 Oct 2;16(19):3374. doi: 10.3390/cancers16193374 (PMC11476342; doi:10.3390/cancers16193374)
Supplement: Supplementary file 1 [file cancers-16-03374-s001.zip › cancers-3210083-supplementary.pdf]

Triple Combination of Entinostat, a Bromodomain Inhibitor, and Cisplatin Is a Promising Treatment Option for Bladder Cancer

Supplemental Information

Table S1. Overview of primary antibodies used in western blot.

Protein of interest	expected size (kDa)	Primary antibody (company, Catalog nr.)
BRD4	200	Cell Signaling Technologie; E2A7X
FOXO1	78	Cell Signaling Technologie; C29H4
c-Myc	65	Cell Signaling Technologie; D84C12
p53	53	Santa Cruz; sc-126
β-Actin	43	Santa Cruz; sc47778
Bim	22	bio-techne; NBP1-76963
p21	21	Cell Signaling Technology; #2947
survivin	16	bio-techne; AF886

Table S2. Primer sequences for RT-PCR.

Gene	Primer Forward	Primer Reverse
survivin	TGAGAACGAGCCAGACTTGG	TGTTCCTCTATGGGGTCGTCA
FOXO1	ATGTGTTGCCCAACCAAAGC	AGTGTAACCTGCTCACTAACCC
FOXO3a	AAGGATCACTGAGGAAGGG	GTGTCAGTTTGAGGGTCTGC
GUSB	ACCTCCAAGTATCCCAAGGGT	GTCTTGCTCCACGCTGGT
HPRT1	CCTGGCGTCGTGATTAGTGA	CGAGCAAGACGTTCAGTCCT
TBP	GTGACCCAGCATCACTGTTTC	GAGCATCTCCAGCACACTCT
p53	AGTCAGATCCTAGCGTCG	TCAGGAAGTAGTTTCCATAGG
Bim	AGACAGAGCCACAAGCTTCC	CAATACGCCGCAACTCTTGG
p21	TGCCGAAGTCAGTTCCTTGT	GTTCTGACATGGCGCCTCC

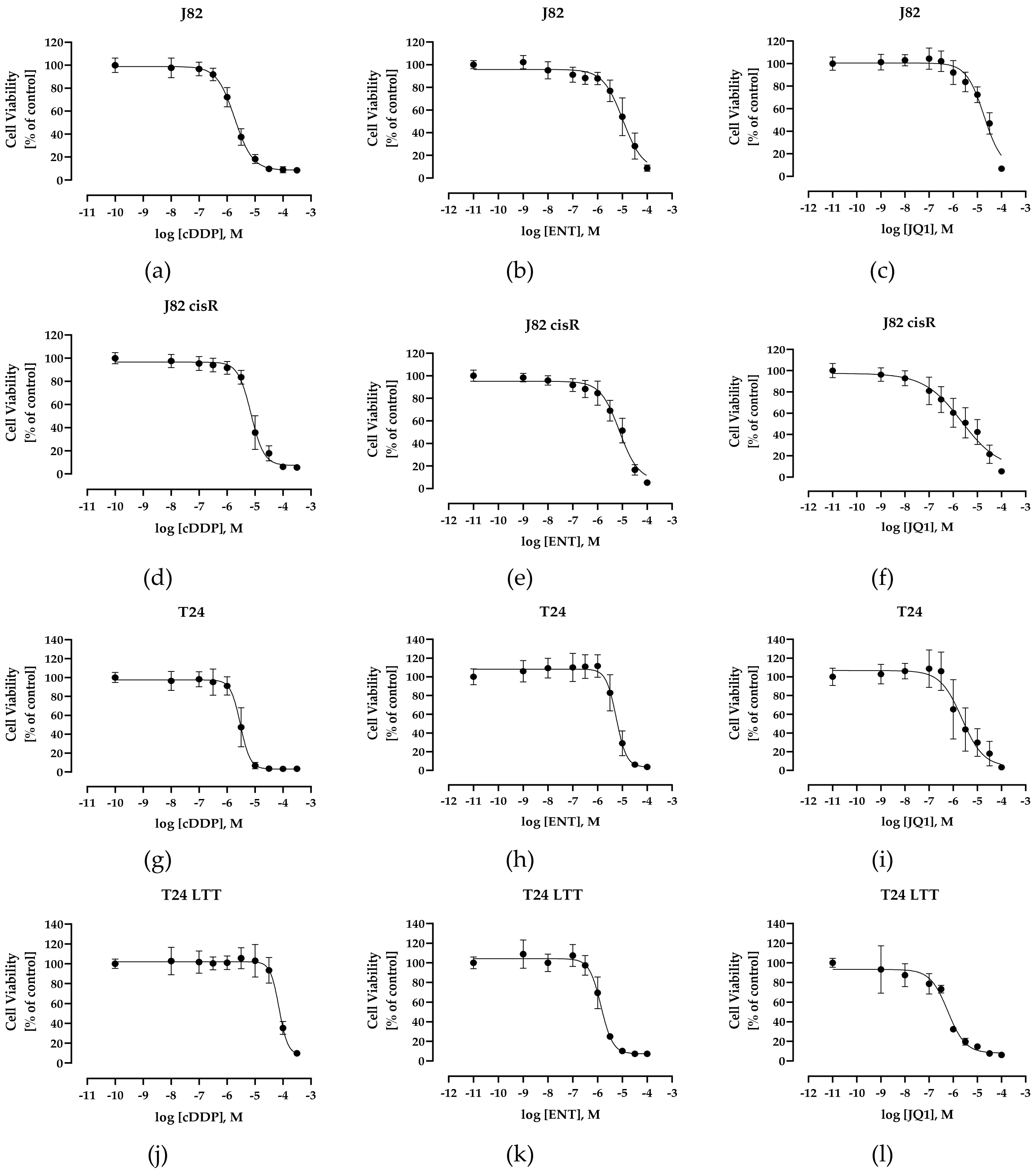

Figure S1. Concentration-effect-curves determined with a 72 h MTT-Assay in J82 **(a)** cisplatin (cDDP), **(b)** entinostat (ENT), **(c)** JQ1; J82 cisR **(d)** cisplatin, **(e)** entinostat, **(f)** JQ1; T24 **(g)** cisplatin, **(h)** entinostat, **(i)** JQ1; T24 LTT **(j)** cisplatin, **(k)** entinostat, **(l)** JQ1. Data shown are the mean ± SD of at least three independent experiments, each carried out in triplicates.

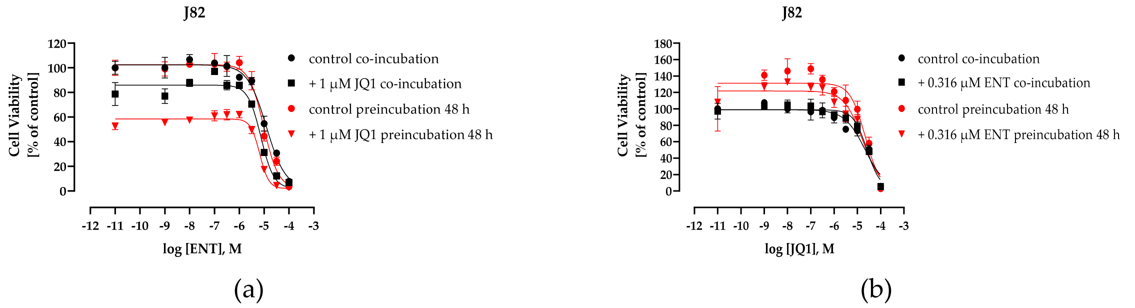

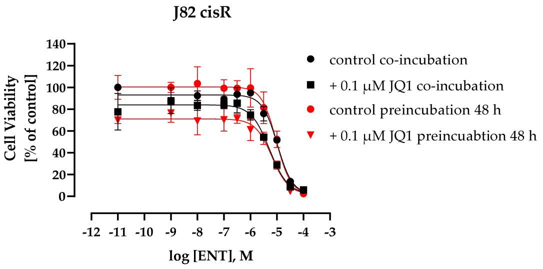

(c)

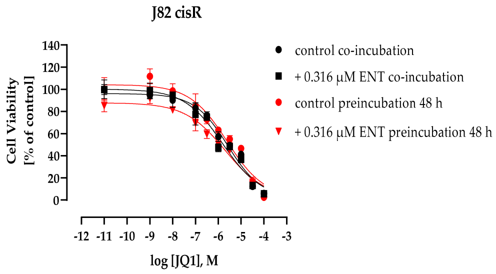

(d)

Figure S2. Concentration-effect-curves of entinostat or JQ1 (MTT-Assay). Cells were either preincubated with entinostat or JQ1 for 48 h followed by the addition of the second epigenetic inhibitor (JQ1 or entinostat) for another 72 h or co-incubated with both epigenetic inhibitors for 72 h. **(a)-(b)** J82; **(c)-(d)** J82 cisR. Control means concentration-effect-curve of entinostat or JQ1 in the absence of the second epigenetic inhibitor. Data shown are the mean ± SD of at least one representative experiment, carried out in triplicates.

Table S3. IC_{50} values (MTT) of entinostat or JQ1. Cells were co-incubated with one fixed concentration of entinostat or JQ1 for 72 h or preincubated with one fixed concentration of entinostat or JQ1 for 48 h followed by addition of the other epigenetic inhibitor for another 72 h. Data shown are the mean ± SD of at least one representative experiment carried out in triplicates. Shift factors (SF) were calculated by dividing the IC_{50} value of the control by the IC_{50} value of the additional treatment.

cell line	control	co-incubation JQ1			control	co-incubation ENT		
	ENT IC_{50} ± SD [µM]	JQ1 [µM]	ENT IC_{50} ± SD [µM]	SF	JQ1 IC_{50} ± SD [µM]	ENT [µM]	JQ1 IC_{50} ± SD [µM]	SF
J82	12.3 ± 1.08	1	7.34 ± 0.77	1.68	21.5 ± 4.05	0.316	25.7 ± 2.71	0.84
J82 cisR	10.4 ± 0.94	0.1	5.27 ± 0.64	1.97	2.64 ± 0.52	0.316	1.54 ± 0.36	1.71

cell line	control	preincubation JQ1 48 h			control	preincubation ENT 48 h		
	ENT IC_{50} ± SD [µM]	JQ1 [µM]	ENT IC_{50} ± SD [µM]	SF	JQ1 IC_{50} ± SD [µM]	ENT [µM]	JQ1 IC_{50} ± SD [µM]	SF
J82	9.59 ± 0.81	1	6.65 ± 0.49	1.44	21.6 ± 4.99	0.316	16.9 ± 2.79	1.28
J82 cisR	9.38 ± 0.89	0.1	6.46 ± 0.73	1.45	2.41 ± 0.51	0.316	1.99 ± 0.37	1.21

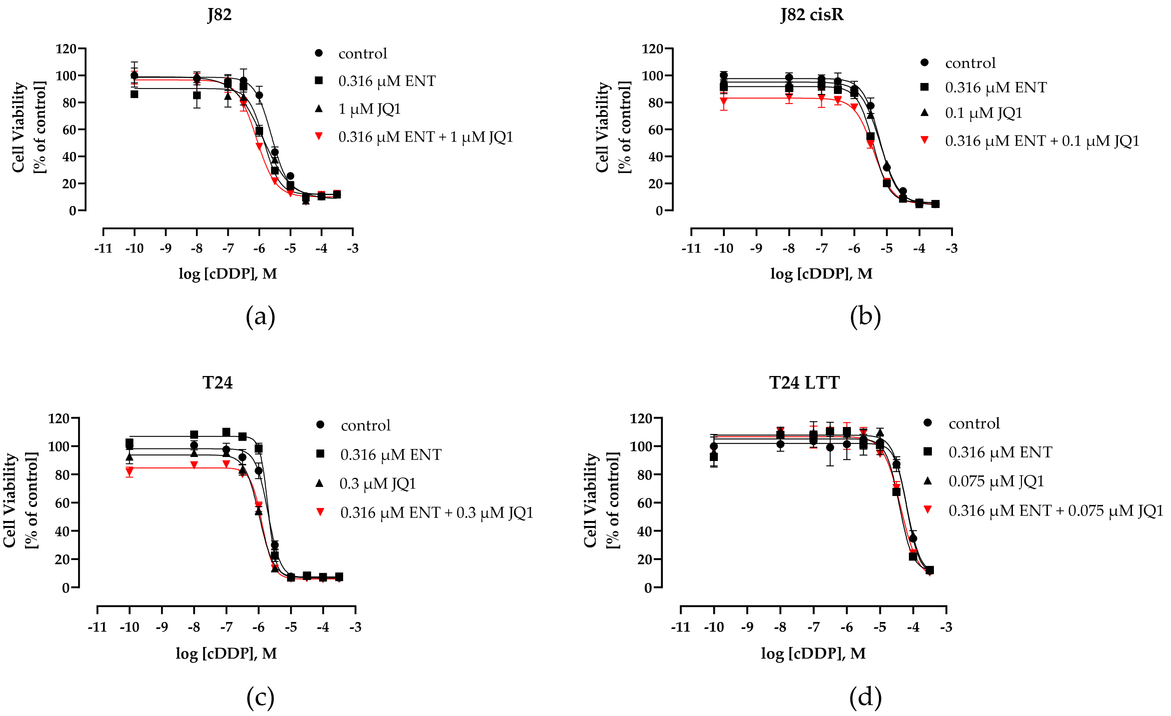

Figure S3. Concentration-effect-curves of cisplatin (MTT-Assay). Cells were co-incubated with entinostat, JQ1 and cisplatin for 72 h. **(a)** J82 **(b)** J82 cisR **(c)** T24 **(d)** T24 LTT. The control illustrates cell viability after treatment with ascending concentrations of cisplatin. Data shown are the mean ± SD of one representative experiment carried out in triplicates.

Table S4. IC_{50} values (MTT) of cisplatin. Cells were co-incubated with entinostat, JQ1 and cisplatin for 72 h. Data shown are the mean ± SD of at least one representative experiment carried out in triplicates.

cell line	control	co-incubation								
	cisplatin IC_{50} ± SD [µM]	ENT [µM]	cisplatin IC_{50} ± SD [µM]	SF	JQ1 [µM]	cisplatin IC_{50} ± SD [µM]	SF	ENT+ JQ1 [µM]	cisplatin IC_{50} ± SD [µM]	SF
**J82**	2.49 ± 0.12	0.316	1.43 ± 0.15	1.74	1	1.31 ± 0.18	1.90	0.316 + 1	0.816 ± 0.06	3.05
**J82 cisR**	6.17 ± 0.23	0.316	3.86 ± 0.18	1.60	0.1	6.30 ± 0.30	0.98	0.316 + 0.1	3.95 ± 0.29	1.56
**T24**	1.97 ± 0.06	0.316	1.94 ± 0.07	1.02	0.3	1.05 ± 0.04	1.88	0.316 + 0.3	1.28 ± 0.04	1.54
**T24 LTT**	65.3 ± 4.17	0.316	38.2 ± 4.06	1.71	0.075	62.8 ± 7.45	1.04	0.316 + 0.075	42.2 ± 4.77	1.55

Table S5. CI values of the dual combination studies (48 h preincubation with entinostat or JQ1, followed by addition of cisplatin for another 72 h. CI values were calculated using compuSyn software from the mean of at least three independent experiments, each carried out in triplicates. CI < 1.0 indicate a synergistic effect (green), CI = 1 indicate an additive effect, CI > 1 indicate an antagonistic effect (red); * means fraction affected was less than 0.20.

J82

cisplatin [µM]	JQ1 [µM] 0.5	1	2	3	ENT [µM] 0.1	0.316	0.5	0.75
0.05	*	*	*	0.804	*	*	*	*
0.1	*	*	1.48	0.800	*	*	*	*
0.15	*	*	1.04	0.733	*	*	*	*
0.2	*	1.80	0.887	0.624	*	*	*	*
0.3	*	0.926	0.509	0.391	*	*	*	3.52

J82 cisR

cisplatin [µM]	JQ1 [µM] 0.05	0.1	0.2	0.3	ENT [µM] 0.1	0.316	0.5	0.75
0.2	*	2.21	1.72	0.683	*	0.934	1.53	1.23
0.4	*	3.54	1.13	0.584	*	*	*	1.38
0.6	*	3.12	1.16	0.383	*	*	*	1.30
0.8	*	2.51	0.828	0.275	*	1.30	1.47	0.989
1	*	1.61	0.533	0.209	*	0.823	1.31	0.701

T24

cisplatin [µM]	JQ1 [µM] 0.1	0.3	0.5	0.7	ENT [µM] 0.316	0.5	0.75
0.2	*	1.15	0.446	0.392	*	*	*
0.4	*	0.730	0.322	0.325	*	*	*

0.6	*	0.522	0.302	0.291	*	*	*
0.8	*	0.419	0.271	0.308	*	*	*
1	*	0.358	0.260	0.277	*	*	*

T24 LTT								
		**JQ1 [µM]**				**ENT [µM]**		
		**0.05**	**0.075**	**0.15**	**0.3**	**0.316**	**0.5**	**0.75**
**cisplatin [µM]**	5	*	*	*	0.955	*	*	1.53
	10	*	*	*	0.803	*	*	1.40
	15	*	*	*	0.759	*	*	0.869
	20	*	*	*	0.565	*	0.910	0.558
	25	*	*	1.85	0.504	0.808	0.632	0.422

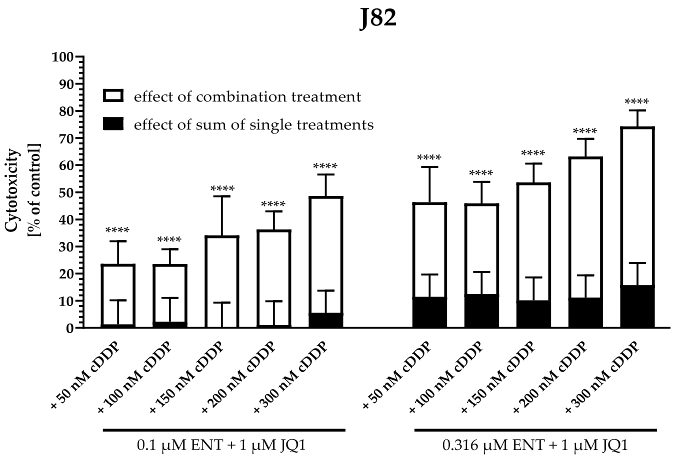

(a)

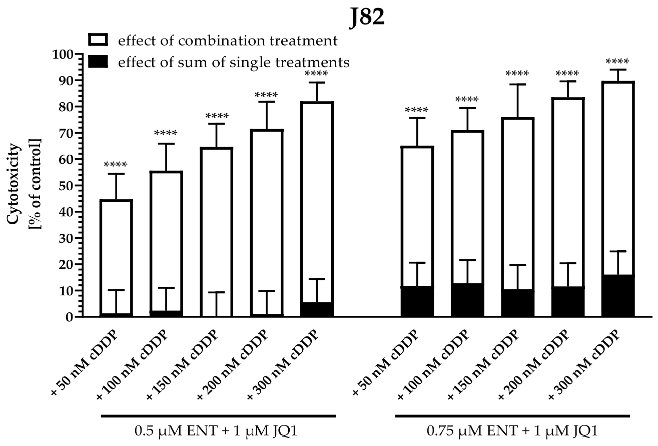

(b)

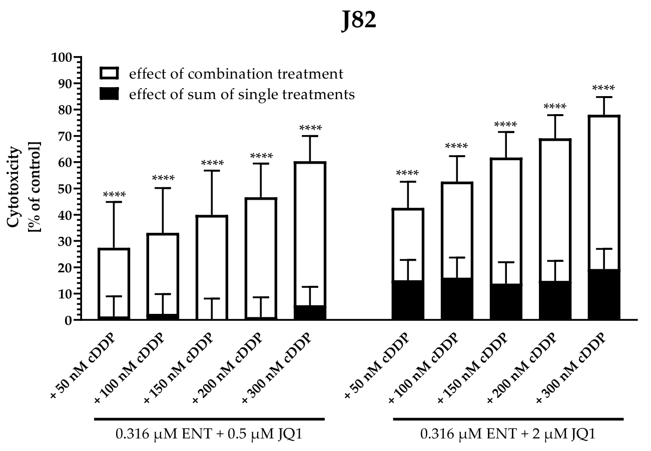
J82
effect of combination treatment
effect of sum of single treatments
Cytotoxicity [% of control]
+ 50 nM cDDP
+ 100 nM cDDP
+ 150 nM cDDP
+ 200 nM cDDP
+ 300 nM cDDP
0.316 μM ENT + 0.5 μM JQ1
0.316 μM ENT + 2 μM JQ1

(c)

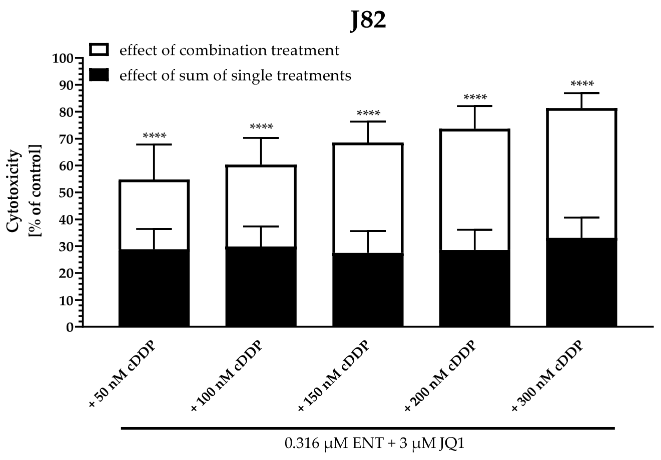
J82
effect of combination treatment
effect of sum of single treatments
Cytotoxicity [% of control]
+ 50 nM cDDP
+ 100 nM cDDP
+ 150 nM cDDP
+ 200 nM cDDP
+ 300 nM cDDP
0.316 μM ENT + 3 μM JQ1

(d)

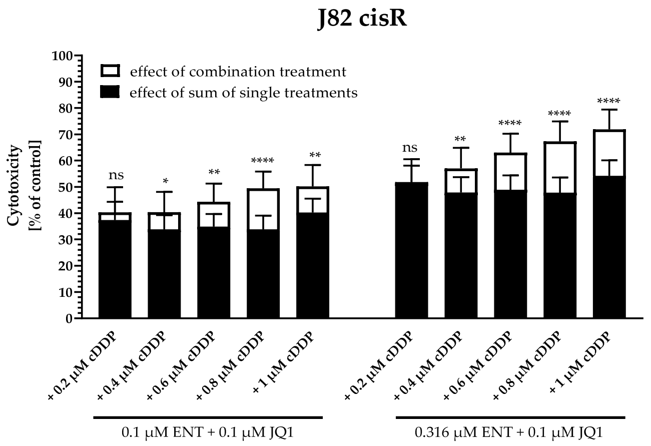
J82 cisR
effect of combination treatment
effect of sum of single treatments
Cytotoxicity [% of control]
+ 0.2 μM cDDP
+ 0.4 μM cDDP
+ 0.6 μM cDDP
+ 0.8 μM cDDP
+ 1 μM cDDP
0.1 μM ENT + 0.1 μM JQ1
0.316 μM ENT + 0.1 μM JQ1

(e)

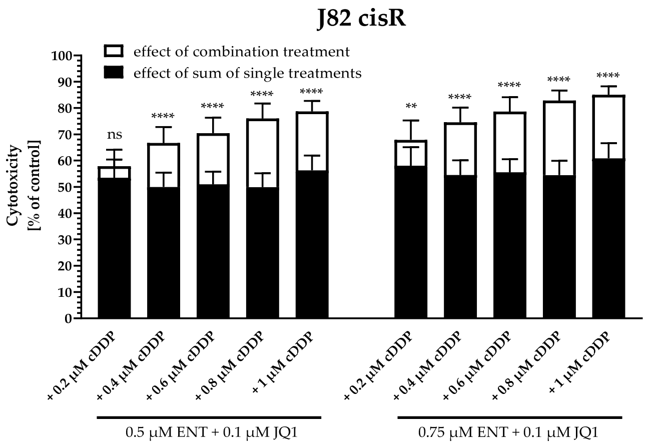
J82 cisR
effect of combination treatment
effect of sum of single treatments
Cytotoxicity [% of control]
+ 0.2 μM cDDP
+ 0.4 μM cDDP
+ 0.6 μM cDDP
+ 0.8 μM cDDP
+ 1 μM cDDP
0.5 μM ENT + 0.1 μM JQ1
0.75 μM ENT + 0.1 μM JQ1

(f)

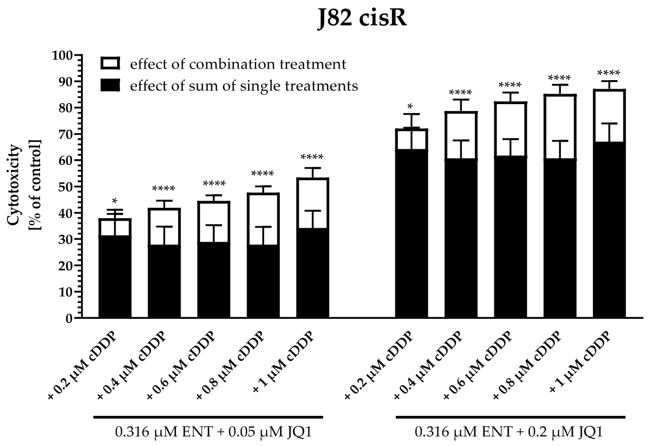
J82 cisR
effect of combination treatment
effect of sum of single treatments
Cytotoxicity [% of control]
+ 0.2 μM cDDP
+ 0.4 μM cDDP
+ 0.6 μM cDDP
+ 0.8 μM cDDP
+ 1 μM cDDP
0.316 μM ENT + 0.05 μM JQ1
0.316 μM ENT + 0.2 μM JQ1

(g)

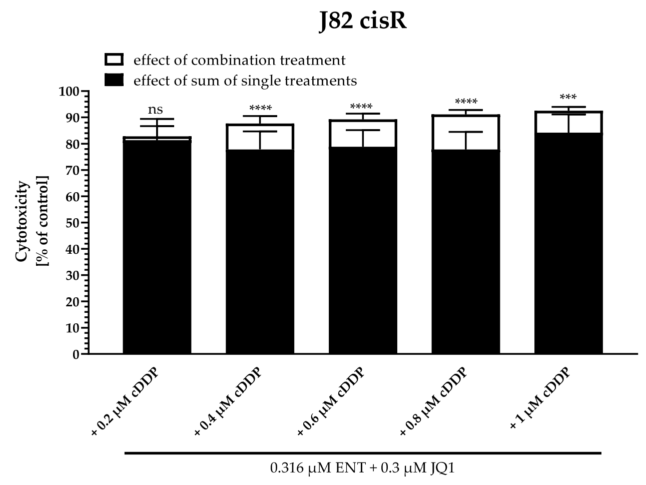
J82 cisR
effect of combination treatment
effect of sum of single treatments
Cytotoxicity [% of control]
+ 0.2 μM cDDP
+ 0.4 μM cDDP
+ 0.6 μM cDDP
+ 0.8 μM cDDP
+ 1 μM cDDP
0.316 μM ENT + 0.3 μM JQ1

(h)

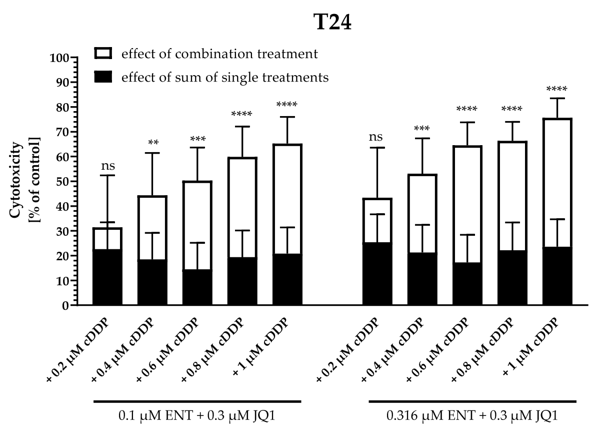
T24
effect of combination treatment
effect of sum of single treatments
Cytotoxicity [% of control]
+ 0.2 µM cDDP
+ 0.4 µM cDDP
+ 0.6 µM cDDP
+ 0.8 µM cDDP
+ 1 µM cDDP
0.1 µM ENT + 0.3 µM JQ1
0.316 µM ENT + 0.3 µM JQ1

(i)

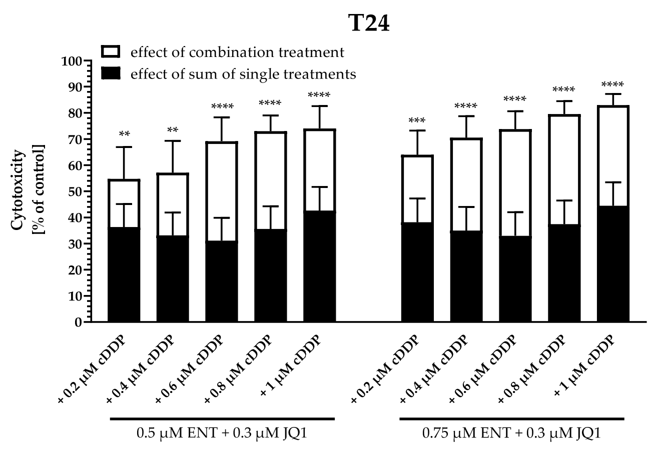
T24
effect of combination treatment
effect of sum of single treatments
Cytotoxicity [% of control]
+ 0.2 µM cDDP
+ 0.4 µM cDDP
+ 0.6 µM cDDP
+ 0.8 µM cDDP
+ 1 µM cDDP
0.5 µM ENT + 0.3 µM JQ1
0.75 µM ENT + 0.3 µM JQ1

(j)

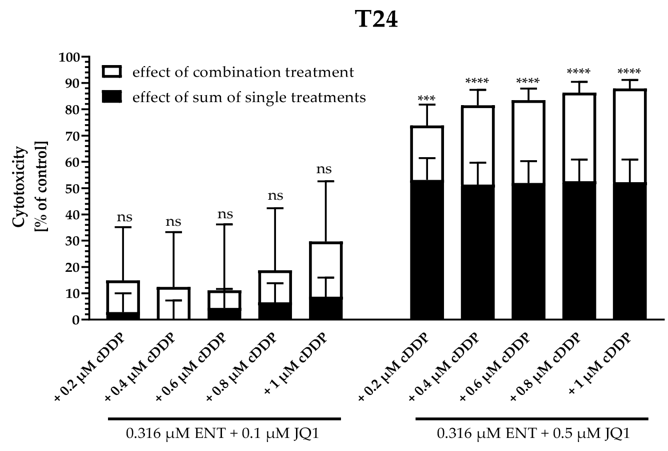
T24
effect of combination treatment
effect of sum of single treatments
Cytotoxicity [% of control]
+ 0.2 µM cDDP
+ 0.4 µM cDDP
+ 0.6 µM cDDP
+ 0.8 µM cDDP
+ 1 µM cDDP
0.316 µM ENT + 0.1 µM JQ1
0.316 µM ENT + 0.5 µM JQ1

(k)

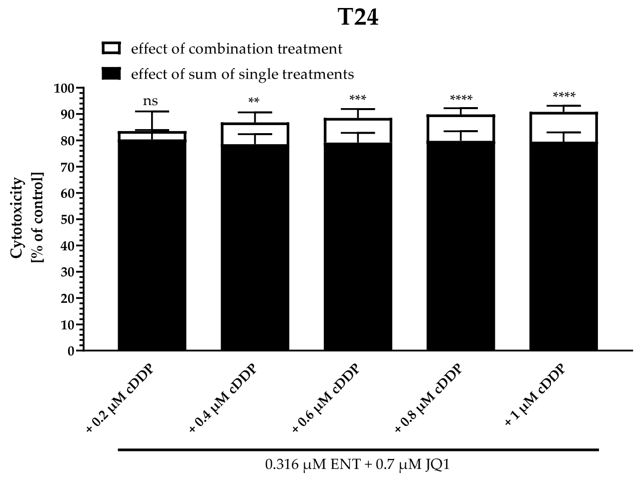
T24
effect of combination treatment
effect of sum of single treatments
Cytotoxicity [% of control]
+ 0.2 µM cDDP
+ 0.4 µM cDDP
+ 0.6 µM cDDP
+ 0.8 µM cDDP
+ 1 µM cDDP
0.316 µM ENT + 0.7 µM JQ1

(l)

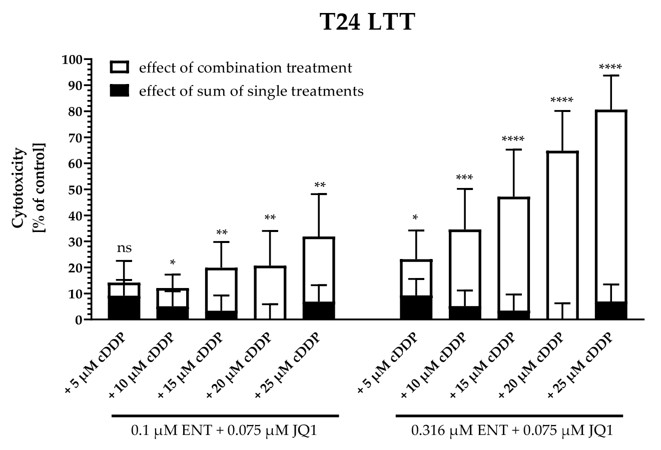
T24 LTT
effect of combination treatment
effect of sum of single treatments
Cytotoxicity [% of control]
+ 5 µM cDDP
+ 10 µM cDDP
+ 15 µM cDDP
+ 20 µM cDDP
+ 25 µM cDDP
0.1 µM ENT + 0.075 µM JQ1
0.316 µM ENT + 0.075 µM JQ1

(m)

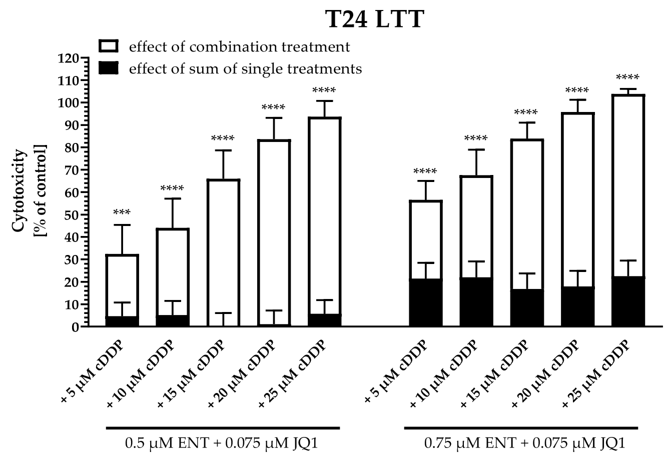
T24 LTT
effect of combination treatment
effect of sum of single treatments
Cytotoxicity [% of control]
+ 5 µM cDDP
+ 10 µM cDDP
+ 15 µM cDDP
+ 20 µM cDDP
+ 25 µM cDDP
0.5 µM ENT + 0.075 µM JQ1
0.75 µM ENT + 0.075 µM JQ1

(n)

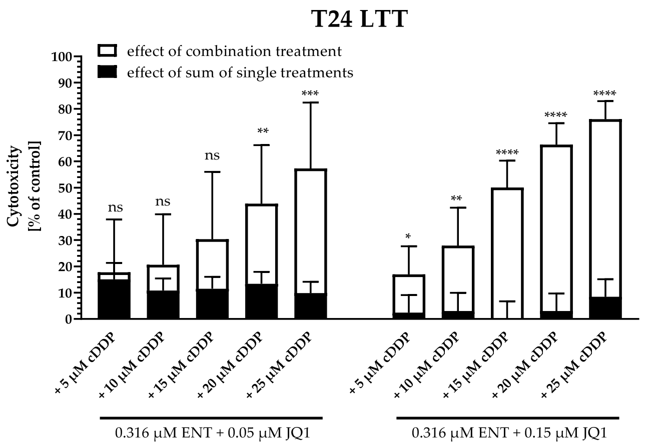

(o)

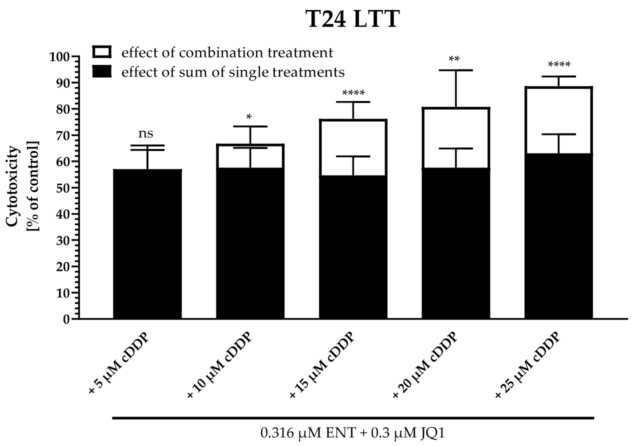

(p)

Figure S4. Synergistic cytotoxic effect of combination treatment in J82 **(a)**-**(d)**, J82 cisR **(e)**-**(h)**, T24 **(i)**-**(l)** and T24 LTT **(m)**-**(p)**. Cells were preincubated with entinostat and JQ1 for 48 h followed by an addition of cisplatin for another 72 h. Shown are the mean + SD of at least three independent experiments, each carried out in triplicates. Data were normalized to untreated control. Black bars illustrate the additive effects (sum) of the individual treatments, white bars are the data of the combination treatment. Significance was calculated using unpaired t-test. ns ($p \geq 0.05$); * ($p < 0.05$); ** ($p < 0.01$); *** ($p < 0.001$); **** ($p < 0.0001$).

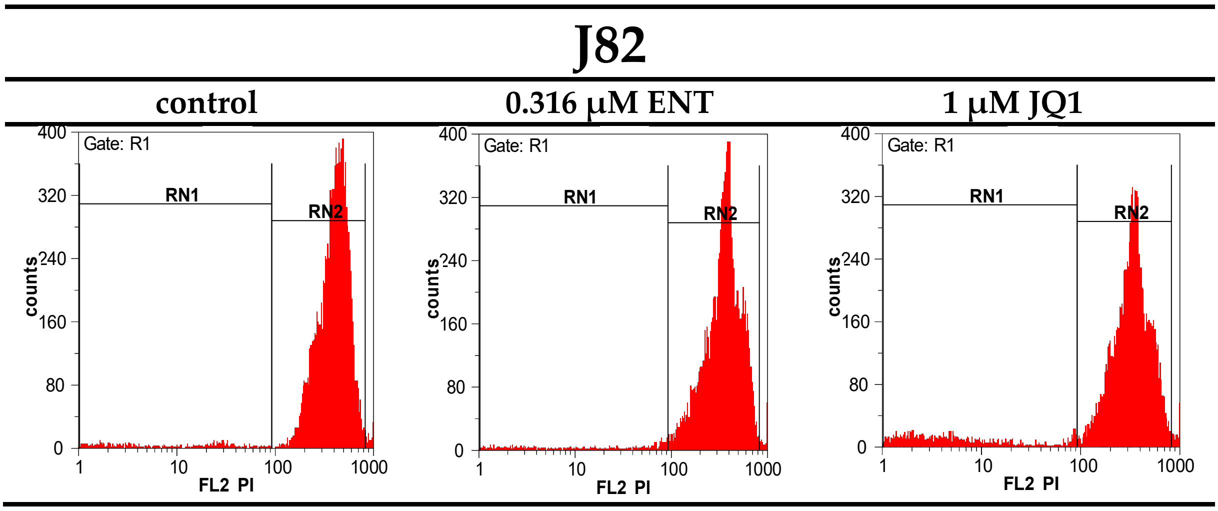

2 µM cisplatin

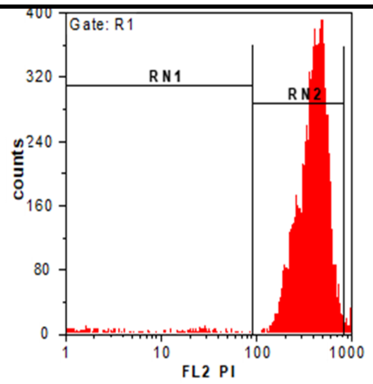

0.316 µM ENT + 1 µM JQ1

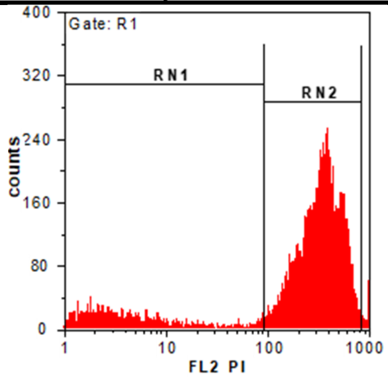

0.316 µM ENT + 2 µM cisplatin

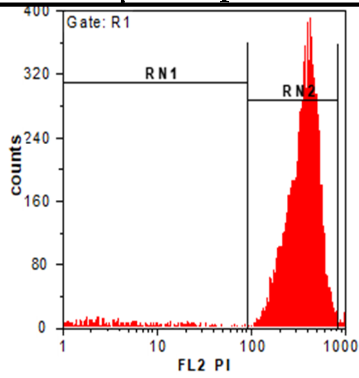

1 µM JQ1 + 2 µM cisplatin

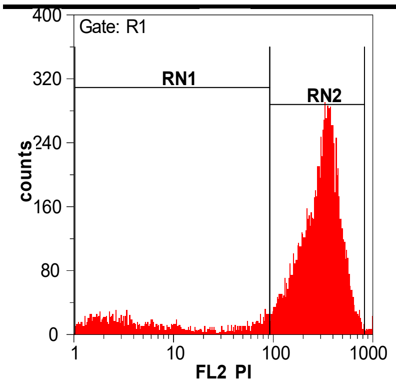

0.316 µM ENT + 1 µM JQ1 + 2 µM cisplatin

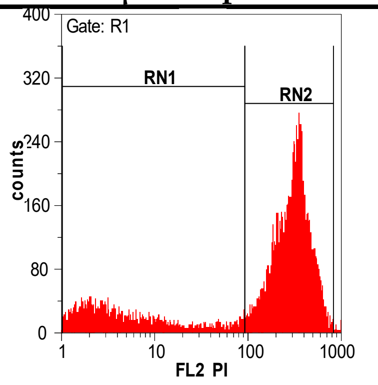

100 µM cisplatin

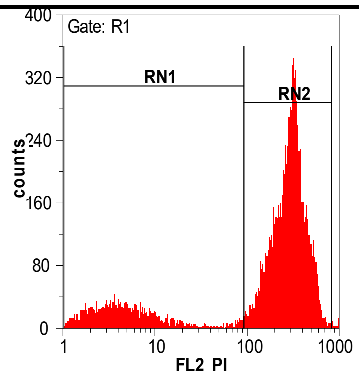

J82 cisR

control

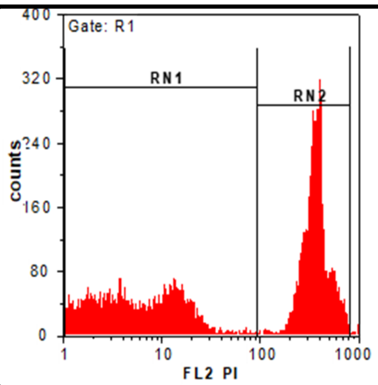

0.316 µM ENT

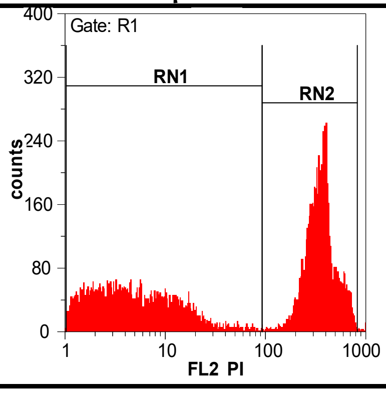

0.1 µM JQ1

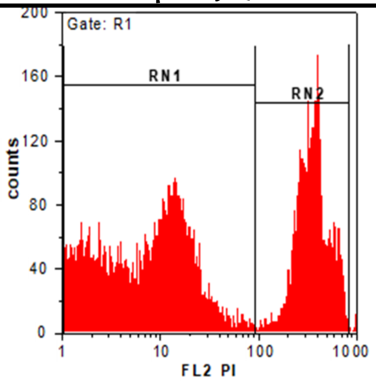

8 µM cisplatin

0.316 µM ENT + 0.1 µM JQ1

0.316 µM ENT + 8 µM cisplatin

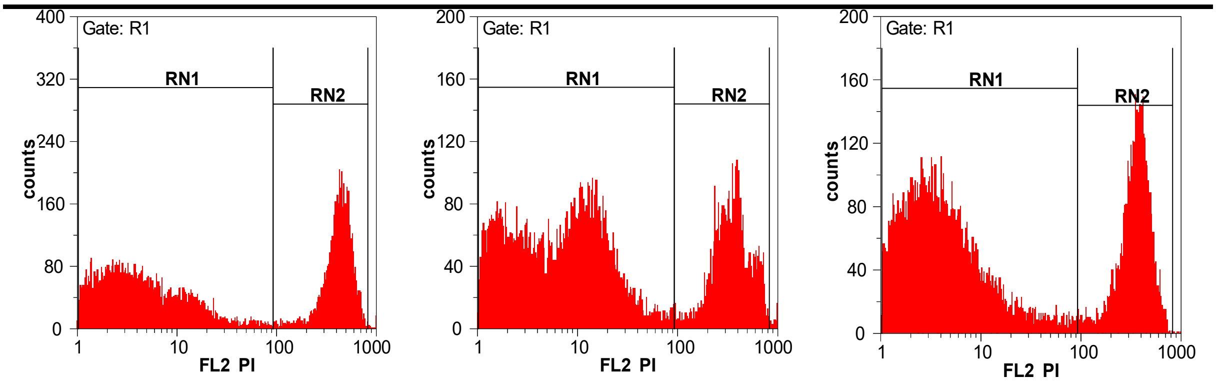

0.1 µM JQ1<br>+ 8 µM cisplatin	0.316 µM ENT<br>+ 0.1 µM JQ1<br>+ 8 µM cisplatin	100 µM cisplatin

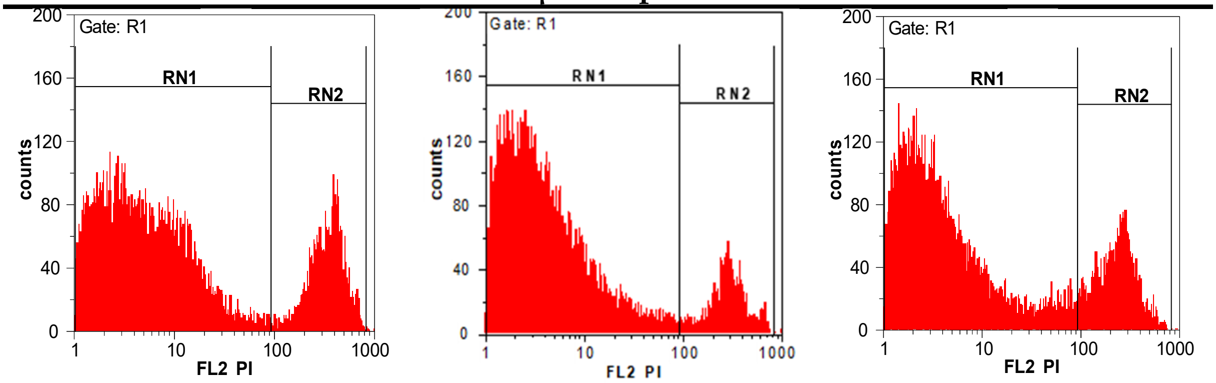

T24

control	0.316 µM ENT	0.3 µM JQ1

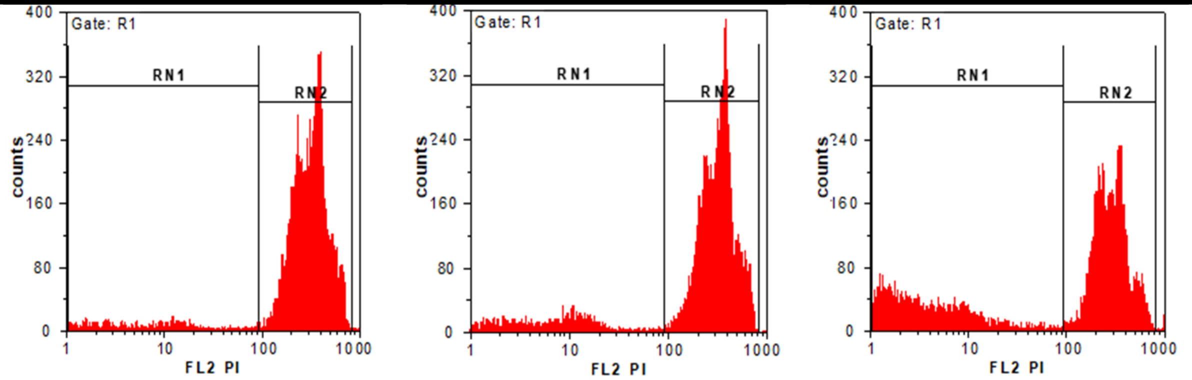

3 µM cisplatin	0.316 µM ENT<br>+ 0.3 µM JQ1	0.316 µM ENT<br>+ 3 µM cisplatin

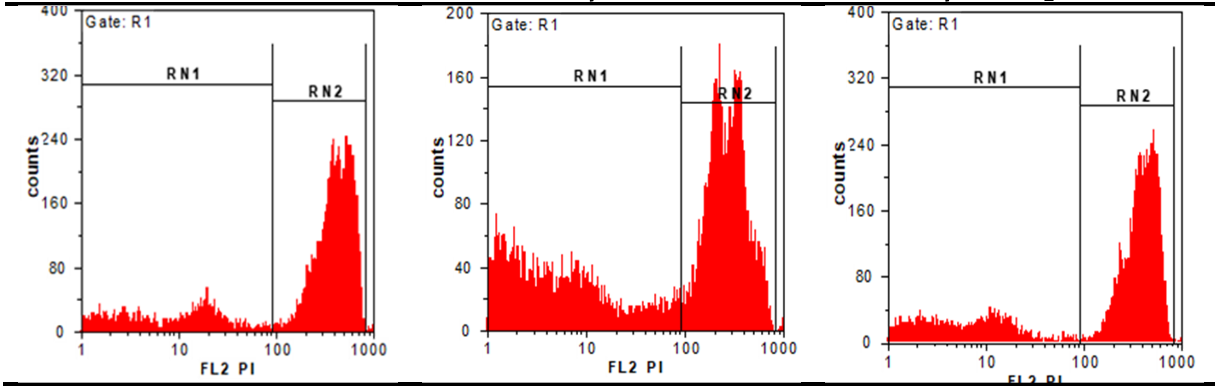

0.3 μM JQ1 + 3 μM cisplatin	0.316 μM ENT + 0.3 μM JQ1 + 3 μM cisplatin	100 μM cisplatin

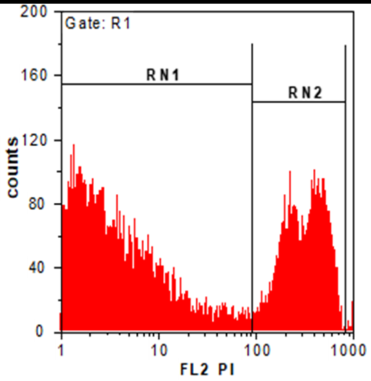

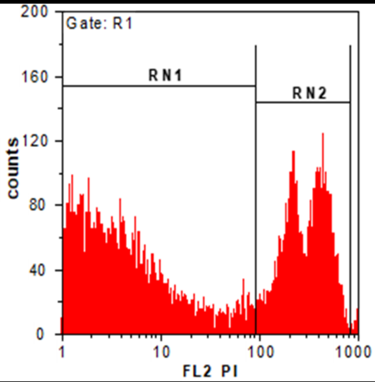

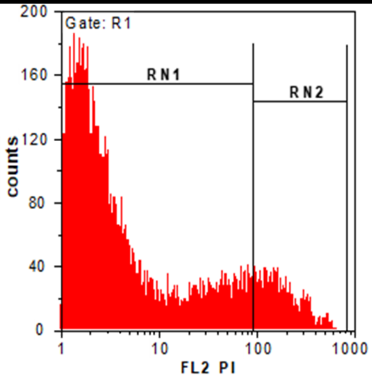

T24 LTT

control	0.316 μM ENT	0.075 μM JQ1

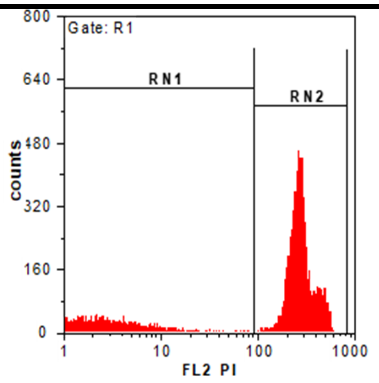

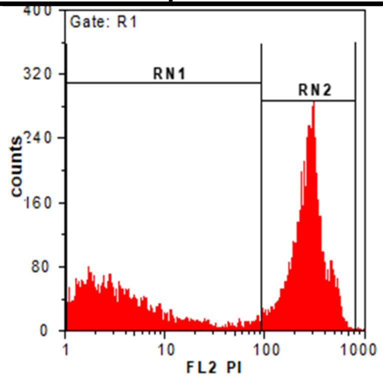

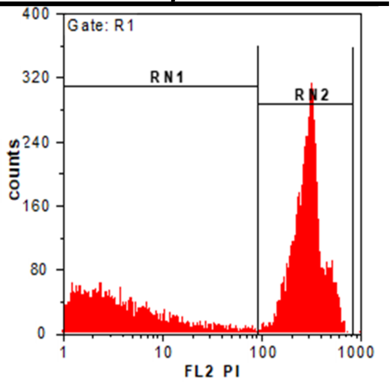

70 μM cisplatin	0.316 μM ENT + 0.075 μM JQ1	0.316 μM ENT + 70 μM cisplatin

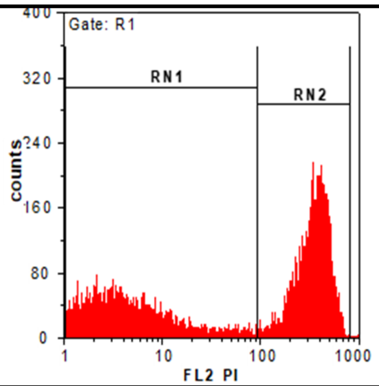

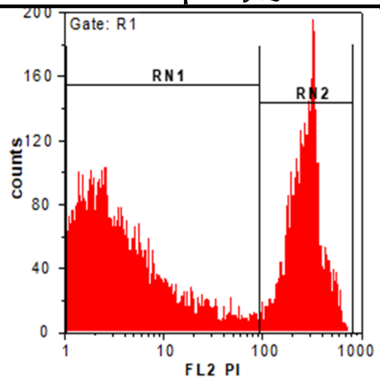

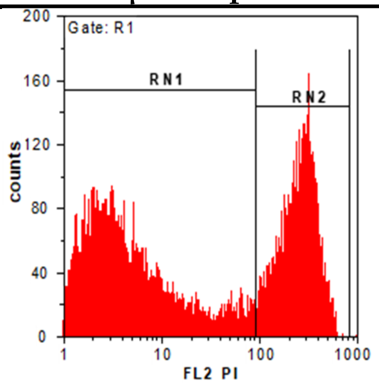

0.075 μM JQ1 + 70 μM cisplatin	0.316 μM ENT + 0.075 μM JQ1 + 70 μM cisplatin	200 μM cisplatin

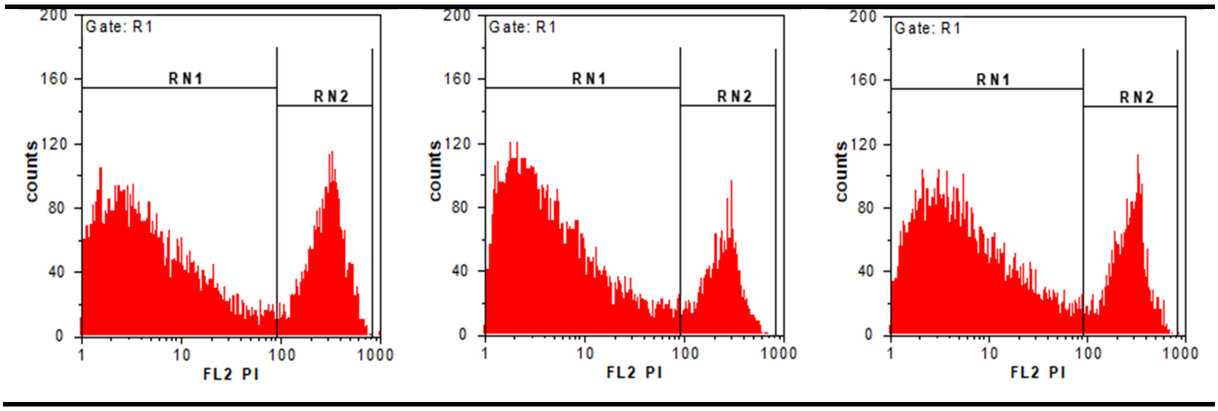

Figure S5. Representative cytometry flow images are shown for control, entinostat, JQ1, cisplatin and their combination treatments in J82, J82 cisR, T24 and T24 LTT. Cells were preincubated with entinostat and JQ1 for 48 h followed by addition of cisplatin for another 24 h. RN1 was defined as the subG1 fraction.

J82

vehicle control	0.316 μM ENT	1 μM JQ1

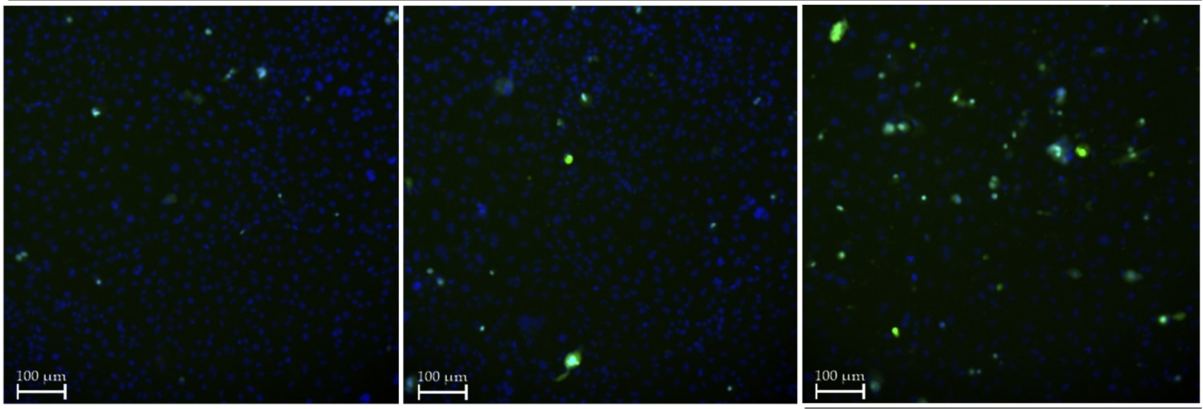

0.316 μM ENT + 1 μM JQ1	20 μM cisplatin	100 μM cisplatin

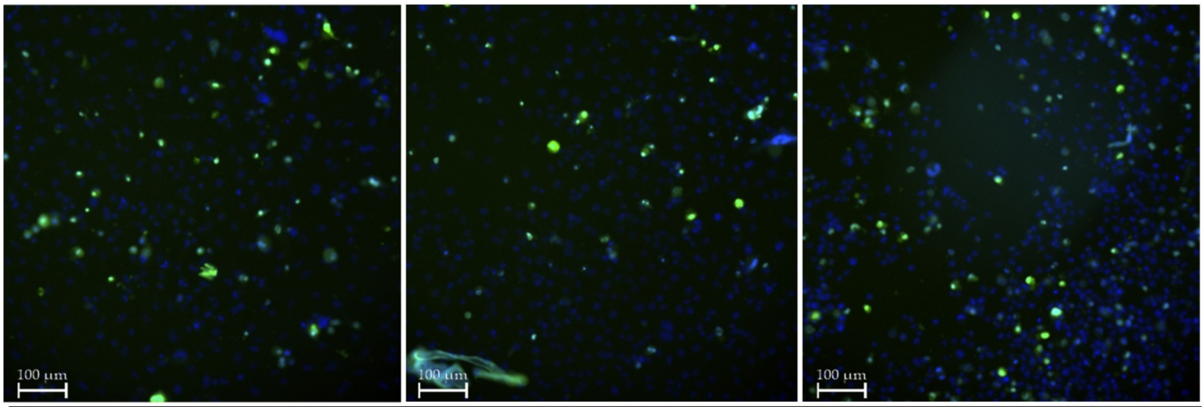

0.316 μM ENT + 20 μM cisplatin	1 μM JQ1 + 20 μM cisplatin	0.316 μM ENT + 1 μM JQ1 + 20 μM cisplatin

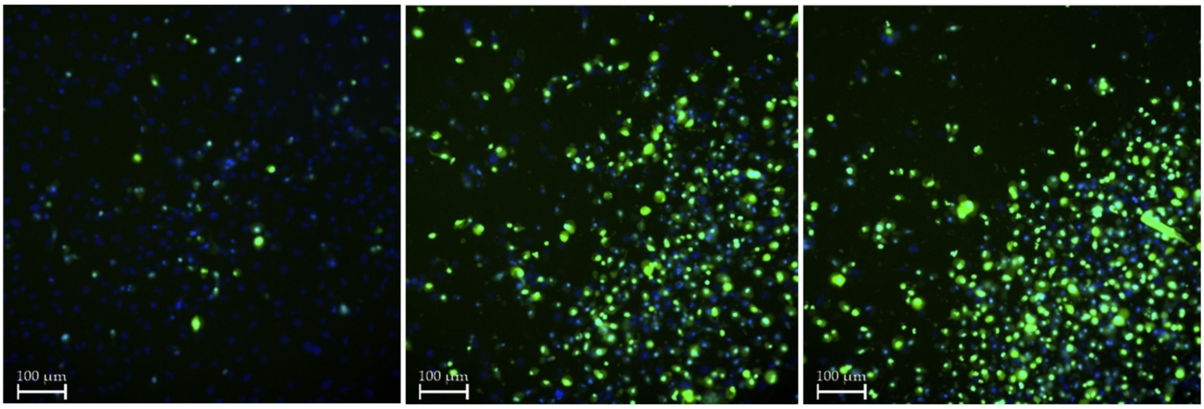

J82 cisR

vehicle control

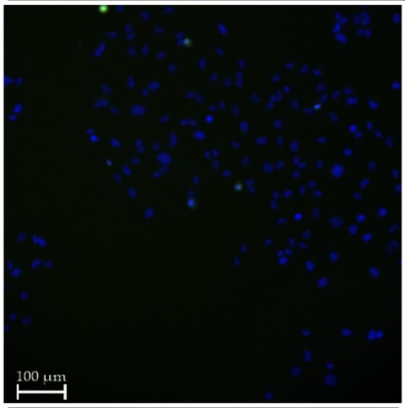

0.316 μM ENT

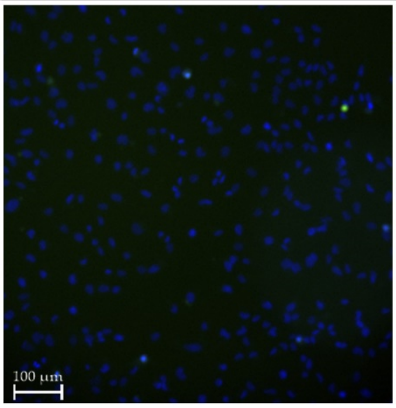

0.1 μM JQ1

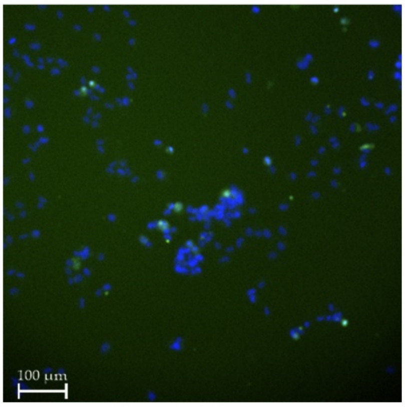

0.316 μM ENT + 0.1 μM JQ1

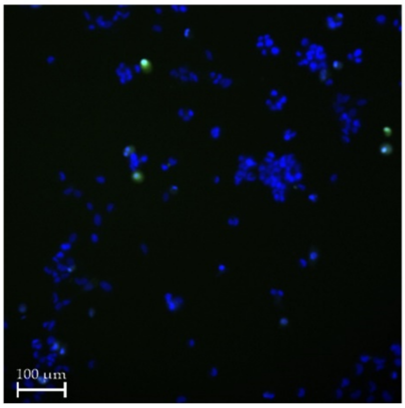

60 μM cisplatin

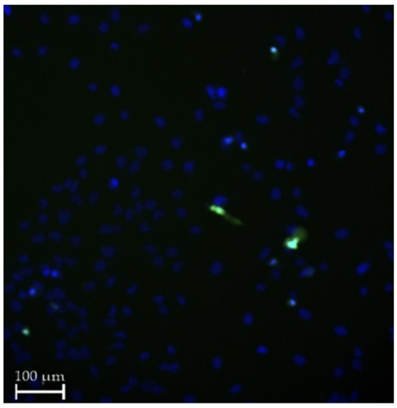

100 μM cisplatin

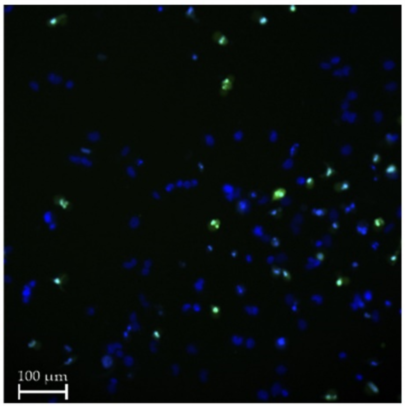

0.316 μM ENT + 60 μM cisplatin

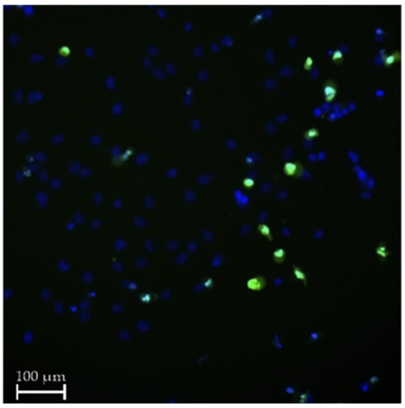

0.1 μM JQ1 + 60 μM cisplatin

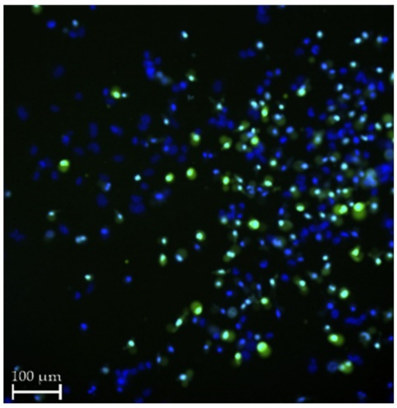

0.316 μM ENT + 0.1 μM JQ1 + 60 μM cisplatin

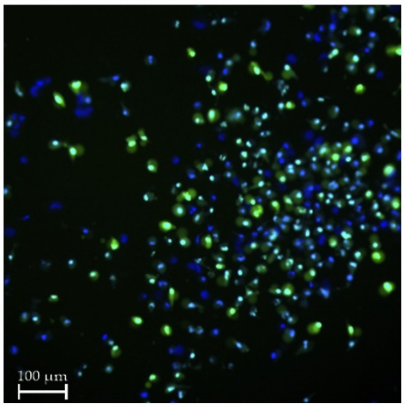

T24

vehicle control	0.316 μM ENT	0.3 μM JQ1

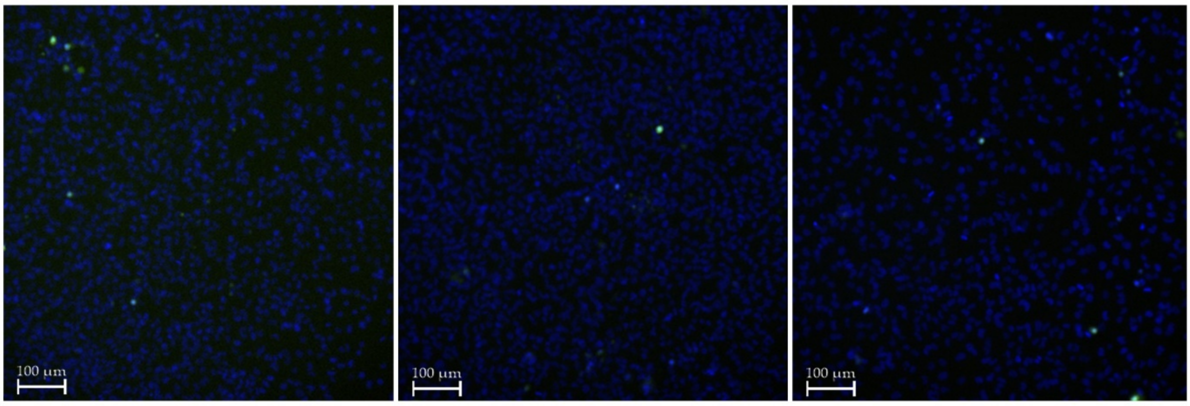

0.316 μM ENT + 0.3 μM JQ1	15 μM cisplatin	100 μM cisplatin

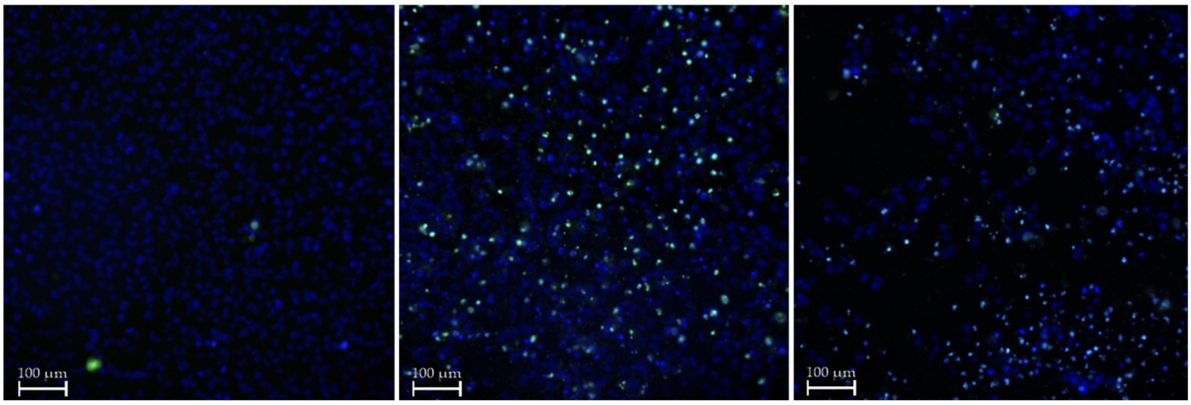

0.316 μM ENT + 15 μM cisplatin	0.3 μM JQ1 + 15 μM cisplatin	0.316 μM ENT + 0.3 μM JQ1 + 15 μM cisplatin

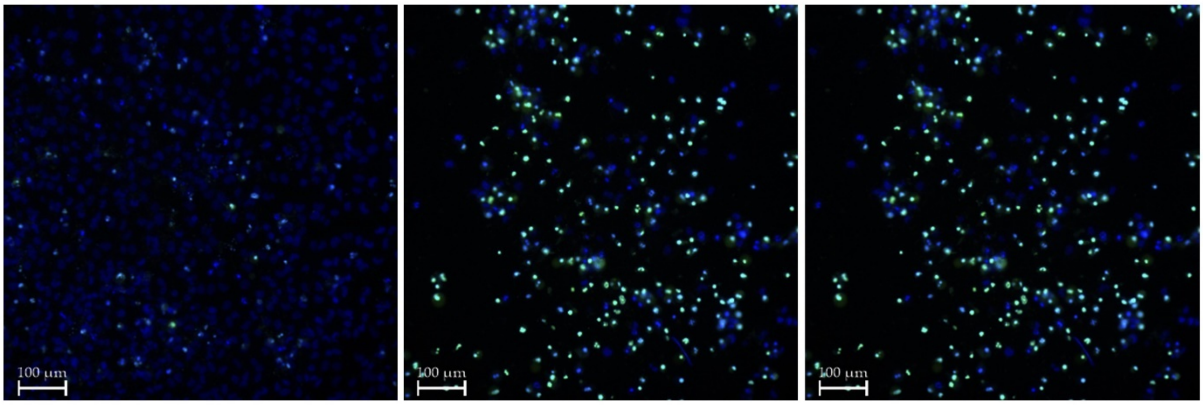

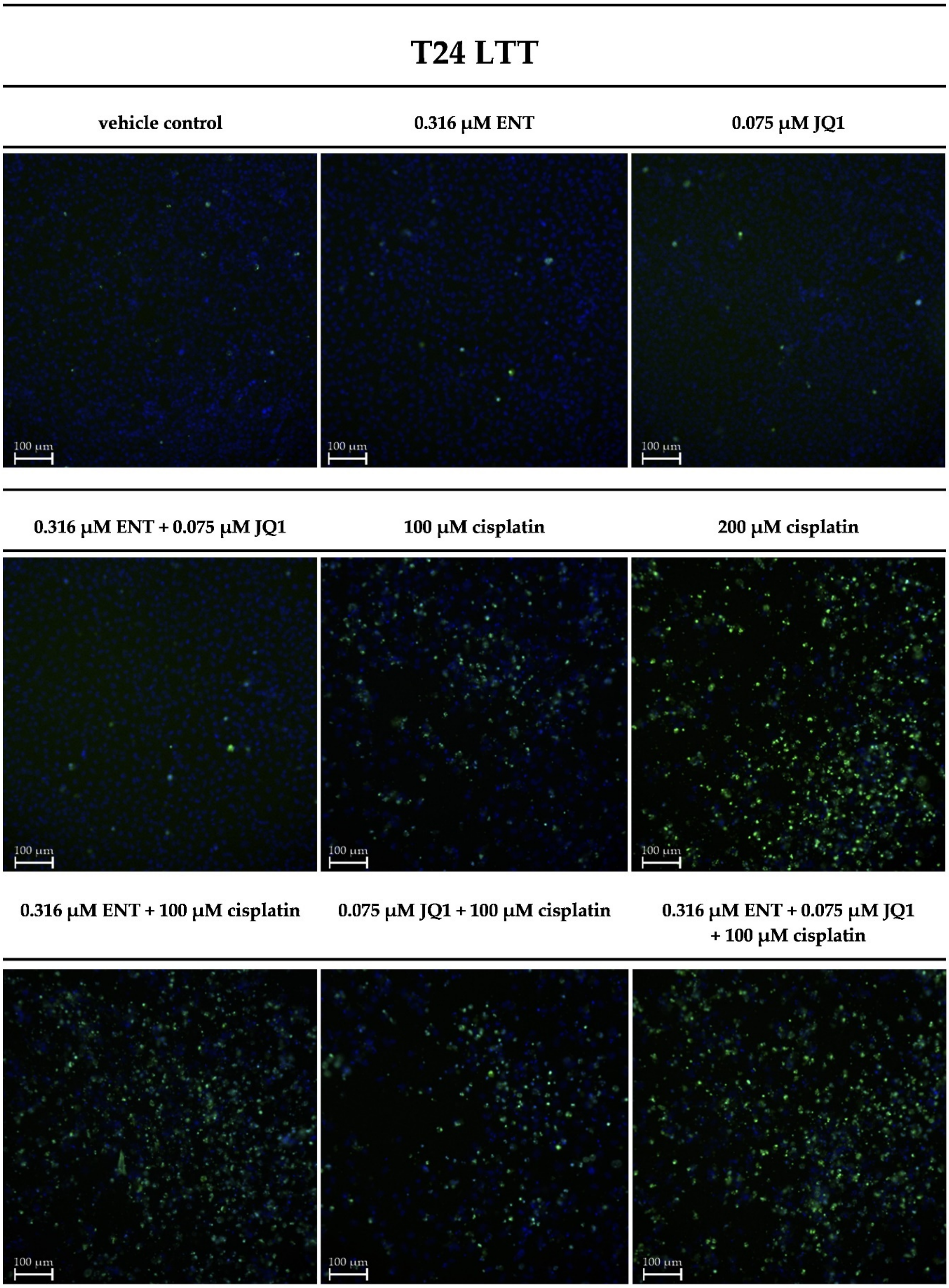

Figure S6. Representative fluorescent imaging pictures (Thermofisher Arrayscan XTI) are shown for vehicle control, entinostat, JQ1, cisplatin and their combination treatments in J82, J82 cisR, T24 and T24 LTT. Nuclei were stained using Hoechst 33342 (blue). Caspase 3/7 activation is shown in green. Cells were preincubated with entinostat and JQ1 for 48 h followed by addition of cisplatin for another 24 h (T24 LTT 48 h).

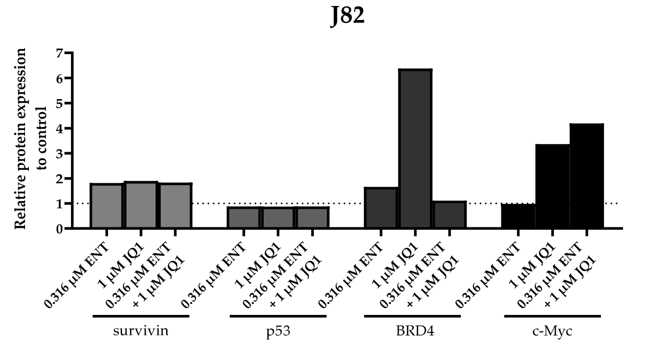

(a)

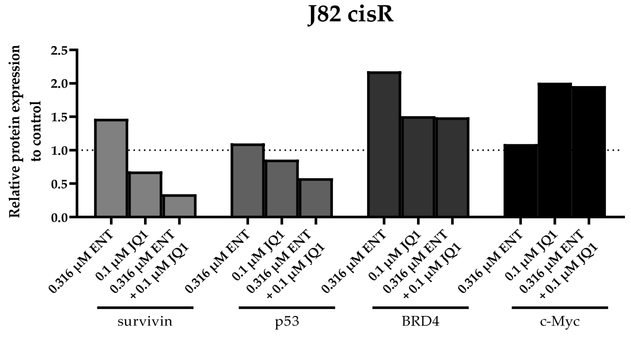

(b)

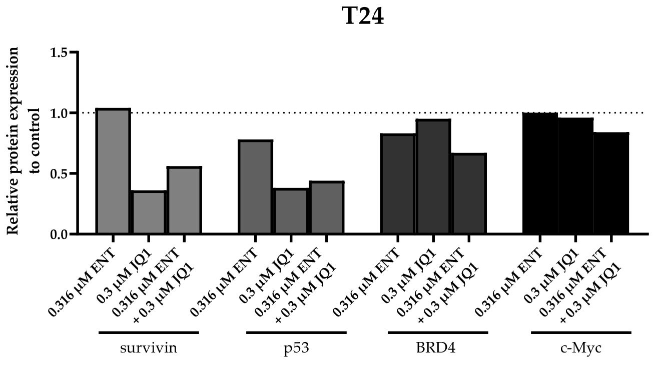

(c)

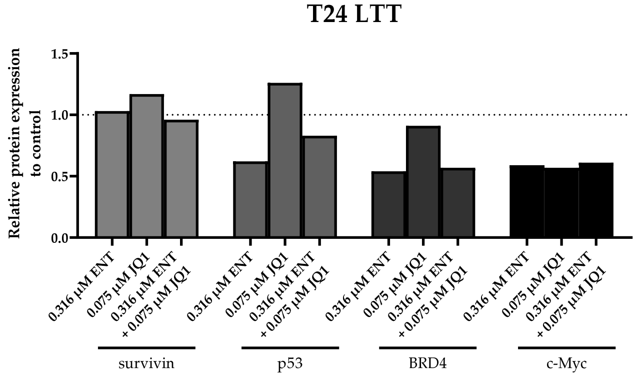

(d)

Figure S7. The relative protein expression of proteins of interest related to β-actin as loading control is shown in **(a)** J82, **(b)** J82 cisR, **(c)** T24 and **(d)** T24 LTT. Ratio of control was set to 1, represented by the dashed line. The western blots were analyzed with ImageJ software (ImageJ 1.54g (http://imagej.org). Data shown is one representative blot out of at least two different protein samples. Cells were incubated with entinostat and JQ1 for 48 h.

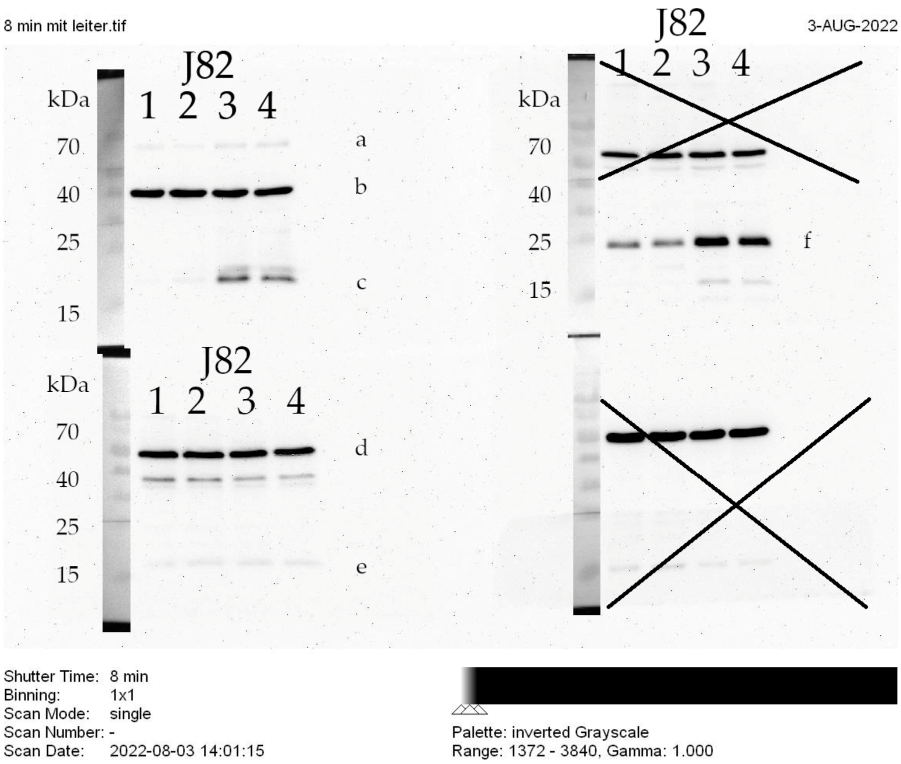

a 1: 0.57: 1.54: 1.71
c 1: 2.98: 41.8: 32.8
d 1: 0.88: 0.87: 0.88
e 1: 1.81: 1.90: 1.83

f 1: 0.59: 1.82: 1.38

Figure S8. Uncropped western blot, J82: blot at left top shows FOXO1 (a), β-Actin (b) and p21 (c) and the blot below illustrates p53 (d) and survivin (e), the blot on the right-side displays Bim (f), 48 h incubation. Order of samples left to right: control (1), entinostat (2), JQ1 (3), entinostat plus JQ1 (4). Densiometric analysis with ImageJ software of relative protein expression in relation to β-Actin is presented (a; c-f). Ratio of control was set to 1.

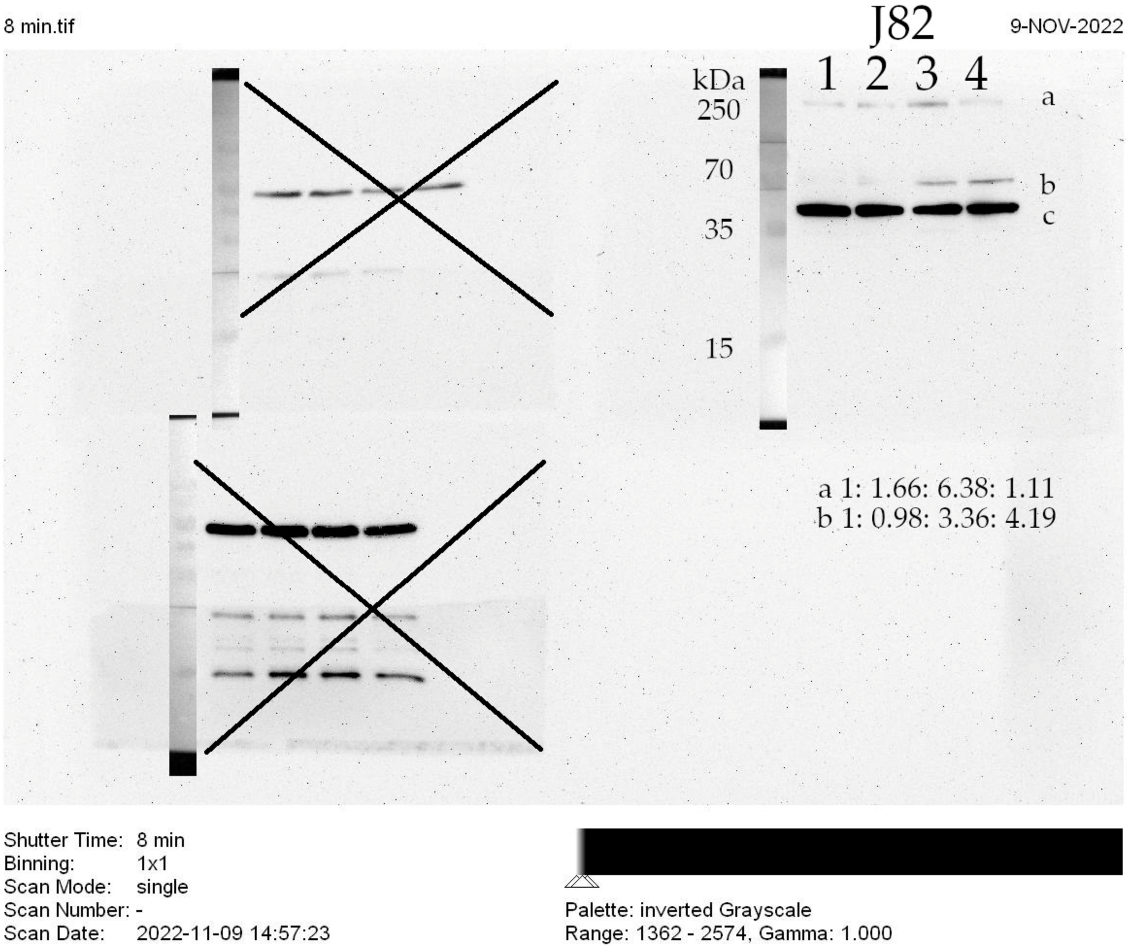

Figure S9. Uncropped western blot, J82: blot at right top shows BRD4 (a), c-Myc (b) and β-Actin (c), 48 h incubation. Order of samples left to right: control (1), entinostat (2), JQ1 (3), entinostat plus JQ1 (4). Densiometric analysis with ImageJ software of relative protein expression in relation to β-Actin is presented (a-b). Ratio of control was set to 1.

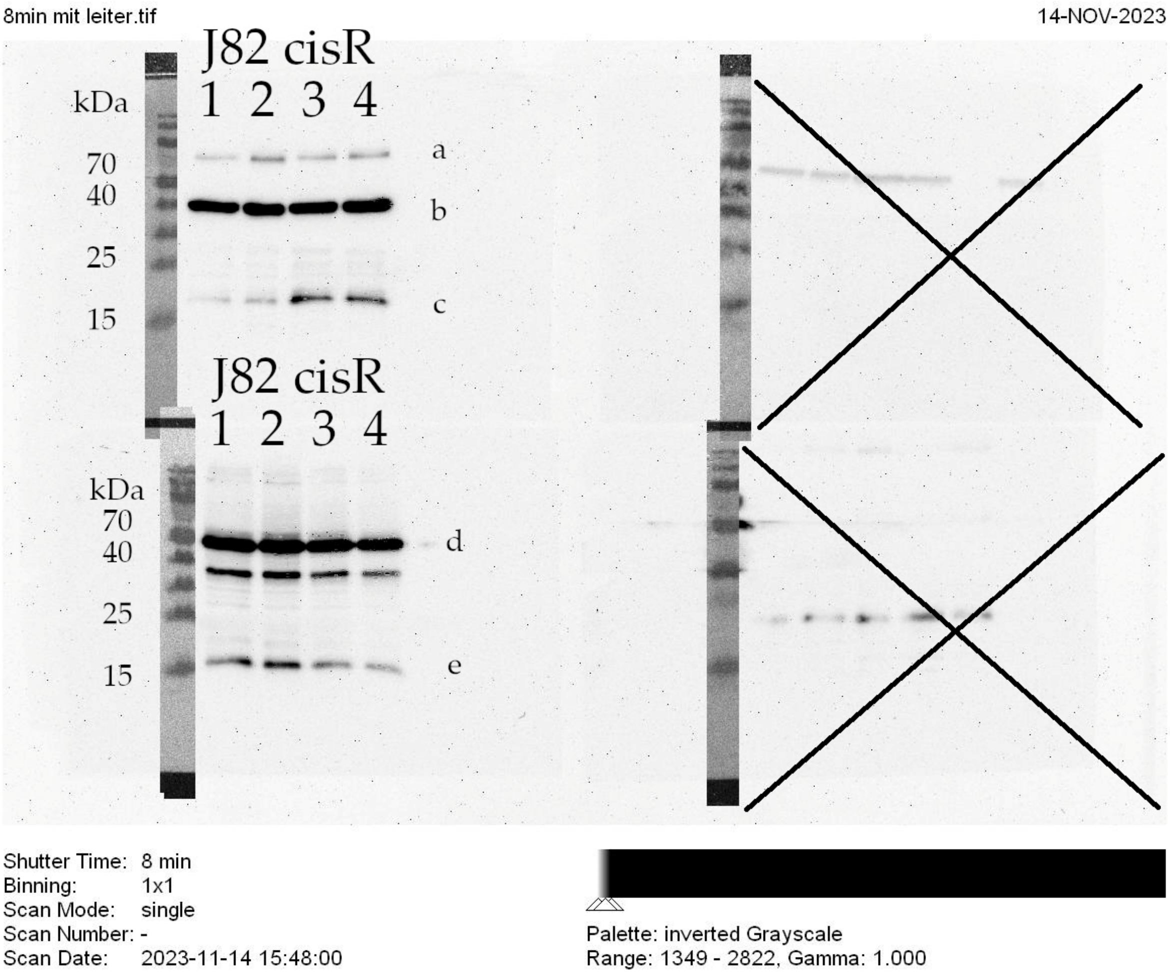

a 1: 3.13: 1.79: 2.02
c 1: 2.20: 9.75: 6.83
d 1: 1.10: 0.86: 0.58
e 1: 1.47: 0.68: 0.34

Figure S10. Uncropped western blot, J82 cisR: blot at left top shows FOXO1 (a), β-Actin (b) and p21 (c), the blot below illustrates p53 (d) and survivin (e), 48 h incubation. Order of samples left to right: control (1), entinostat (2), JQ1 (3), entinostat plus JQ1 (4). Densiometric analysis with ImageJ software of relative protein expression in relation to β-Actin is presented (a; c-e). Ratio of control was set to 1.

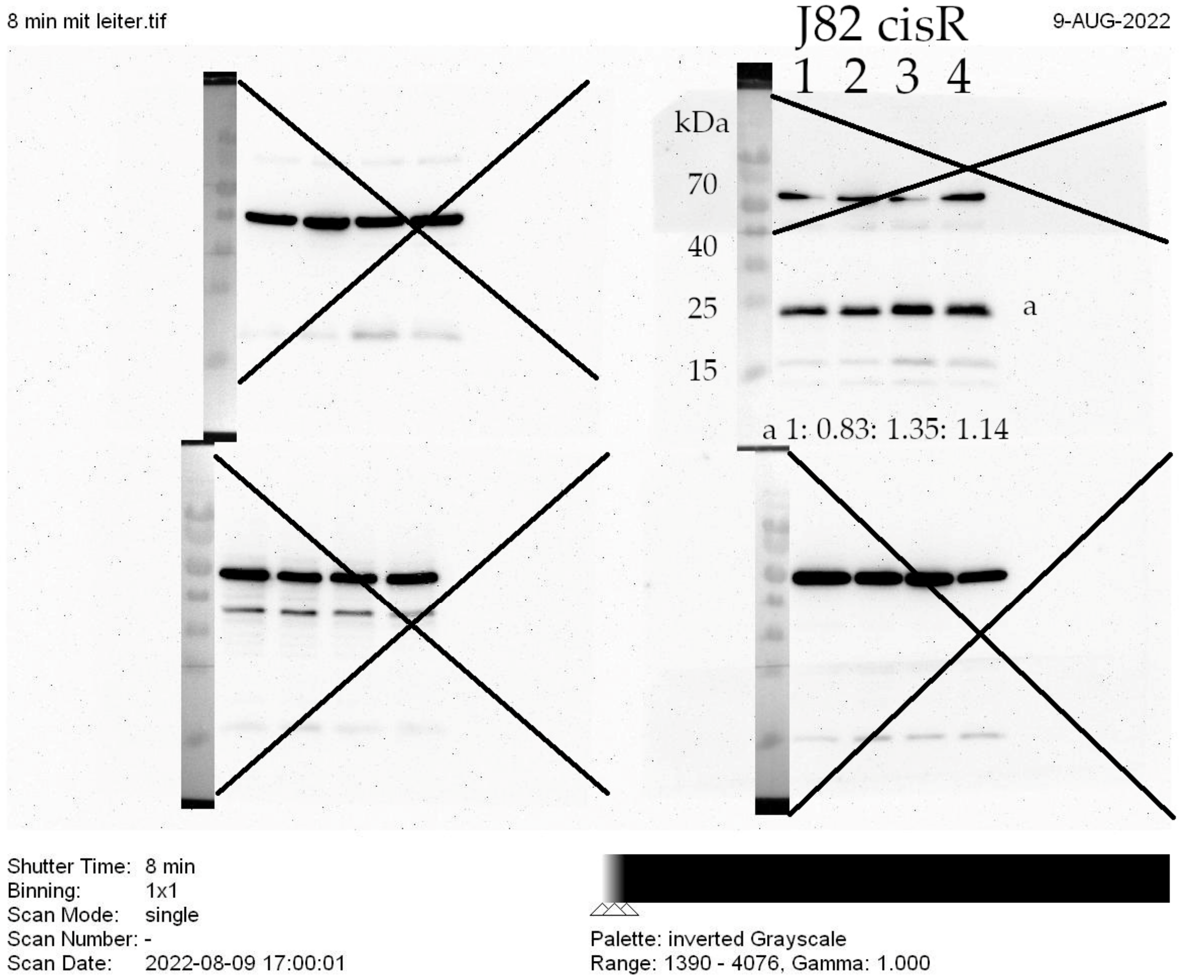

Figure S11. Uncropped western blot, J82 cisR: blot at right top shows at the bottom Bim (a), 48 h incubation. Order of samples left to right: control (1), entinostat (2), JQ1 (3), entinostat plus JQ1 (4). Densiometric analysis with ImageJ software of relative protein expression in relation to β-Actin is presented (a). Ratio of control was set to 1.

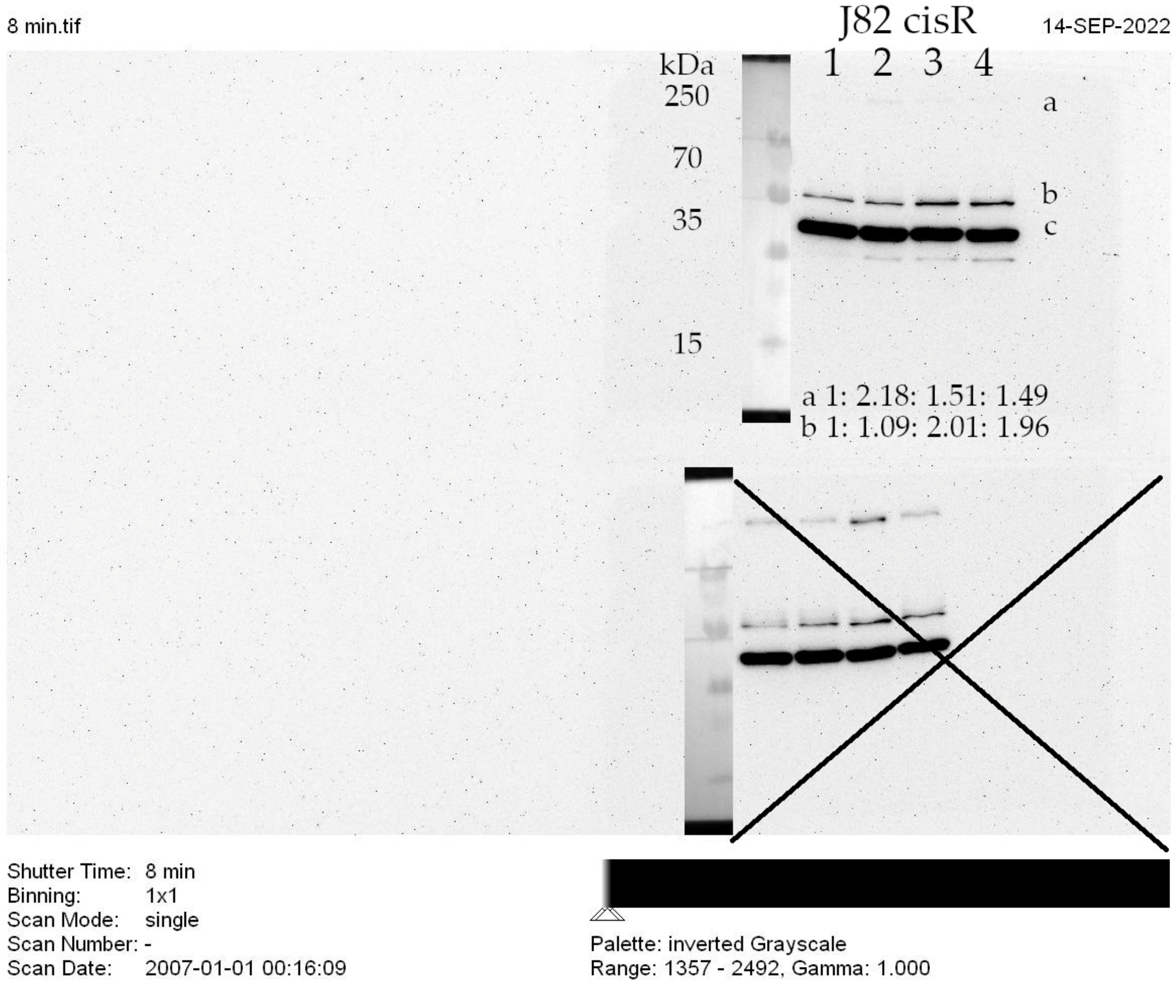

Figure S12. Uncropped western blot, J82 cisR: blot at top shows BRD (a), c-Myc (b) and β-Actin (c), 48 h incubation. Order of samples left to right: control (1), entinostat (2), JQ1 (3), entinostat plus JQ1 (4). Densiometric analysis with ImageJ software of relative protein expression in relation to β-Actin is presented (a-b). Ratio of control was set to 1.

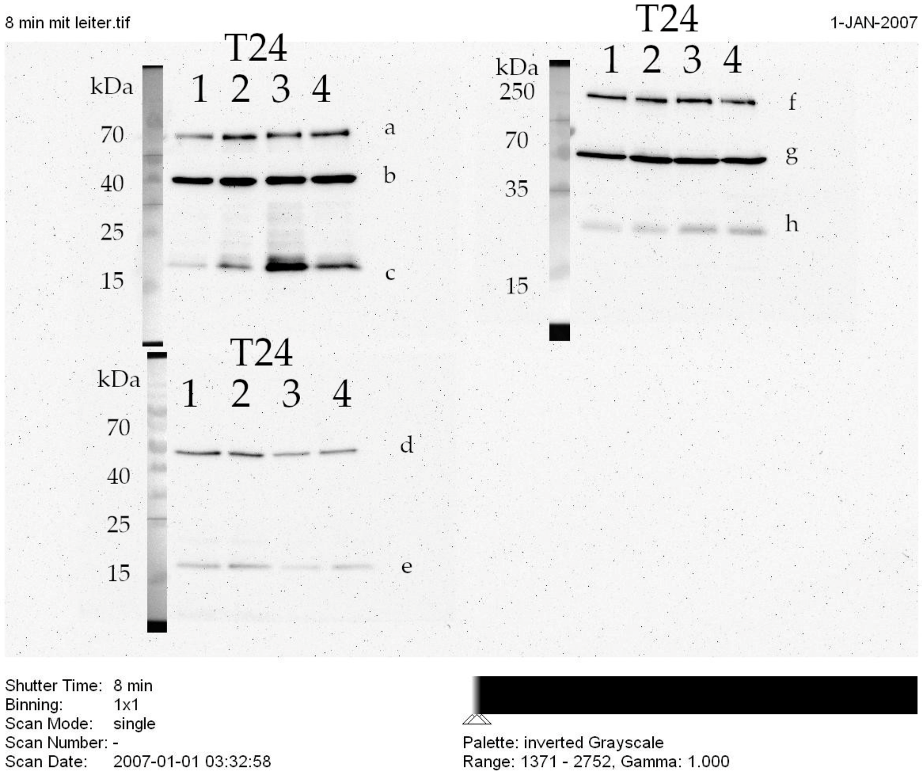

a 1: 1.84: 1.42: 2.31
c 1: 2.20: 16.0: 6.56
d 1: 0.78: 0.38: 0.44
e 1: 1.04: 0.36: 0.56

f 1: 0.83: 0.95: 0.67
g 1: 1.00: 0.96: 0.84
h 1: 1.12: 1.76: 1.64

Figure S13. Uncropped western blot, T24: blot at left top shows FOXO1 (a), β-Actin (b) and p21 (c), the blot below illustrates p53 (d) and survivin (e), the blot on the right-side displays BRD4 (f), c-Myc (g) and Bim (h), 48 h incubation. Order of samples left to right: control (1), entinostat (2), JQ1 (3), entinostat plus JQ1 (4). Densiometric analysis with ImageJ software of relative protein expression in relation to β-Actin is presented (a; c-h). Ratio of control was set to 1.

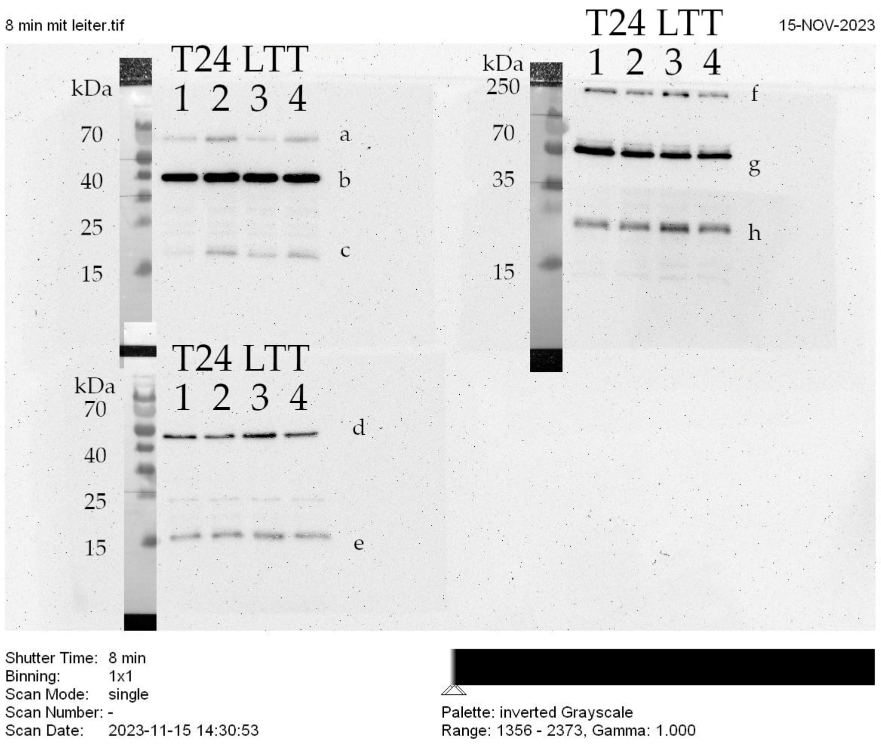

a 1: 2.50: 0.77: 2.04
c 1: 3.90: 2.00: 3.51
d 1: 0.62: 1.26: 0.83
e 1: 1.03: 1.17: 0.96

f 1: 0.54: 0.91: 0.57
g 1: 0.59: 0.57: 0.61
h 1: 0.82: 1.33: 1.03

Figure S14. Uncropped western blot, T24 LTT: blot at left top shows FOXO1 (a), β-Actin (b) and p21 (c), the blot below illustrates p53 (d) and survivin (e), the blot on the right-side displays BRD4 (f), c-Myc (g) and Bim (h), 48 h incubation. Order of samples left to right: control (1), entinostat (2), JQ1 (3), entinostat plus JQ1 (4). Densiometric analysis with ImageJ software of relative protein expression in relation to β-Actin is presented (a; c-h). Ratio of control was set to 1.

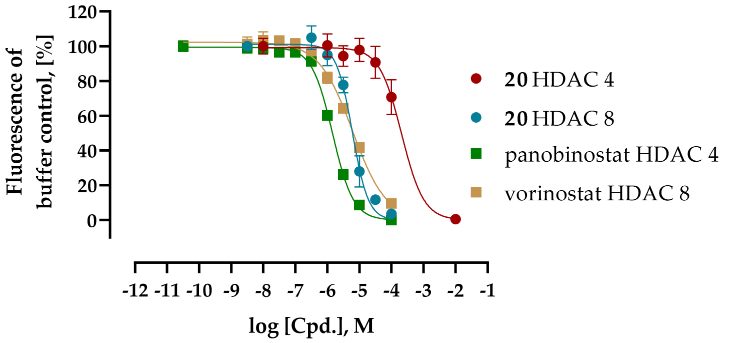

Figure S15. HDAC enzyme inhibitory activity of **20**, vorinostat and panobinostat.

Table S6. HDAC enzyme inhibitory activity of **20**, vorinostat and panobinostat. Data shown are IC_{50} values ± SD of at least two independent experiments, each carried out in duplicates. ND is not determined.

Cpd.	HDAC4 IC_{50} ± SD [µM]	HDAC8 IC_{50} ± SD [µM]
**20**	207 ± 50.0	6.03 ± 0.30
**vorinostat**	ND	6.13 ± 0.36
**panobinostat**	1.46 ± 0.04	ND

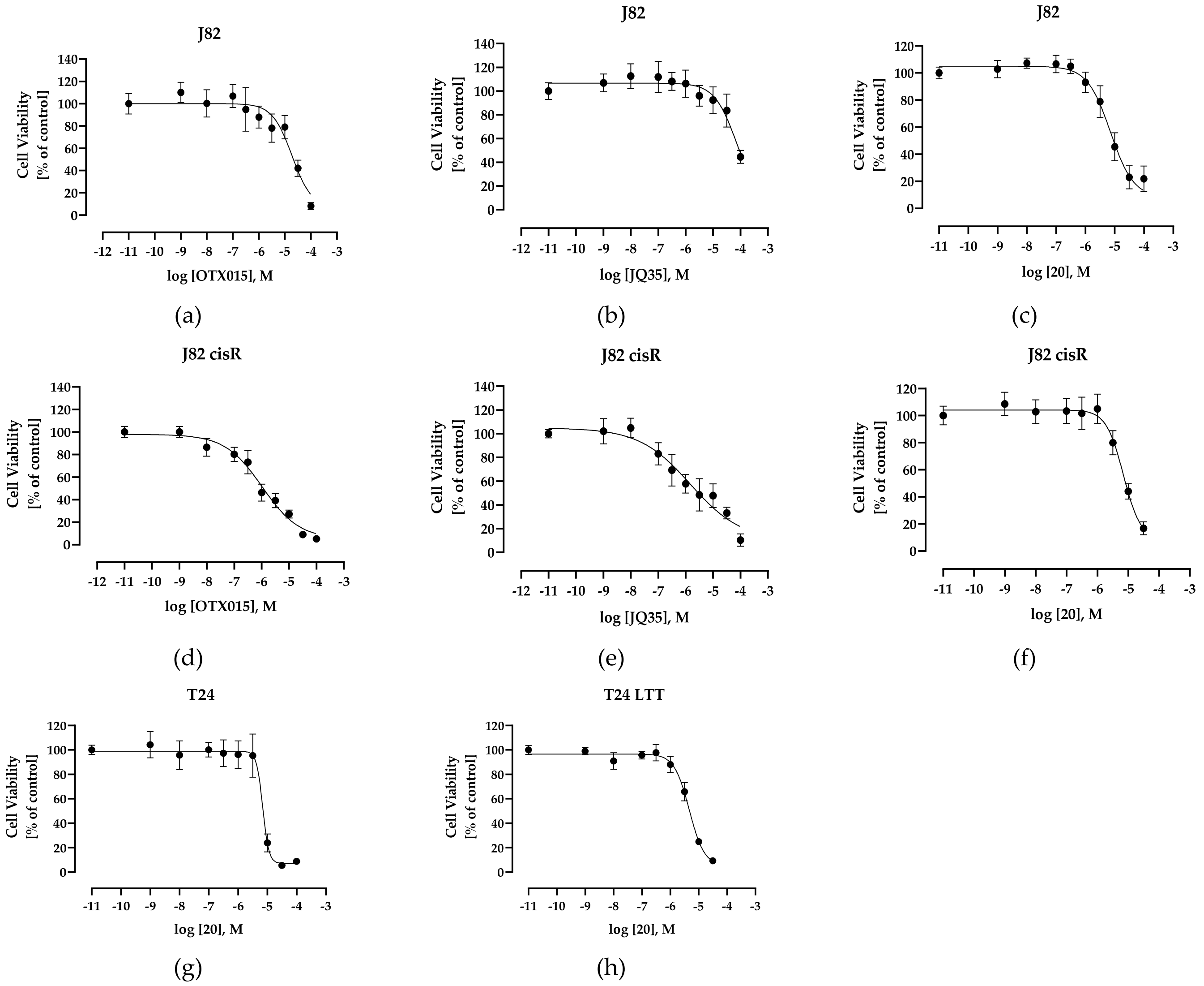

Figure S16. Concentration-effect-curves (MTT-Assay, 72 h) in J82 **(a)** OTX015, **(b)** JQ35, **(c) 20**; in J82 cisR **(d)** OTX015, **(e)** JQ35, **(f) 20**; in T24 **(g) 20** and in T24 LTT **(h) 20**. Data shown are the mean ± SD of at least three independent experiments, each carried out in triplicates.

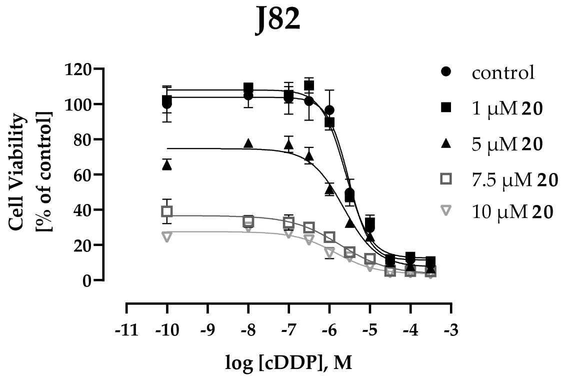

(a)

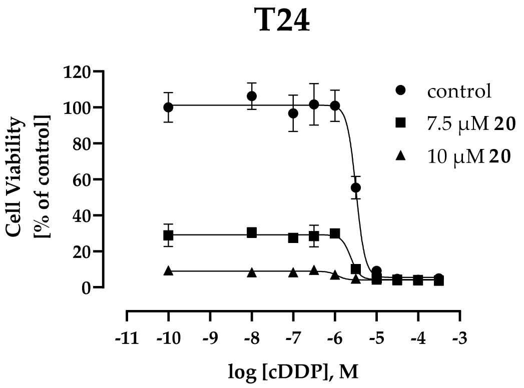

(b)

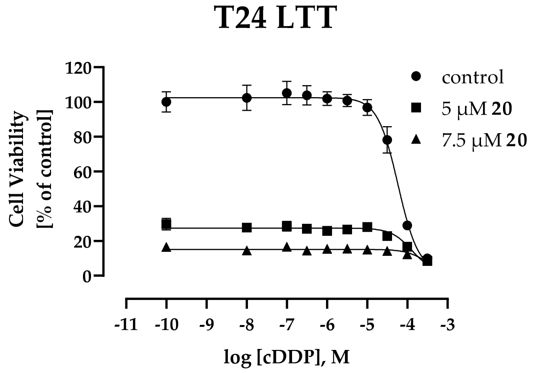

(c)

Figure S17. Concentration-effect-curves of cisplatin (MTT-Assay) of **(a)** J82; **(b)** T24; **(c)** T24 LTT. Cells were preincubated with **20** for 48 h followed by addition of cisplatin for another 72 h. Data shown are the mean of one representative experiment carried out in triplicates.

Table S7. IC_{50} values (MTT) of cisplatin. Cells were preincubated with entinostat and/or OTX015; JQ35 for 48 h followed by addition of cisplatin for another 72 h. Control was untreated for 48 h followed by addition of cisplatin for another 72 h. Data shown are the mean ± SD of at least three independent experiments, each carried out in triplicates.

**cell line**	**control**									
	**cisplatin IC_{50} ± SD [µM]**	**ENT**	**cisplatin IC_{50} ± SD [µM]**	**SF**	**OTX015**	**cisplatin IC_{50} ± SD [µM]**	**SF**	**JQ35**	**cisplatin IC_{50} ± SD [µM]**	**SF**
**J82**	3.02 ± 0.10	0.316 µM	1.93 ± 0.25	1.56	1 µM	1.36 ± 0.23	2.22	3 µM	2.18 ± 0.17	1.39
**J82 cisR**	7.80 ± 0.23	0.316 µM	4.29 ± 0.18	1.82	0.1 µM	4.17 ± 0.32	1.87	0.1 µM	4.88 ± 0.59	1.60
					0.3 µM	2.32 ± 0.15	3.36			

Table S8. IC_{50} values (MTT) of cisplatin. Cells were preincubated with **20** for 48 h followed by addition of cisplatin for another 72 h. Control was untreated for 48 h followed by addition of cisplatin for another 72 h. Data shown are the mean of one representative experiment carried out in triplicates. ND = not determined.

cell line	control	+ 1 µM 20		+ 5 µM 20		+ 7.5 µM 20		+ 10 µM 20	
	cisplatin IC_{50} ± SD [µM]	cisplatin IC_{50} ± SD [µM]	SF	cisplatin IC_{50} ± SD [µM]	SF	cisplatin IC_{50} ± SD [µM]	SF	cisplatin IC_{50} ± SD [µM]	SF
**J82**	2.98 ± 0.21	2.55 ± 0.26	1.17	2.26 ± 0.38	1.32	1.86 ± 0.56	1.60	1.43 ± 0.36	2.08
**J82 cisR**	7.64 ± 0.38	ND	ND	3.27 ± 0.63	2.33	1.47 ± 0.76	5.19	ND	ND
**T24**	3.23 ± 0.10	ND	ND	ND	ND	2.40 ± 0.22	1.34	1.15 ± 0.39	2.82
**T24 LTT**	58.7 ± 2.60	ND	ND	128 ± 18.4	0.46	285 ± 31.8	0.21	ND	ND

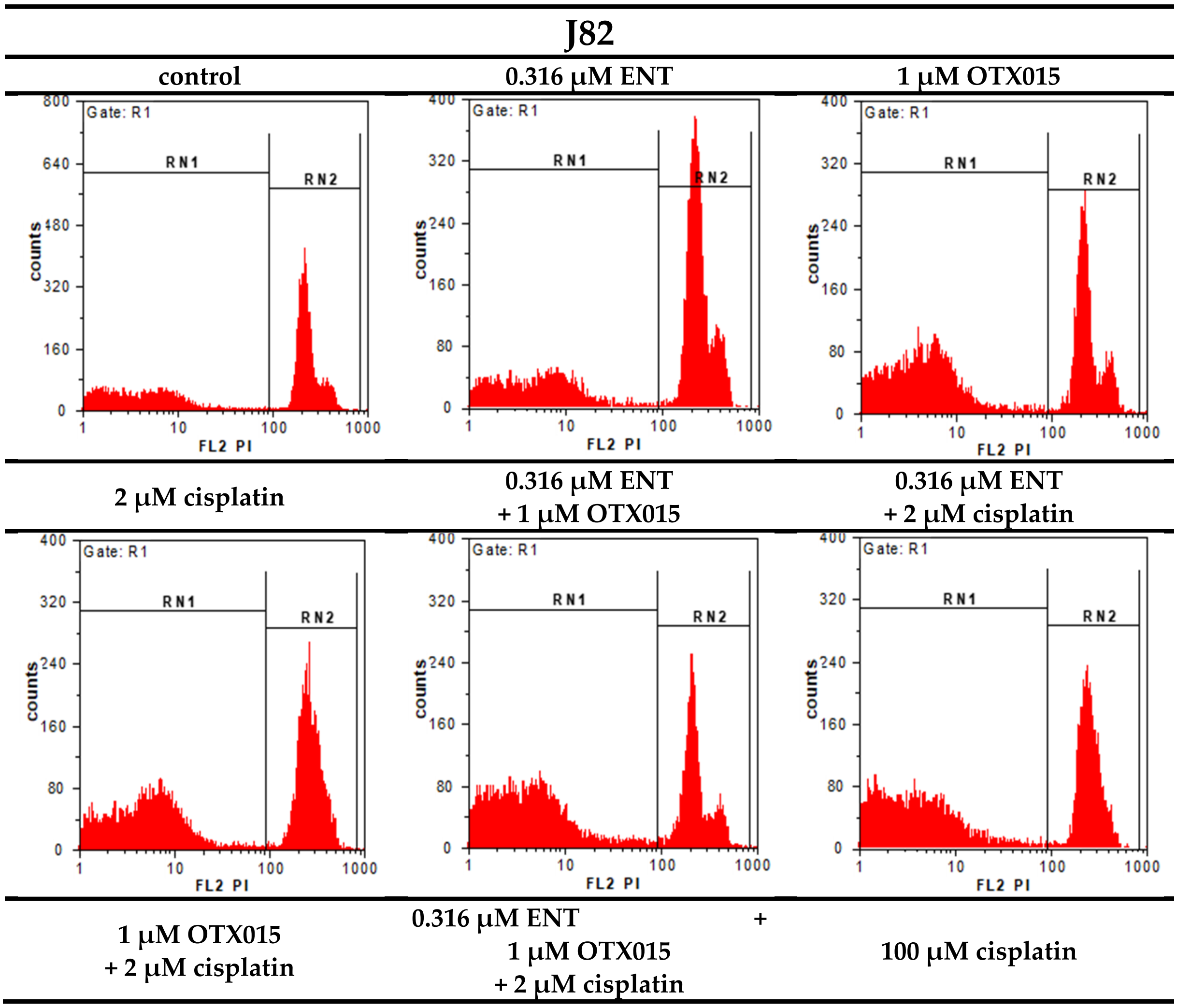

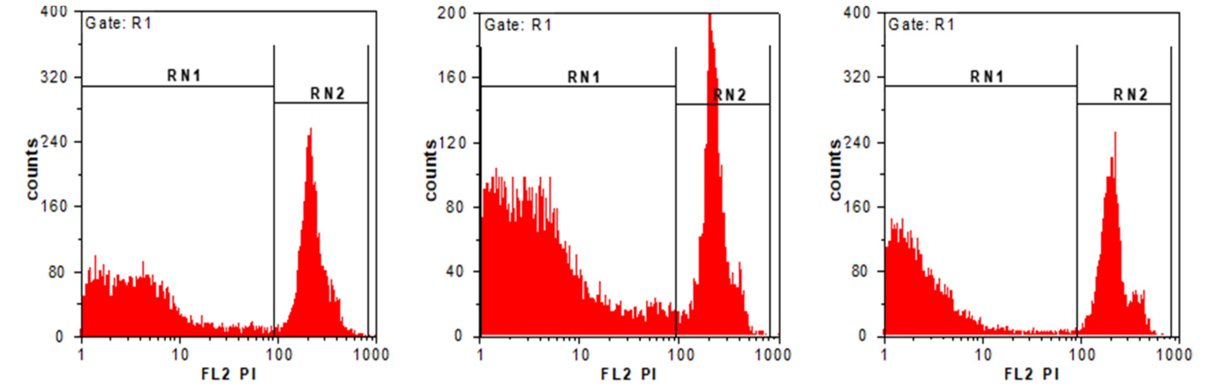

J82 cisR

control	0.316 μM ENT	0.1 μM OTX015

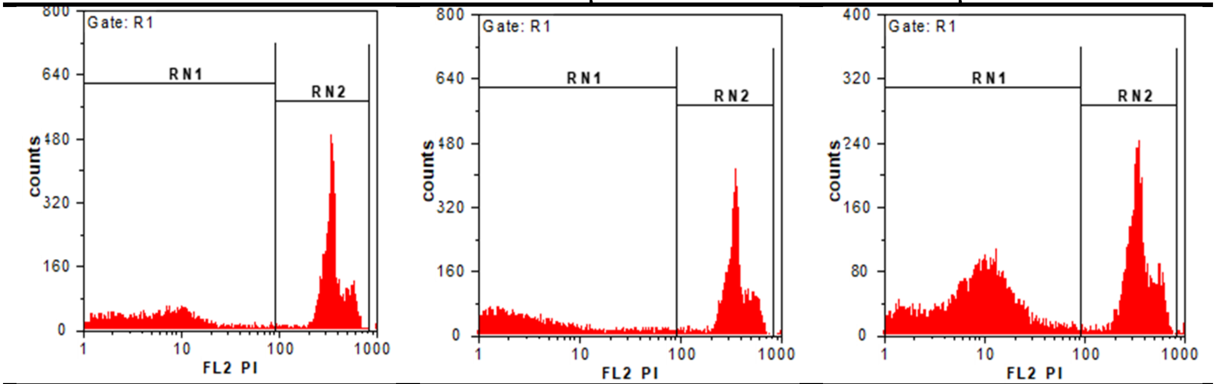

8 μM cisplatin	0.316 μM ENT + 0.1 μM OTX015	0.316 μM ENT + 8 μM cisplatin

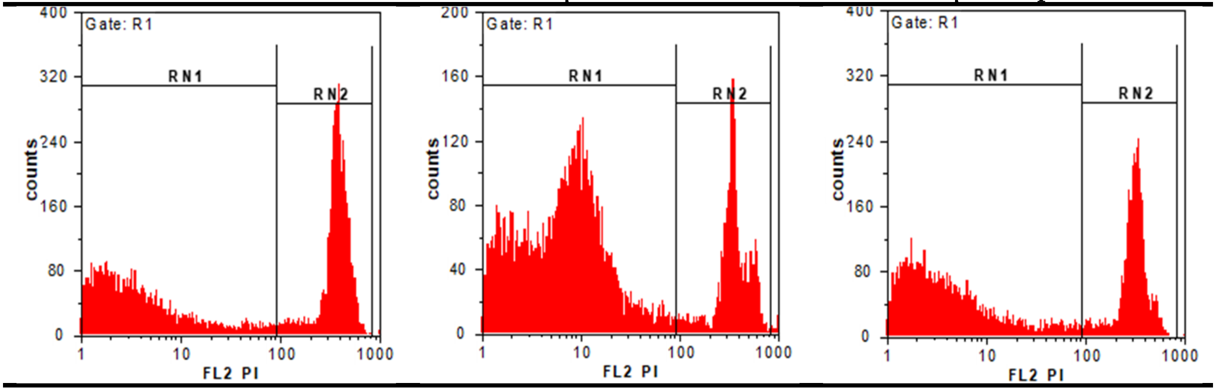

0.1 μM OTX015 + 8 μM cisplatin	0.316 μM ENT + 0.1 μM OTX015 + 8 μM cisplatin	100 μM cisplatin

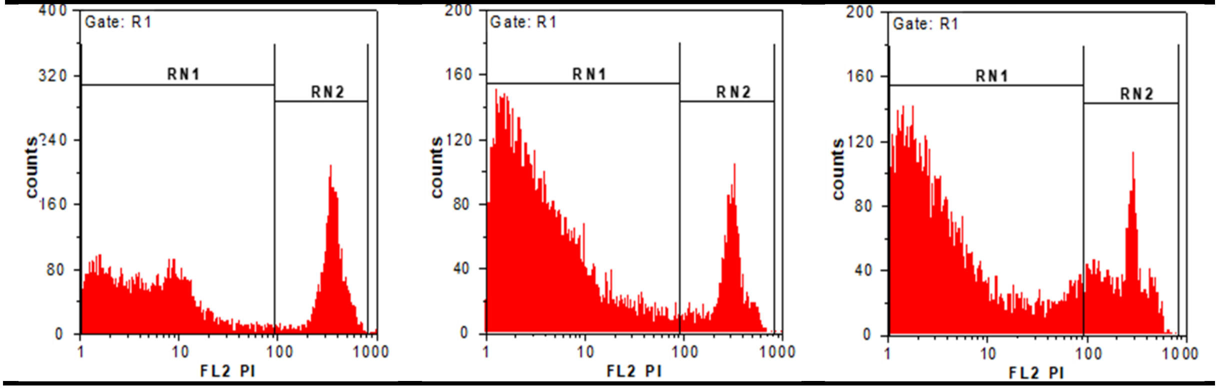

Figure S18. Representative cytometry flow images are shown for control, entinostat, OTX015, cisplatin and their combination treatments in J82 and J82 cisR. Cells were preincubated with entinostat and JQ1 for 48 h followed by addition of cisplatin for another 24 h. RN1 was defined as the subG1 fraction.

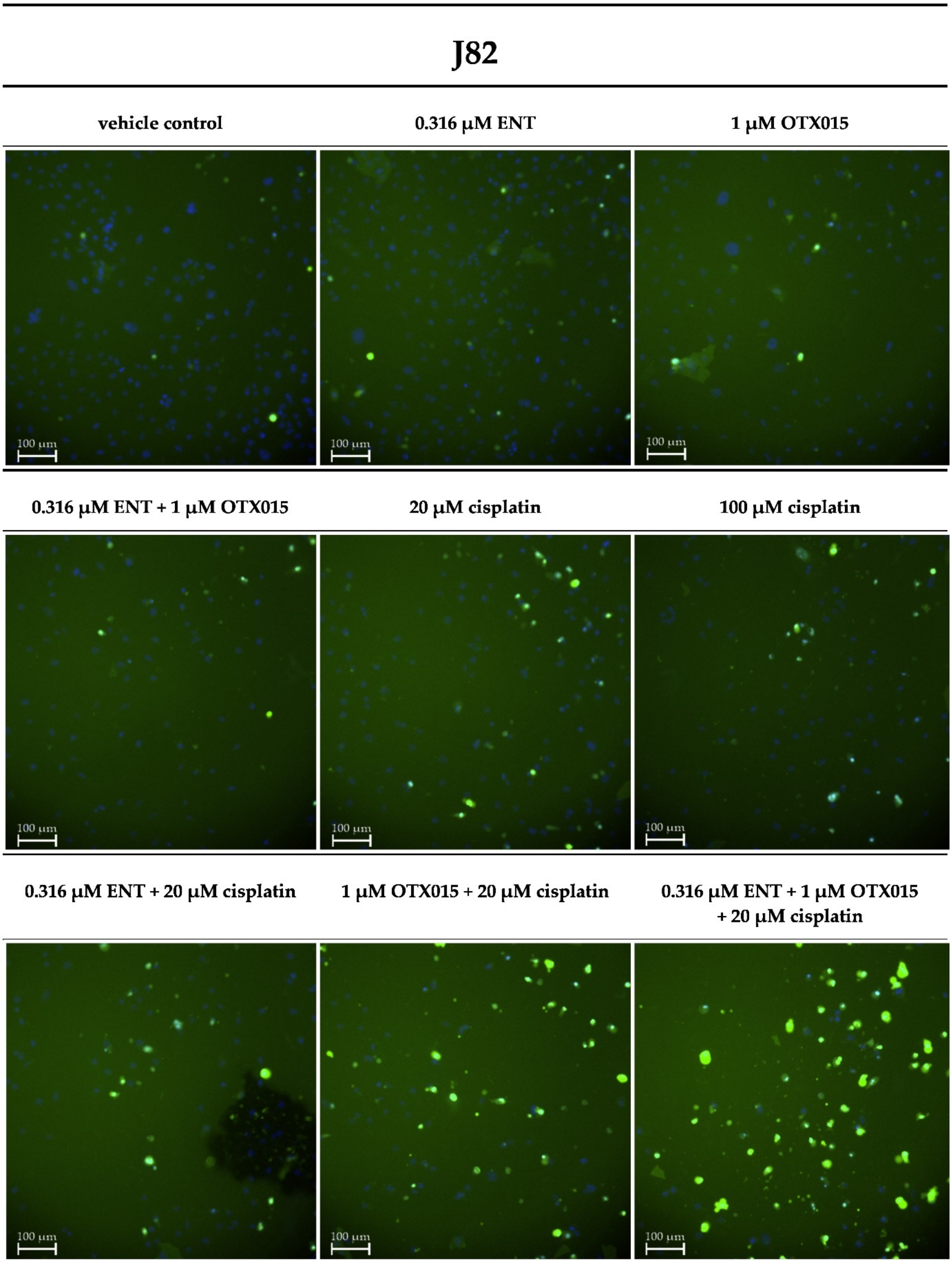

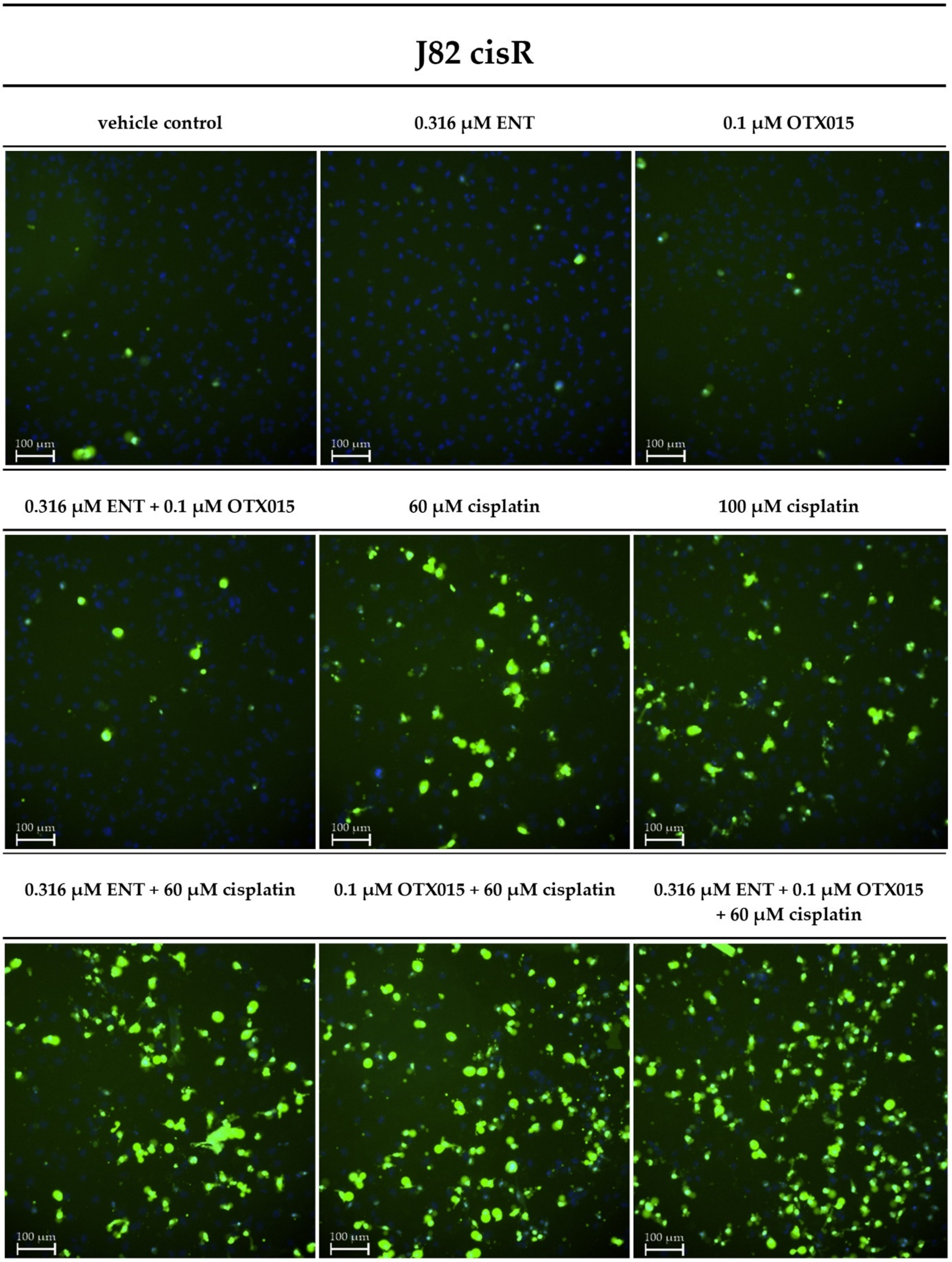
J82 cisR
vehicle control
0.316 μM ENT
0.1 μM OTX015
0.316 μM ENT + 0.1 μM OTX015
60 μM cisplatin
100 μM cisplatin
0.316 μM ENT + 60 μM cisplatin
0.1 μM OTX015 + 60 μM cisplatin
0.316 μM ENT + 0.1 μM OTX015 + 60 μM cisplatin
100 μm

(a)

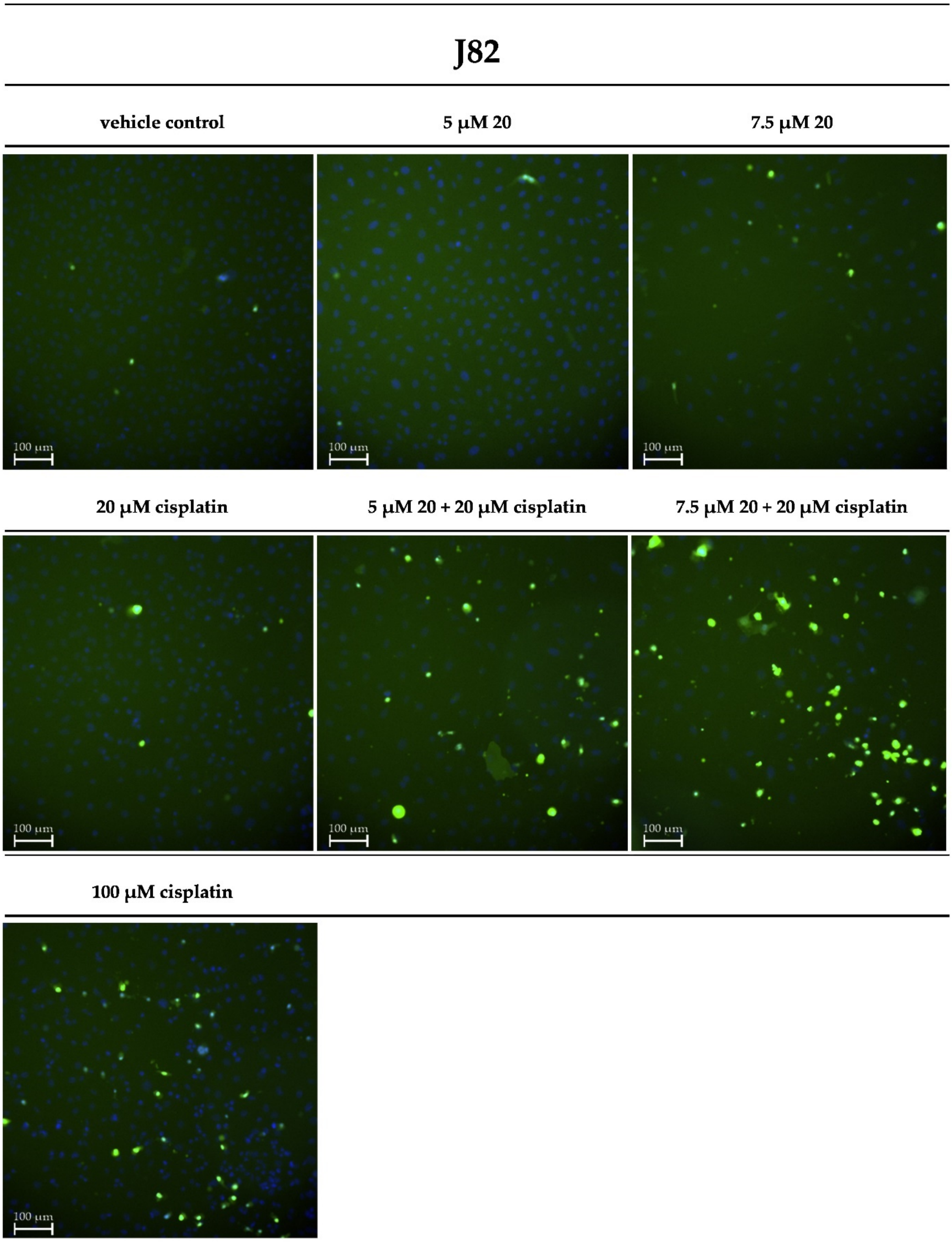
J82
vehicle control
5 µM 20
7.5 µM 20
20 µM cisplatin
5 µM 20 + 20 µM cisplatin
7.5 µM 20 + 20 µM cisplatin
100 µM cisplatin
100 µm

J82 cisR

vehicle control

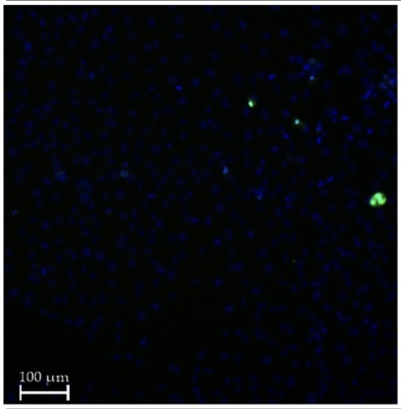

5 µM 20

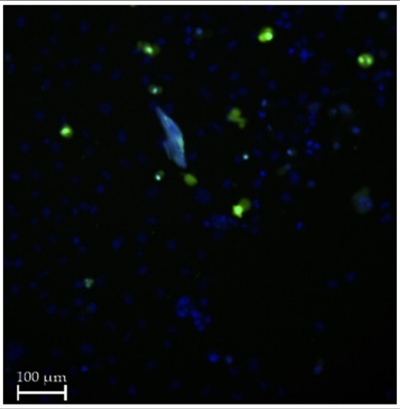

7.5 µM 20

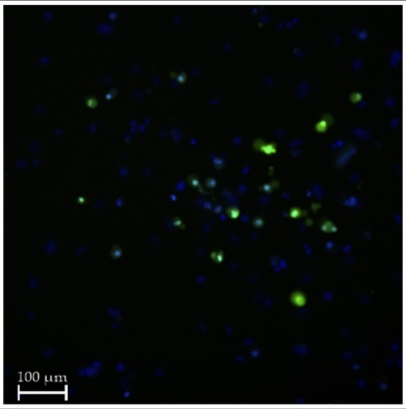

60 µM cisplatin

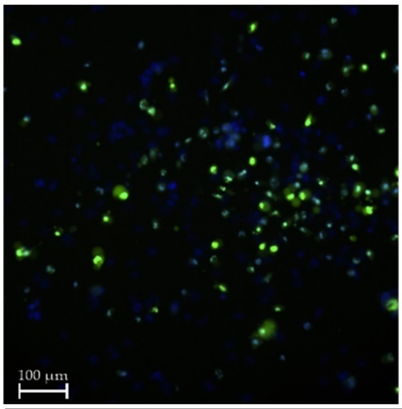

5 µM 20 + 60 µM cisplatin

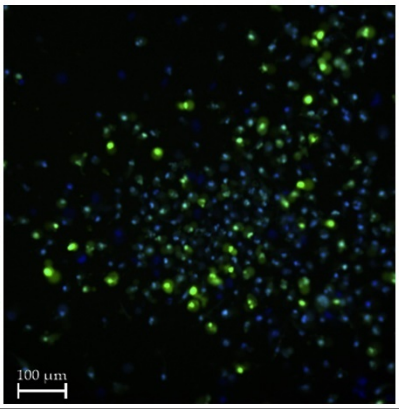

7.5 µM 20 + 60 µM cisplatin

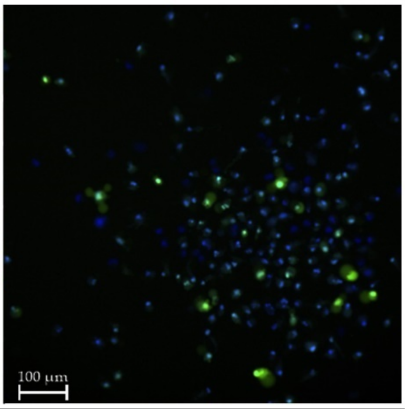

100 µM cisplatin

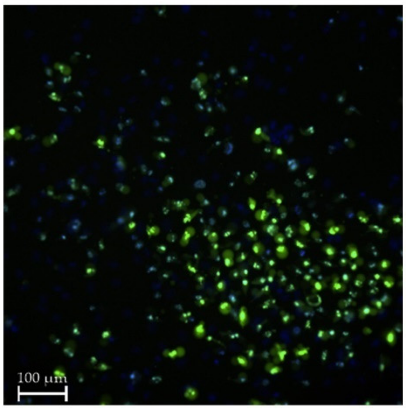

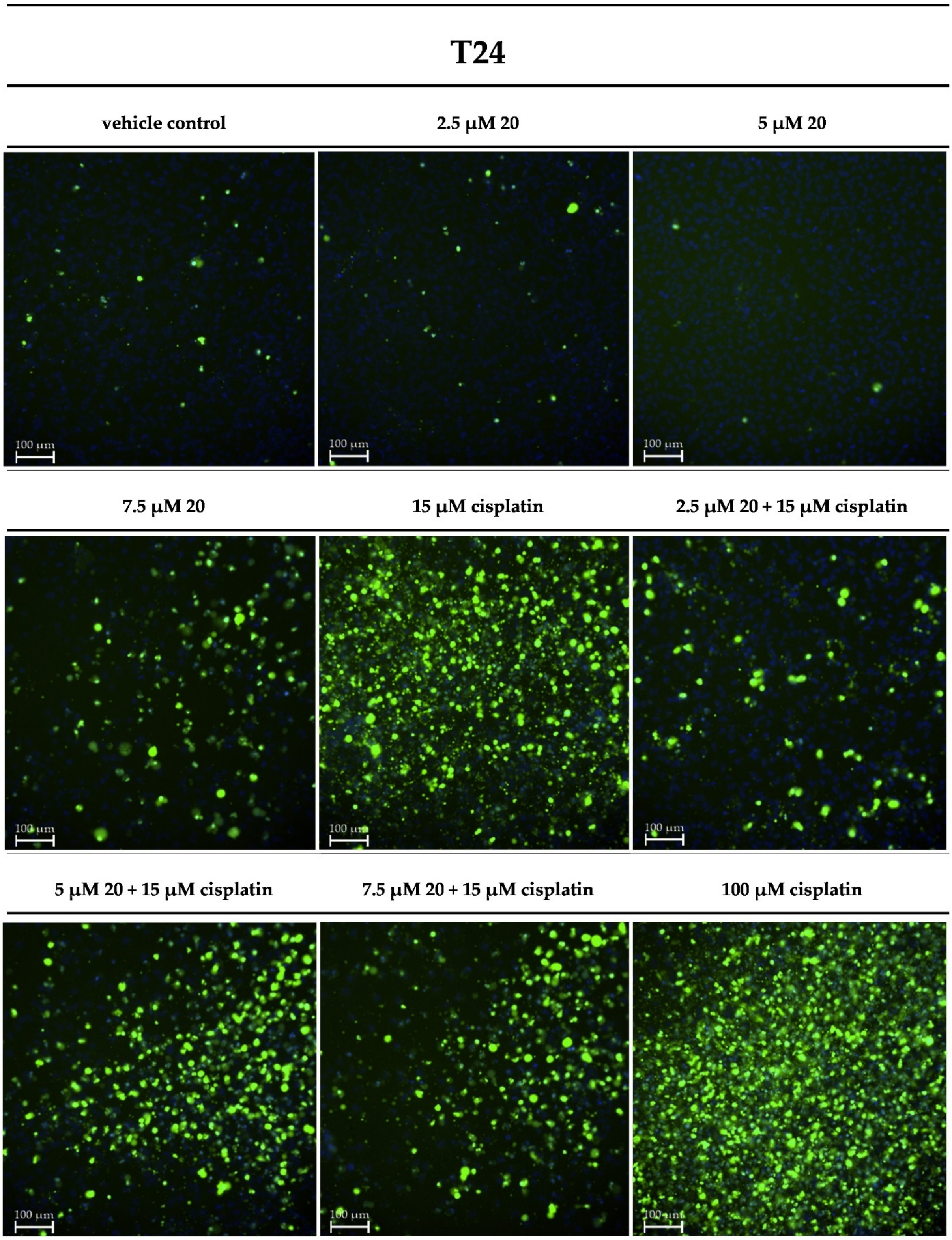
T24
vehicle control
2.5 µM 20
5 µM 20
7.5 µM 20
15 µM cisplatin
2.5 µM 20 + 15 µM cisplatin
5 µM 20 + 15 µM cisplatin
7.5 µM 20 + 15 µM cisplatin
100 µM cisplatin
100 µm

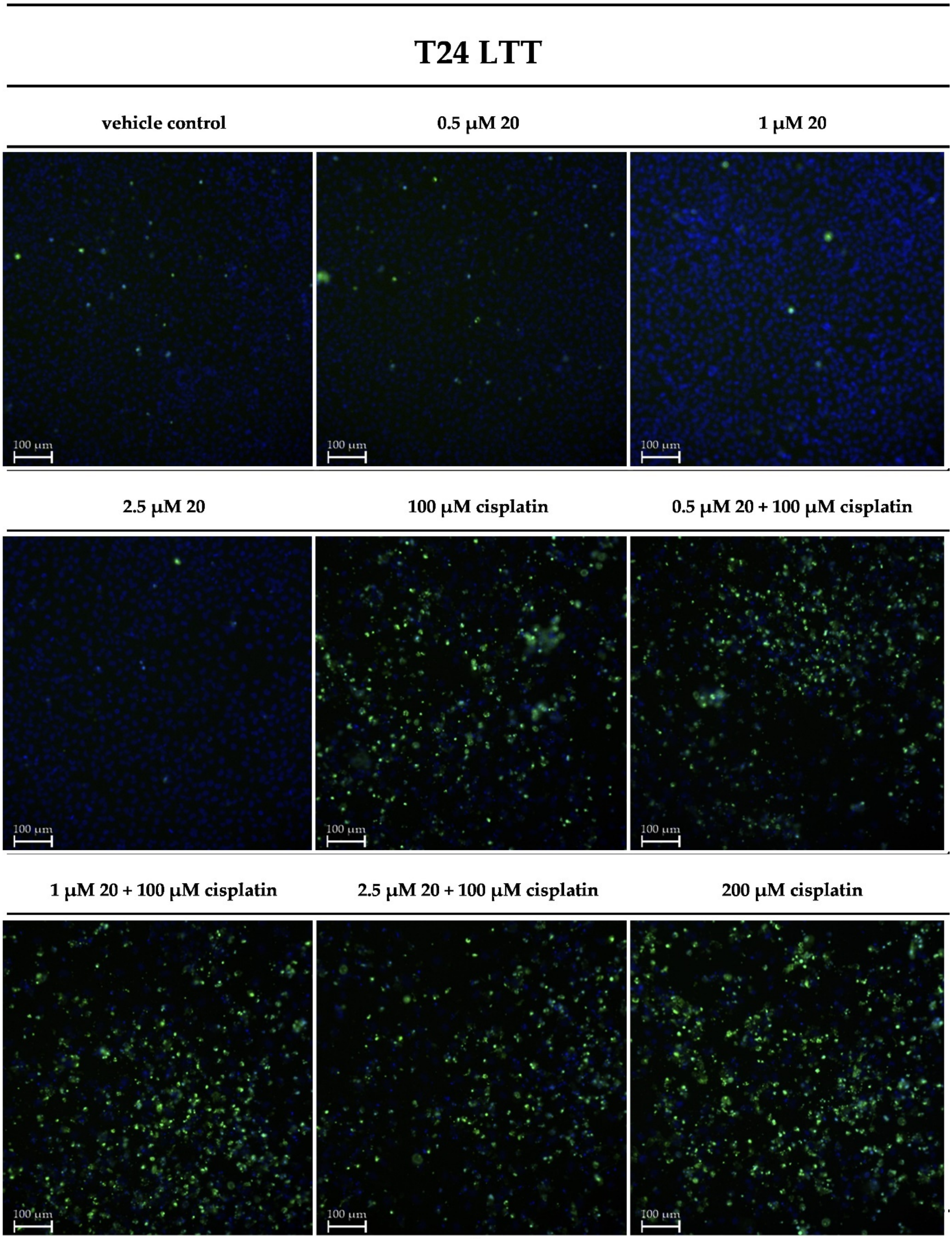

(b)

Figure S19. (a) Representative fluorescent imaging pictures (Thermofisher Arrayscan XTI) are shown for vehicle control, entinostat, OTX015, cisplatin and their combination treatments in J82, J82 cisR. Cells were preincubated with entinostat and OTX015 for 48 h followed by addition of cisplatin for another 24 h. **(b)** Representative fluorescent imaging pictures (Thermofisher Arrayscan XTI) are shown for vehicle control, **20**, cisplatin and their combination treatments in J82, J82 cisR, T24 and T24 LTT. Cells were preincubated with **20** for 48 h followed by addition of cisplatin for another 24 h (T24 LTT 48 h). Nuclei were stained using Hoechst 33342 (blue). Caspase 3/7 activation is presented in green.

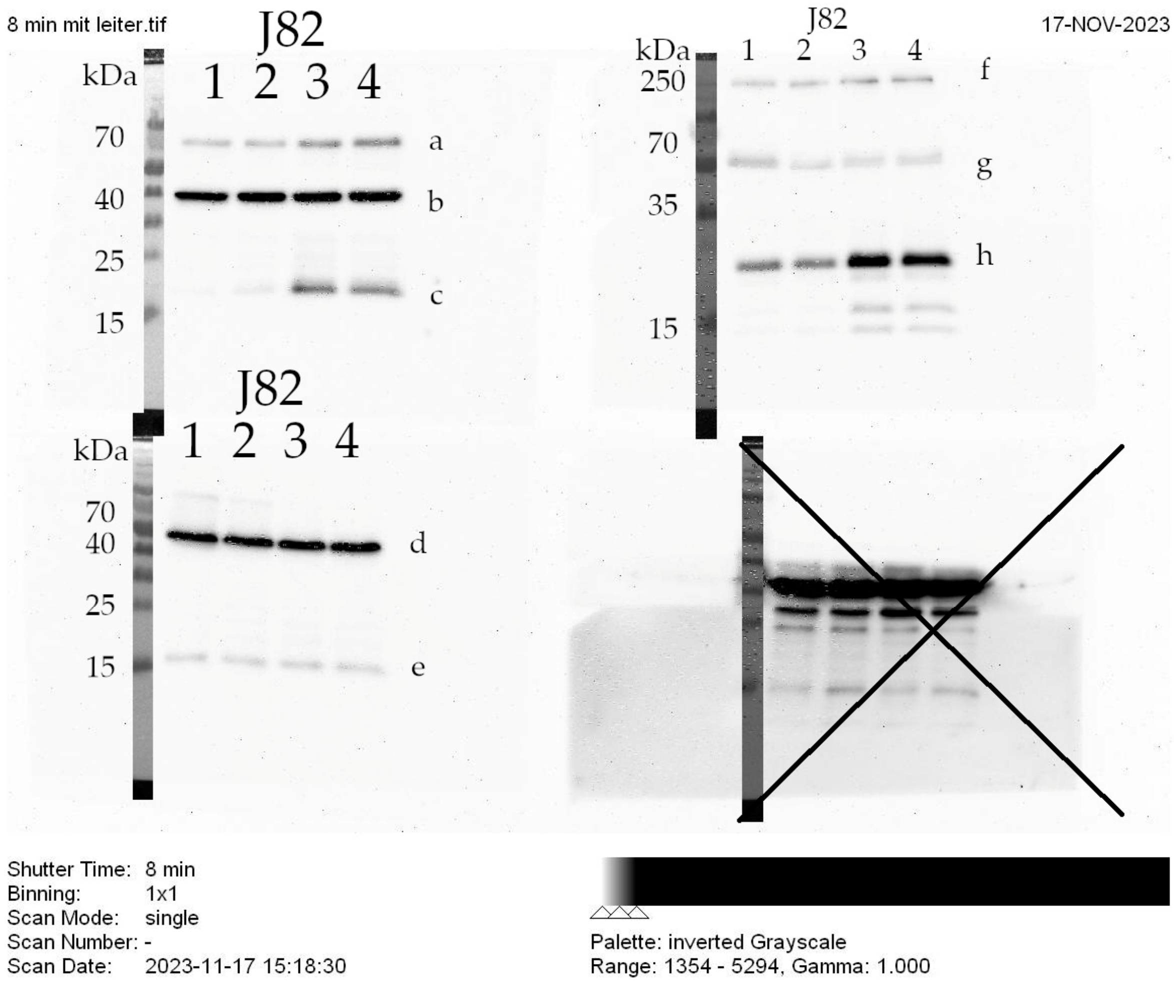

a 1: 0.87: 1.78: 2.32
c 1: 2.07: 24.5: 20.3
d 1: 0.94: 0.92: 0.90
e 1: 1.10: 1.21: 1.20

f 1: 0.88: 1.42: 1.49
g 1: 0.35: 0.43: 0.47
h 1: 0.80: 2.49: 2.34

Figure S20. Uncropped western blot, J82, blot at left top shows FOXO1 (a), β-Actin (b) and p21 (c), the blot below illustrates p53 (d) and survivin (e), the blot on the right-side displays BRD4 (f), c-Myc (g) and Bim (h), 48 h incubation. Order of samples left to right: control (1), entinostat (2), OTX015 (3), entinostat plus OTX015 (4). Densiometric analysis with ImageJ software of relative protein expression in relation to β-Actin is presented (a; c-h). Ratio of control was set to 1.

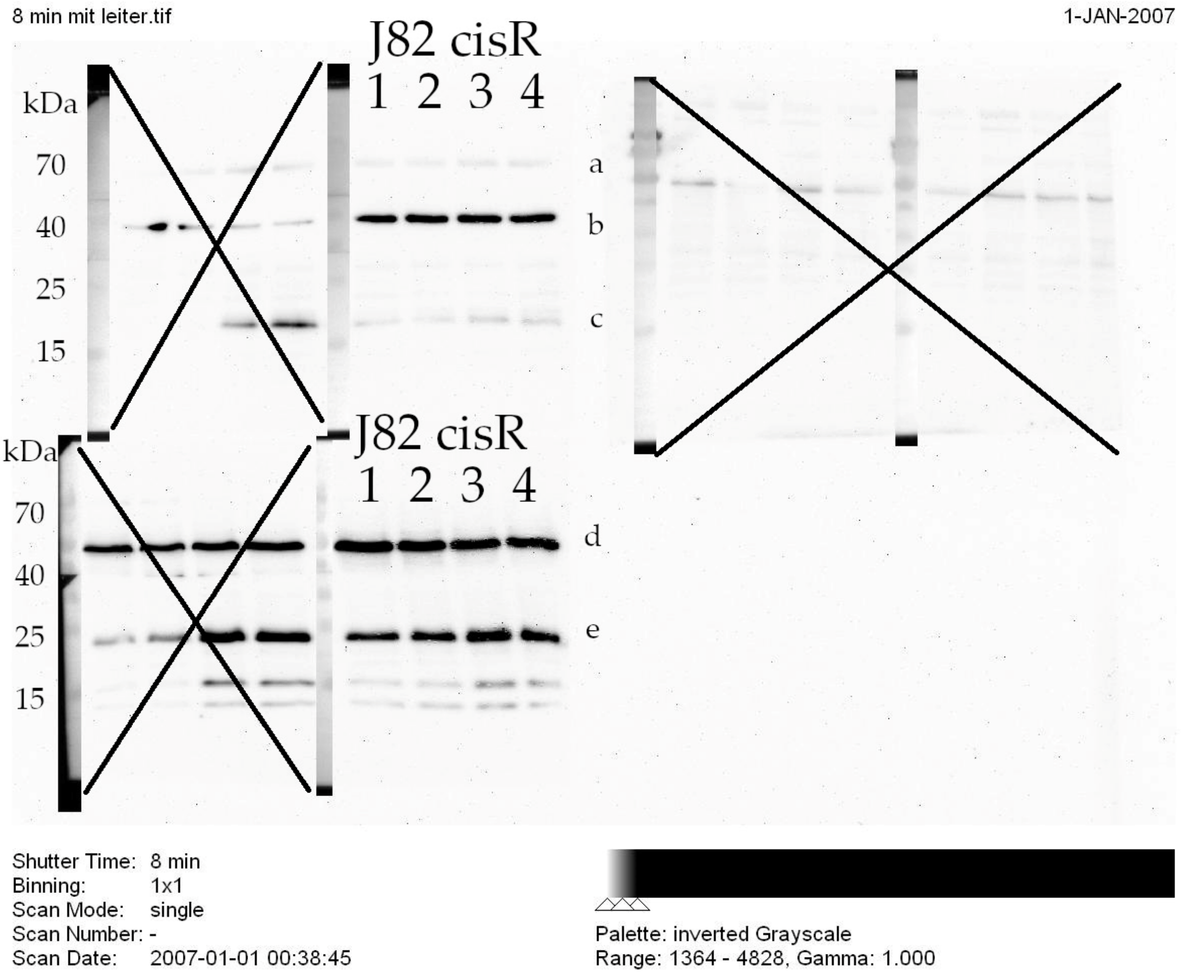

a 1: 0.87: 1.23: 1.11
c 1: 0.69: 1.48: 1.49
d 1: 0.72: 0.66: 0.65
e 1: 0.90: 1.10: 0.84

Figure S21. Uncropped western blot, J82 cisR, blot at left top shows FOXO1 (a), β-Actin (b) and p21 (c), the blot below illustrates p53 (d) and Bim (e), 48 h incubation. Order of samples left to right: control (1), entinostat (2), OTX015 (3), entinostat plus OTX015 (4). Densiometric analysis with ImageJ software of relative protein expression in relation to β-Actin is presented (a; c-e). Ratio of control was set to 1.

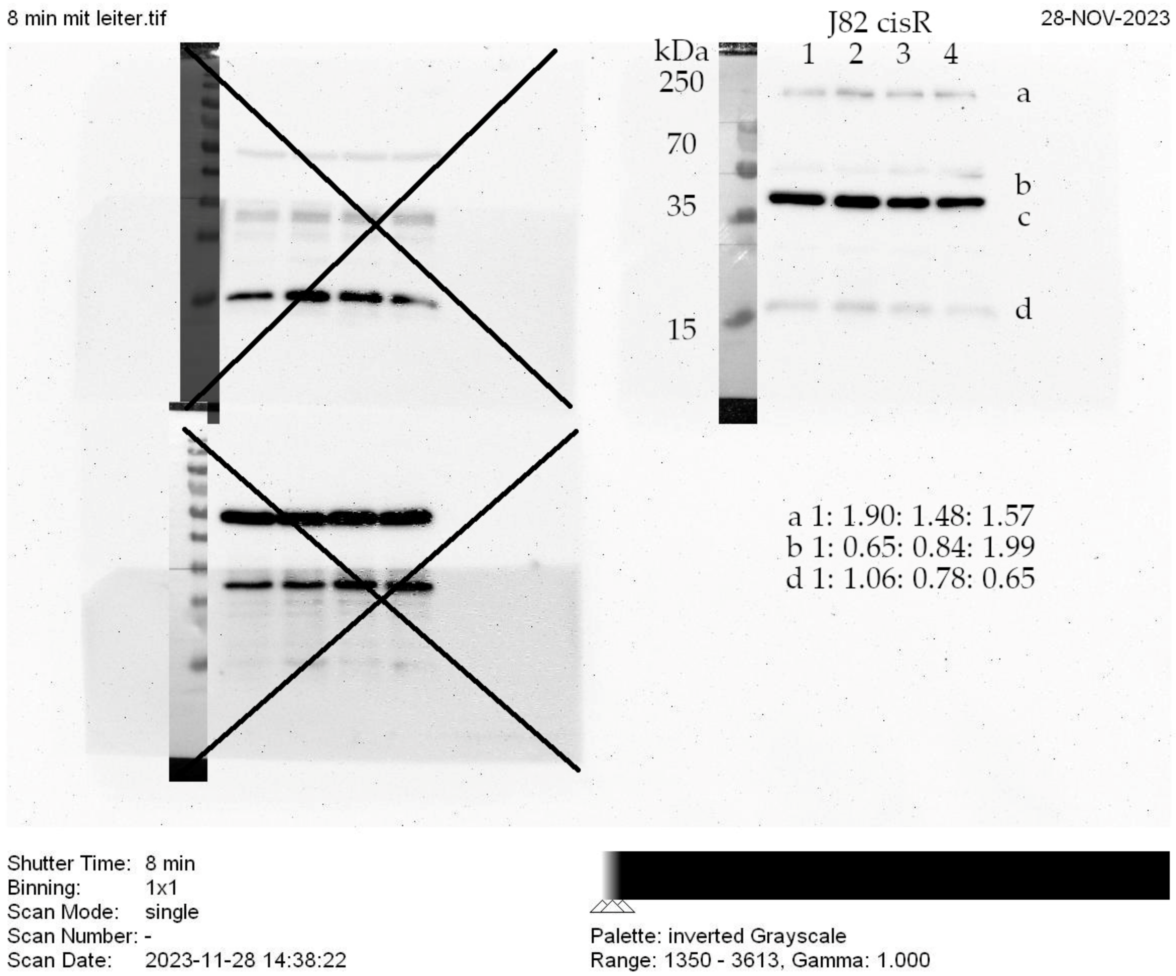

Figure S22. Uncropped western blot, J82 cisR, the blot on the right-side displays BRD4 (a), c-Myc (b), β-Actin (c) and survivin (d), 48 h incubation. Order of samples left to right: control (1), entinostat (2), OTX015 (3), entinostat plus OTX015 (4). Densiometric analysis with ImageJ software of relative protein expression in relation to β-Actin is presented (a-b; d). Ratio of control was set to 1.

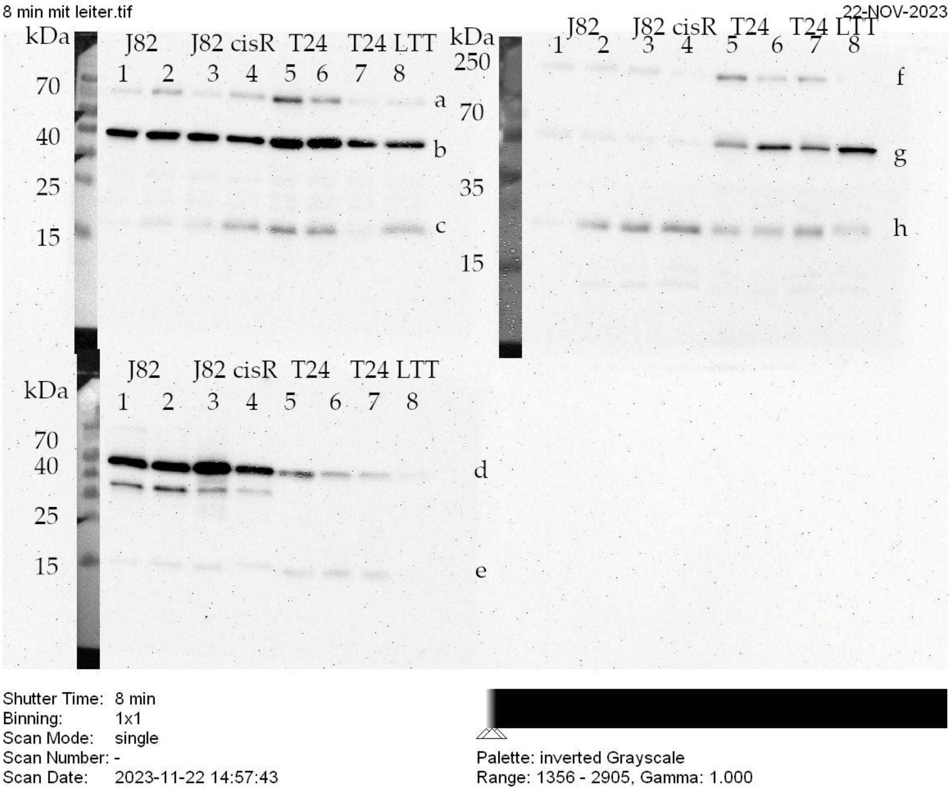

a 1: 2.50; 1: 2.76; 1: 0.48; 1: 2.20
c 1: 3.74; 1: 3.89; 1: 0.94; 1: 7.64
d 1: 0.90; 1: 0.52; 1: 0.33; 1: 0.32
e 1: 1.39; 1: 0.64; 1:1.26; 1: 0.11

f 1: 1.04; 1 :0.28; 1: 0.33; 1: 0.03
g 1: 0.63; 1: 1.11; 1: 1.91; 1: 1.93
h 1: 5.08; 1: 1.38; 1: 1.06; 1: 0.44

Figure S23. Uncropped western blot, J82, J82 cisR, T24 and T24 LTT, blot at left top shows FOXO1 (a), β-Actin (b) and p21 (c), the blot below illustrates p53 (d) and survivin (e), the blot on the right side displays BRD4 (f), c-Myc (g) and Bim (h), 48 h incubation. Order of samples left to right: control (1), 5 μM **20** (2), control (3), 5 μM **20** (4), control (5), 5 μM **20** (6), control (7), 5 μM **20** (8). Densiometric analysis with ImageJ software of relative protein expression in relation to β-Actin is presented (a; c-h). Ratio of control was set to 1.

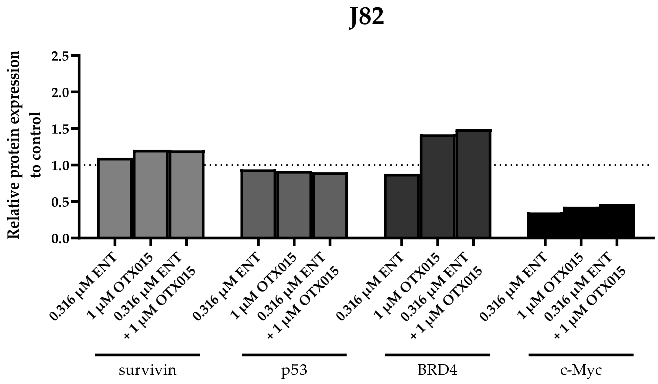

(a)

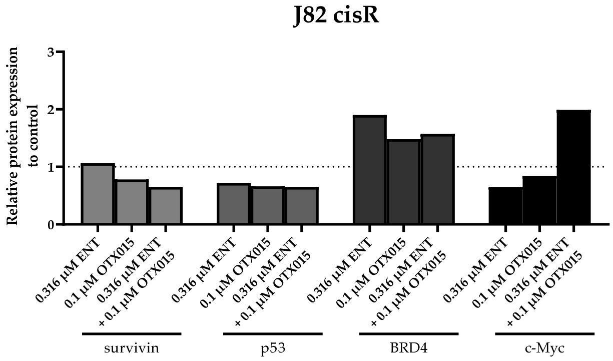

(b)

Figure S24. The relative protein expression of proteins of interest related to β-actin as loading control is shown in **(a)** J82, **(b)** J82 cisR. Ratio of control was set to 1, represented by the dashed line. Cells were incubated with entinostat and OTX015 for 48 h. The western blots were analyzed with ImageJ software. The data presented a representative blot out of at least two different protein samples.

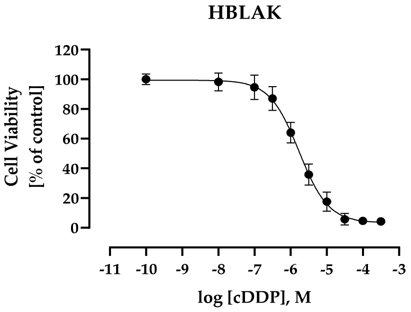

(a)

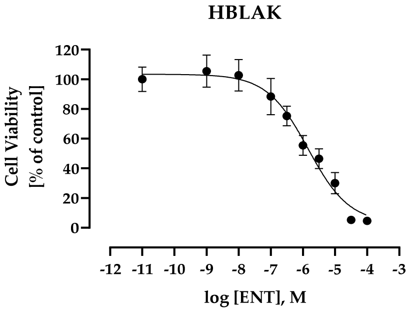

(b)

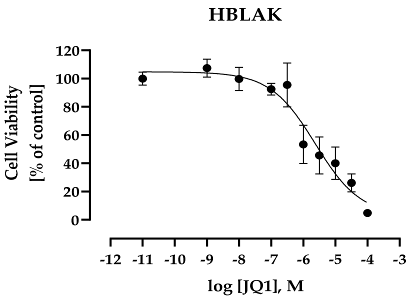

(c)

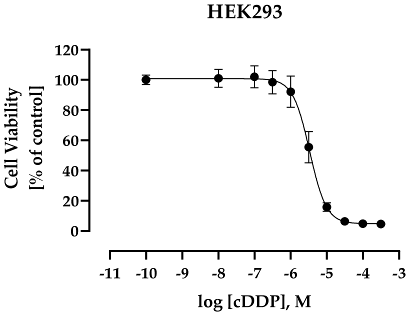

(d)

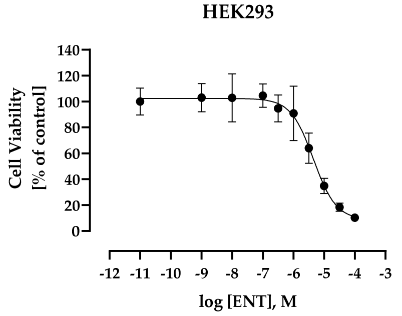

(e)

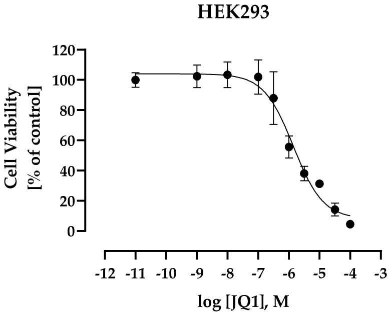

(f)

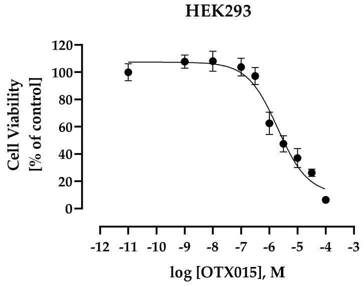

(g)

Figure S25. Concentration-effect-curves determined by 72 h MTT-Assay in **(a)-(c)** HBLAK and **(d)-(g)** HEK293. Data shown are the mean ± SD of at least three independent experiments, each carried out in triplicates.
